# Supplementary material for: Identification and Characterization of Jasmonic Acid Biosynthetic Genes in Salvia miltiorrhiza Bunge
Source: Int J Mol Sci. 2022 Aug 20;23(16):9384. doi: 10.3390/ijms23169384 (PMC9409215; doi:10.3390/ijms23169384)
Supplement: Supplementary file 1 [file ijms-23-09384-s001.zip › ijms-1852428-supplementary.pdf]

Supplementary Figure

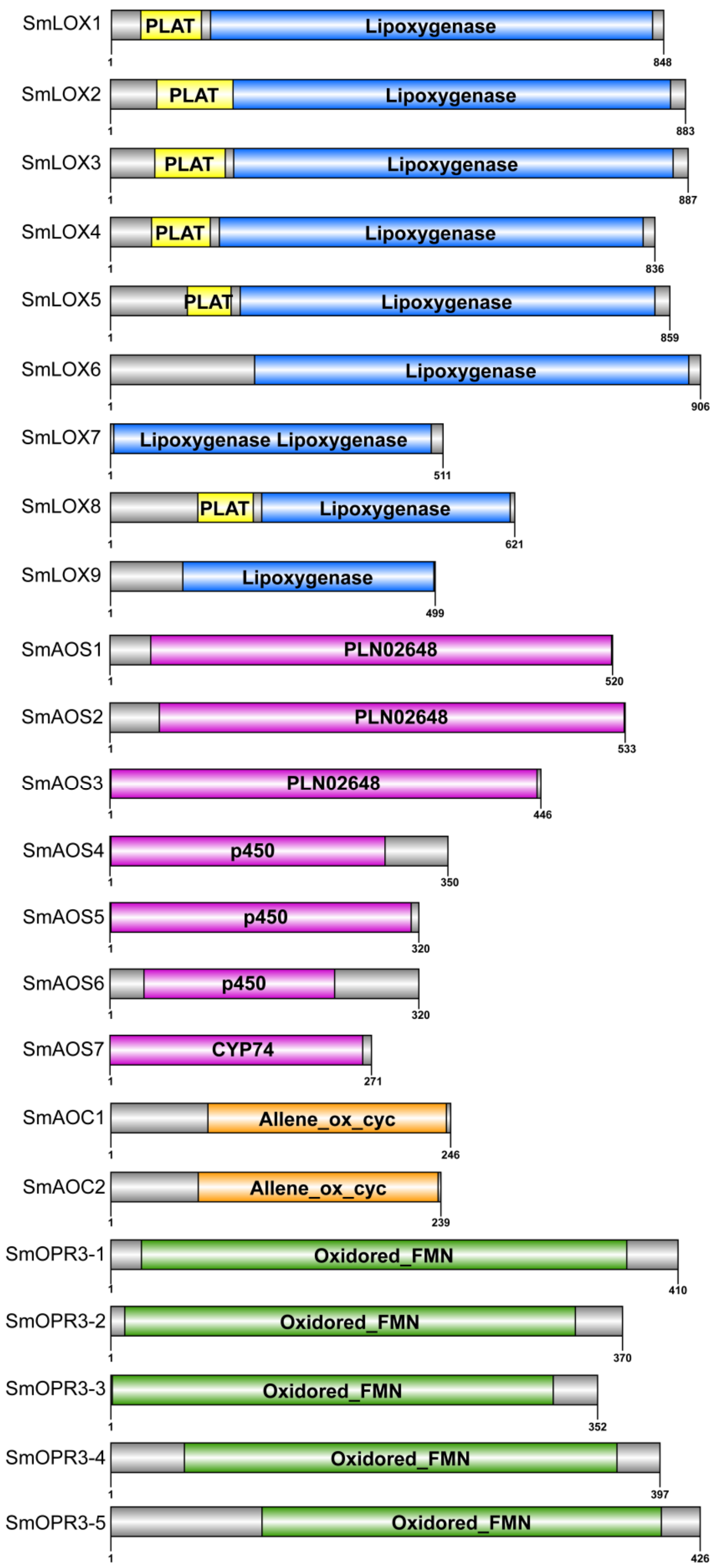

Figure S1. Conserved domains of JA synthesis-related proteins in *S. miltiorrhiza*. The name of the conservative domain is displayed in the middle of the box.

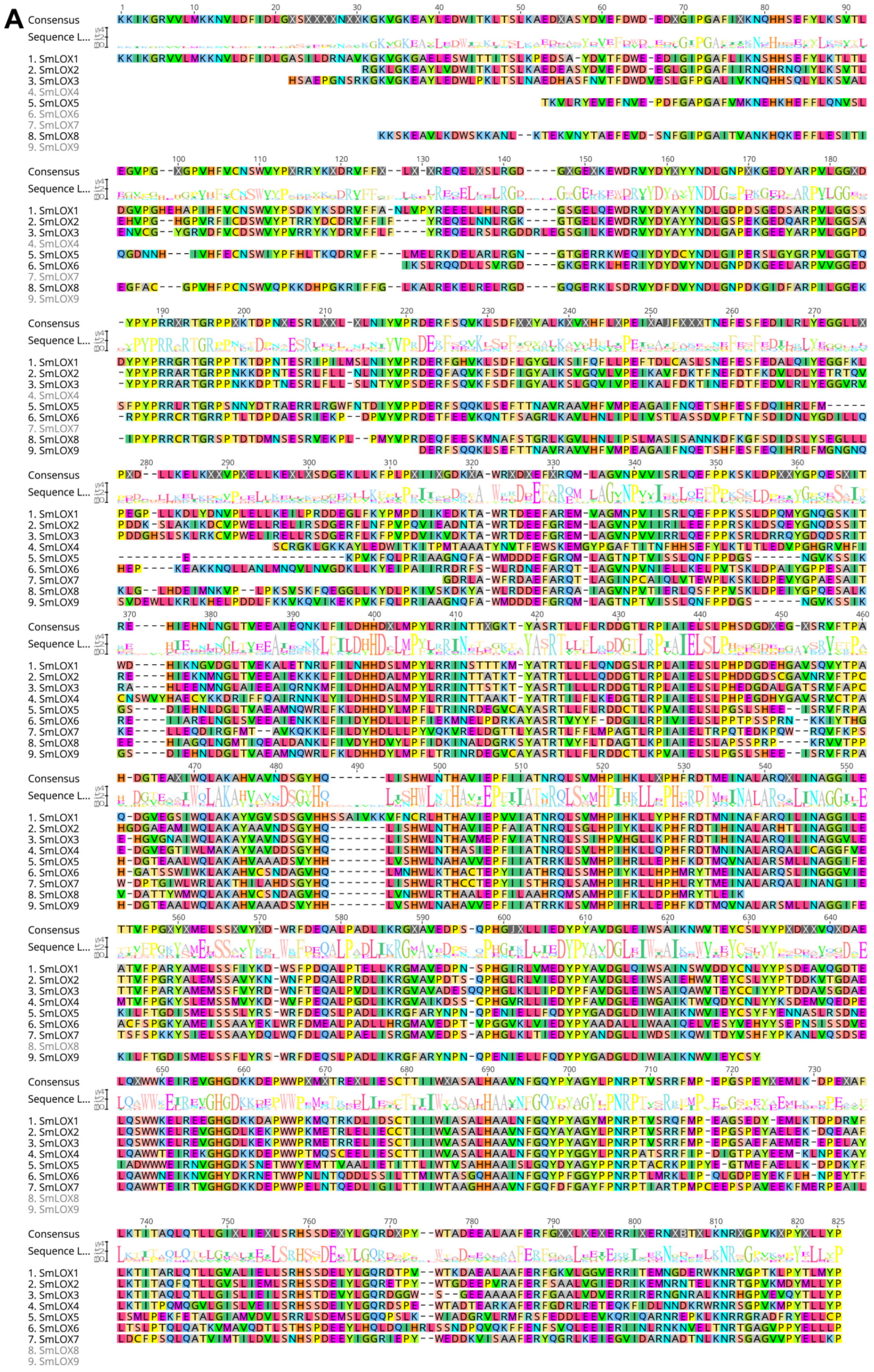
A
Consensus
Sequence L...
1. SmLOX1
2. SmLOX2
3. SmLOX3
4. SmLOX4
5. SmLOX5
6. SmLOX6
7. SmLOX7
8. SmLOX8
9. SmLOX9

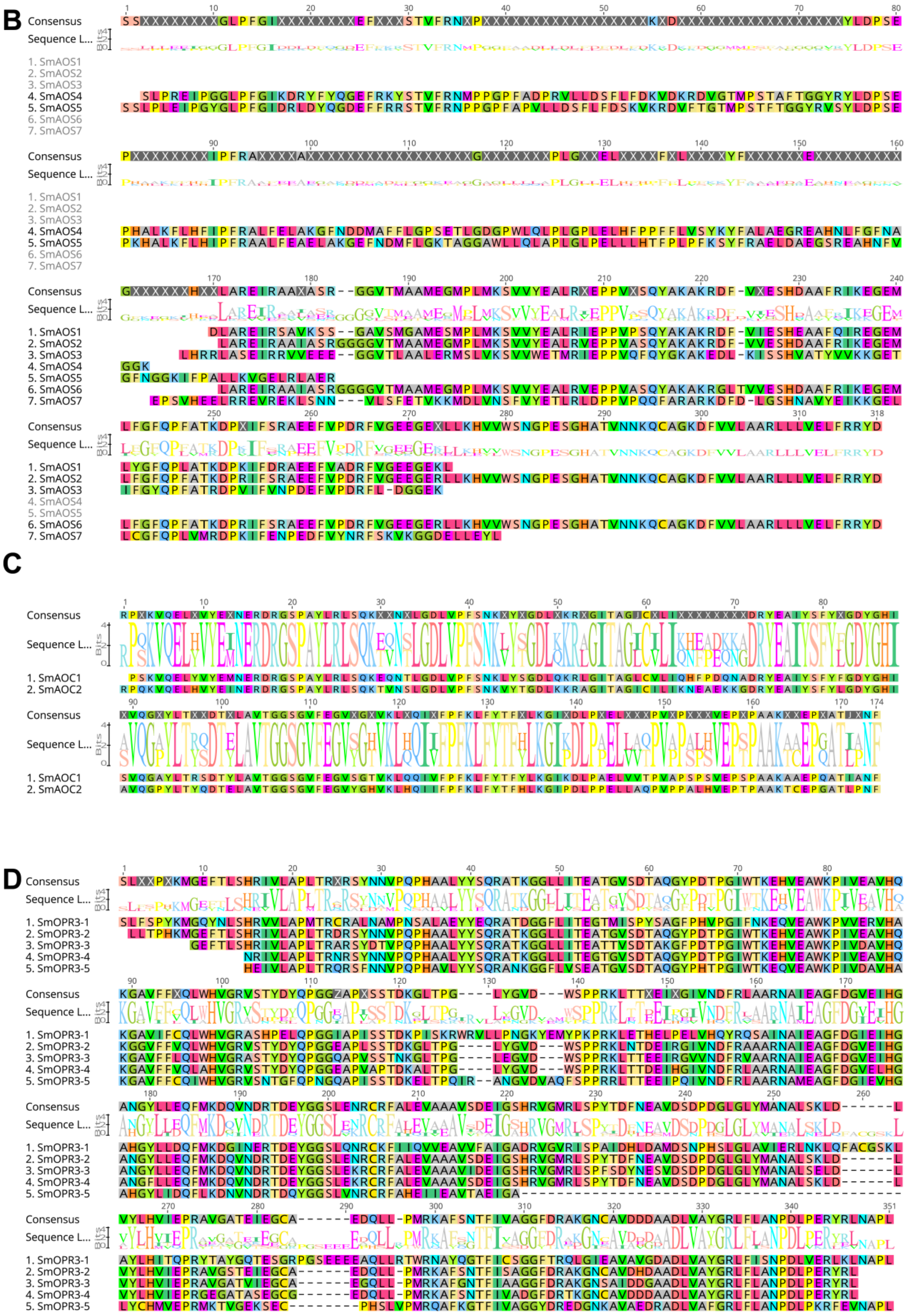

Figure S2. Comparison of amino acid sequences of JA synthesis-related proteins in *S. miltiorrhiza*. A, B, C and D are family multiple sequence alignments of SmLOXs, SmAOSs, SmAOCs and SmOPR3s, respectively.

Supplementary Table

Table S1. Predicted features of the JA gene family

Gene	Gene ID	Accession Number	Genome length(bp)	cDNA length(bp)	Protein length(aa)	Mw (Da) Molecular weight (average)	pI	Subcellular localization	Domain
SmLOX1	*SMil_00007321*	ON525660	6501	2547	848	96043.88	5.35	Cytoplasmic	Lipoxygenase/PLAT
SmLOX2	*SMil_00025931*	ON525661	4853	2649	883	93464.96	11.69	Cytoplasmic	Lipoxygenase/PLAT
SmLOX3	*SMil_00025932*	ON525662	4320	2661	887	100795.53	5.93	Cytoplasmic	Lipoxygenase/PLAT
SmLOX4	*SMil_00005983*	ON525663	4788	2508	836	95301.57	5.58	Cytoplasmic	Lipoxygenase/PLAT
SmLOX5	*SMil_00015717*	ON525664	4231	2577	859	98118.13	6.64	Cytoplasmic	Lipoxygenase/PLAT
SmLOX6	*SMil_00007851*	ON525665	3514	2718	906	102028.04	6.12	Chloroplast	Lipoxygenase
SmLOX7	*SMil_00027821*	ON525666	2945	1533	511	58056.15	5.42	Cytoplasmic	Lipoxygenase
SmLOX8	*SMil_00013199*	ON525667	2856	1863	621	70111.77	9.35	Cytoplasmic	Lipoxygenase/PLAT
SmLOX9	*SMil_00015715*	ON525668	2726	1497	499	56470.75	6.40	Cytoplasmic	Lipoxygenase
SmAOS1	*SMil_00004108*	ON525669	1560	1560	520	57936.59	8.88	Chloroplast	PLN02648
SmAOS2	*SMil_00002297*	ON525670	1599	1599	533	59642.73	9.30	Chloroplast	PLN02648
SmAOS3	*SMil_00027707*	ON525671	1421	1338	446	50097.57	6.07	Cytoplasmic	PLN02648
SmAOS4	*SMil_00024796*	ON525672	1050	1050	350	38237.66	7.05	Cytoplasmic	p450 super family
SmAOS5	*SMil_00028339*	ON525673	960	960	320	35556.58	6.26	Chloroplast	p450 super family
SmAOS6	*SMil_00002298*	ON525674	736	735	245	26644.74	9.24	Cytoplasmic	p450 super family
SmAOS7	*SMil_00024698*	ON525675	813	813	271	30635.10	5.71	Endoplasmic reticulum	CYP74
SmAOC1	*SMil_00024799*	ON525676	830	738	246	26771.31	8.66	Chloroplast	Allene_ox_cyc
SmAOC2	*SMil_00024374*	ON525677	896	720	239	25860.66	9.22	Chloroplast	Allene_ox_cyc
SmOPR3-1	*SMil_00009061*	ON525678	3078	1230	410	45451.88	8.19	Chloroplast	Oxidored_FMN
SmOPR3-2	*SMil_00009405*	ON525679	1371	1110	370	40661.51	5.03	Cytoplasmic	Oxidored_FMN
SmOPR3-3	*SMil_00009404*	ON525680	1546	1056	352	38476.22	5.23	Chloroplast	Oxidored_FMN
SmOPR3-4	*SMil_00009403*	ON525681	1661	1191	397	43766.2	5.10	Cytoplasmic	Oxidored_FMN
SmOPR3-5	*SMil_00024760*	ON525682	3009	1278	426	47422.04	6.64	Vacular membrane	Oxidored_FMN

Table S2. Primers for gene cloning of JA gene family

Gene	Sequence Type	Sequence (5'-3')
SmLOX1	F	ATGTTGAAAGGCATAGTAGACAC
	R	TCAAACAGAGGTACTATTAGGAATC
SmLOX2	F	ATGGGGATTTTCCCACCATG
	R	TCAAATAGATACACTATTTGGAATTCCT-
SmLOX3	F	ATGTCGTGTAATTGCTCAGAGATGT
	R	TCATATAGAGATGCTGTTGGGAATT
SmLOX4	F	ATGGATGAGCGCATCGTCAATG
	R	TTACATTGAGATGCTGTTGGGAATTCC
SmLOX5	F	ATGCCTTTAAAAACTTTGGAGTCC
	R	TCATATGGATATGCTGTTGGGAATT
SmLOX6	F	ATGCTTAGAGCTCAGGCCAG
	R	TCAGATGGAAATGCTGTTGG
SmLOX7	F	ATGACACCCGACGAAGGAGATAG
	R	CTAAATAGAGATGCTATTTGGGACACC
SmLOX8	F	TTGTCGAAGAGATCGAGCATGGCGC
	R	TCAAGCTCTGACGAGCCAACGCTTG
SmLOX9	F	TTGACGATGCCACTAACGAT
	R	CTAATTGTAAGAACAGTATTCTATCACC
SmAOS1	F	ATGGCTGCTTCCTCTTCTCTATCC
	R	TTAGAAAGTAGCCCGTTTGAGCG
SmAOS2	F	CACTCGGCAATGGCTGCTTCG
	R	CTAAAAGCTTGCCCGTTTTAACGACG
SmAOS3	F	ATGTCTTCAGTCGACGAGCTCCCAC
	R	CTATTTGGGGTTTGTGAGCGATTTGAT
SmAOS4	F	CAATGTCCACAGCCCAGCTTCCG
	R	TTAGCTCTCCCGTATTGGTACGGAACC
SmAOS5	F	ATGTCTTCAGTCGACGAGCTCCCACTG
	R	TCACGCCGCCCTCCTCCTCC
SmAOS6	F	ATGGCAATCCCGAAACCCGAAAG
	R	TCAGCAACCCCTCCCGCAATC
SmAOS7	F	ATGCTTGACAAATGGCTCGCCG
	R	TCACTTTGCCTTTTTGAGAGCAGTGA
SmAOC1	F	GCTTTCATCATTTATGGCTTTCACT
	R	TCAATTTGTGAAGTTAGCAATGGTG
SmAOC2	F	ATGGCAGCTTCATCCGCCTCTAC
	R	CTAATTGGTAAAGTTAGGGAGCGTGGC
SmOPR3-1	F	TTGGCGATCACGTCGAAATTGCAGC
	R	TCAAAGCCGCGATATGGTTGCAGGT
SmOPR3-2	F	ATGGCAGCTGAAGATACTGATGTT
	R	CTACACTATGCTAGCTTGTTGAAGAAAT

Gene	**Sequence Type**	**Sequence (5'-3')**
SmOPR3-3	F	ATGGGGGAATTCACACTCTCC
	R	CTATAATGCTTGTTGAAGAAATGGAT
SmOPR3-4	F	ATGGCAGCTGAAGACACTGATG
	R	CTACGGTTGTTGAAGAAATGGAT
SmOPR3-5	F	ATGCAGTTGATGGGCACTTCGAC
	R	TCAAGCAGTTTCATCCAGAAATGG

Table S3. Primers for qRT-PCR of JA gene family

Gene	**Sequence Type**	**Sequence (5'-3')**
SmLOX1	L	GACCCGCAAGGACCTCATCG
	R	ATGAATTGCCGGCTCACCGT
SmLOX2	L	ACACGCACGCCGTGATAGAG
	R	GAGTATTCCGCCGGCGTTGA
SmLOX3	L	TTTCCCGGCGCTTTGATCGT
	R	CCCACGAGTCGCAGACGAAA
SmLOX4	L	GTGCTTGGCATCTCCCTCGT
	R	CGTGCCTCTGTGTCTGCTGT
SmLOX5	L	ACGCAGGCTATCCACCAAACC
	R	CGGACAAGCGCCTTGACAGA
SmLOX6	L	TTGTGGGCGGTGAAGACAGG
	R	GCACAGCCTTGAGCCTTCCA
SmLOX7	L	GATGCGGAGTTCGCTCGACA
	R	TGACTCCGCCGGTCCATACA
SmLOX8	L	AGCCTTGCACCAAGCTCTCC
	R	GGCCAGTTGCCACATCCAGT
SmLOX9	L	CCCTGCGGAGGAGTCGATAGT
	R	TGAGCACCGTCTGTGTGACG
SmAOS1	L	CGACAACTCCTCGCCGATCC
	R	TTCATGCCGCCGAAGGTGTT
SmAOS2	L	TGCATCCGCTGCTCACTCTG
	R	GGCGGCAGCATGAAGTTGTG
SmAOS3	L	CGGACGAGTTCGTGCCTGAT
	R	CGCCGGACATTGCTTGTTGTC
SmAOS4	L	GCGACGAAGCGTGTCACAAC
	R	CACTTGATGAGCGCCGGGAA
SmAOS5	L	CGACACGTGGCTGTTTCTGC
	R	GGAGCAAATCCTCGACGAAATTAGGG
SmAOS6	L	GCGGACTTACGGTGGTGGAG
	R	GAAGGGCTGGAAGCCGAACA
SmAOS7	L	CAGTTCGCCCGAGCTCGAAA

Gene	Sequence Type	Sequence (5'-3')
	R	TGGCTGAAAGCCACACAGCA
SmAOC1	L	TTTCGGCGATTACGGCCACA
	R	TTGACCGTGCCAGACACACC
SmAOC2	L	TCGGCGATCTCGTGCCTTTC
	R	GGCCGTAGTCGCCCAAGTAG
SmOPR3-1	L	GAGCAAGTGGAGGCGTGGAA
	R	GATGCGAAGCTCGACCGACA
SmOPR3-2	L	GGTGGCGAAGCTCCACTCTC
	R	GCGCCGTGTATCTCGACTCC
SmOPR3-3	L	AGGCCTTCGGCAACACCTTC
	R	GGCGCATCCAAGCGATACCT
SmOPR3-4	L	GCACGTTGGCCGTGTCTCTA
	R	CCGTGGATTTCGTCGGTGGT
SmOPR3-5	L	GCCAAGGGTGCGGTCTTCTT
	R	CCGTTCGGCTGAAACCCTGT

Table S4. Sequences of the JA gene family

Gene	Sequence Type	Sequence (5'-3')
SmLOX1	Protein	MLKGIVDTLSGKNGEEKKKIKGRVVLMKKNVLDFIDLGASILDRNAVKGKVGKGAELESWITTITSLKPEDSAYDVTFDWEEDIGIPGAFLIKNSHHSEFYLKTLTLDGVPGHEHAPIH<br>FVCNSWVYPSDKYKSDRVFFANQAYLPSQTPAPLVPYREEELLHLRGDGSGELQEWDRVYDYAYYNDLGDPDSGEDSARPVLGGSSDYPYPRRGRTGRPPTKTDPNTESRIPILMSL<br>NIYVPRDERFGHVKLSDFLGYGLKSIFQFLLPEFTDLCASLSNEFESFEDALQIYEGGFKLPEGPLLKDLYDNVPLELLKEILPRDDEGLFKYPMPDIIKEDKTAWRTDEEFAREMVAGM<br>NPVIISRLQEFPPKSSLDPQMYGNQGSKITWDHIKNGVDGLTVEKALETNRLFILNHHDSLMPYLRRINSTTTKMYATRTLLFLQNDGSLRPLAIELSLPHPDGDEHGAVSQVYTPAQD<br>GVEGSIWQLAKAYVGVSDSGVHHSSAIVKKVFNCRLHTHAVIEPVVIATNRQLSVMHPIHKLLYPHFRDTMNINAFARQILINAGGILEATVFPARYAMELSSFIYKDWSFPDQALPTE<br>LLKRGMAVEDPNSPHGIRLVMEDYPYAVDGLQIWSAINSWVDDYCNLYYPSDEAVQGDTELQSWWKELREEGHGDKKDAPWWPKMQTRKDLIDSCTIIIWIASALHAALNFGQYP<br>YAGYMPNRPTVSRQFMPEAGSEDYEMLKTDPDRVFLKTITARLQTLLGVALIELLSRHSSDELYLGQRDTPVWTKDAEALAAFERFGKVLGGVERRITEMNGDERWKNRVGPTKLP<br>YTLMYPTSEEGLTGRGIPNSTSV-
	CDS	ATGTTGAAAGGCATAGTAGACACGCTGTCGGGAAAAAATGGGGAAGAGAAGAAGAAGATAAAAGGAAGAGTGGTGTTGATGAAGAAGAATGTTCTAGACTTCATCGATTTA<br>GGGGCATCCATTCTCGATCGAAATGCGGTGAAGGGGAAGGTGGGAAAAGGAGCAGAGCTAGAGAGCTGGATCACAACAATCACATCTCTGAAGCCTGAAGATTCAGCATAC<br>GACGTCACATTCGATTGGGAAGAAGACATCGGAATCCCAGGCGCATTCCTCATCAAGAACTCCCACCACAGCGAATTCTACCTCAAAACCCTAACCCTCGACGGCGTCCCCG<br>GCCACGAGCACGCCCCCATCCACTTCGTCTGCAACTCCTGGGTCTACCCCTCCGACAAGTACAAATCCGACCGCGTCTTCTTCGCCAATCAGGCCTATCTCCCCAGCCAAACTC<br>CGGCCCCACTTGTGCCCTACAGAGAGGAGGAGCTGCTGCATTTGCGTGGAGATGGGAGCGGAGAGTTGCAGGAATGGGACAGAGTGTATGACTACGCTTATTACAATGATTT<br>GGGCGACCCGGATAGCGGCGAGGATTCCGCCCGACCCGTCCTCGGTGGATCATCGGATTATCCGTATCCTCGTAGGGGAAGAACAGGCAGACCTCCTACTAAGACAGATCCC<br>AACACTGAGAGTAGGATCCCAATTCTTATGAGCTTGAACATCTACGTTCCAAGAGACGAGCGTTTTGGGCATGTGAAGCTGTCTGATTTTCTTGGTTATGGCCTTAAATCCATC<br>TTCCAGTTCCTCTTGCCTGAGTTTACAGACTTATGTGCCAGCCTCAGCAATGAGTTTGAGAGCTTCGAAGACGCACTTCAAATCTACGAGGGAGGGTTCAAGTTGCCGGAGGG<br>CCCTCTGCTCAAGGACTTATATGATAATGTCCCTTTGGAGCTGCTCAAGGAGATTCTTCCTAGAGATGATGAGGGCCTCTTCAAGTACCCGATGCCCGATATCATCAAAGAGG<br>ACAAGACTGCGTGGAGGACTGATGAAGAATTCGCGAGAGAGATGGTAGCAGGGATGAACCCTGTCATAATTAGTCGTCTGCAGGAGTTTCCTCCGAAGAGCAGCCTTGACCC<br>TCAAATGTATGGGAACCAGGGTAGCAAGATAACGTGGGATCATATCAAGAATGGAGTGGATGGACTCACTGTTGAAAAGGCGTTGGAGACGAACCGGCTGTTCATACTGAAT<br>CATCACGACTCACTGATGCCGTATCTGAGGAGGATCAACAGCACCACCACAAAAATGTACGCCACAAGGACTCTGCTTTTCTTGCAGAATGACGGGAGTTTGAGGCCGTTGG<br>CGATTGAGCTCAGCCTCCCTCATCCTGATGGGGATGAGCACGGTGCCGTGAGTCAGGTGTACACTCCTGCTCAAGACGGCGTTGAGGGTTCGATTTGGCAGCTGGCCAAGGCC

Gene	Sequence Type	Sequence (5'-3')
		TATGTTGGAGTCAGTGATTCTGGAGTTCATCACTCATCAGCCATTGTAAAGAAAGTGTTTAACTGCAGGTTGCATACACATGCTGTGATAGAGCCGGTGGTGATTGCAACGAA<br>CAGACAACTGAGCGTGATGCACCCCATTCATAAGCTTCTGTATCCTCACTTCCGCGACACGATGAACATCAATGCTTTCGCGCGGCAAATCTTGATCAATGCCGGAGGGATTC<br>TGGAGGCCACCGTCTTCCCTGCTCGATACGCCATGGAGCTCTCTTCCTTCATATACAAGGACTGGAGTTTCCCCGATCAGGCACTCCCTACTGAATTACTCAAGAGAGGCATG<br>GCGGTGGAAGATCCAAACTCCCCACACGGAATCCGCCTGGTGATGGAGGACTATCCATATGCAGTGGACGGCCTCCAAATCTGGTCGGCCATCAACTCGTGGGTGGATGACT<br>ACTGCAACCTGTACTATCCCTCAGACGAGGCCGTGCAGGGCGACACCGAGCTACAATCGTGGTGGAAGGAGCTCCGGGAGGAGGGGCACGGCGACAAGAAGGACGCGCCGT<br>GGTGGCCTAAGATGCAGACCCGCAAGGACCTCATCGACAGCTGCACCATCATCATCTGGATCGCGTCCGCGCTGCACGCAGCGCTCAACTTCGGGCAGTACCCCTACGCCGG<br>CTACATGCCCAACCGCCCCACGGTGAGCCGGCAATTCATGCCCGAGGCCGGCAGCGAGGACTACGAGATGCTGAAGACGGACCCCGACAGGGTGTTCTTGAAGACCATCACG<br>GCGAGGCTGCAGACGCTGCTGGGCGTCGCCCTGATCGAGCTCCTGTCGAGGCATTCCTCGGACGAGCTGTATCTCGGGCAGAGGGACACTCCGGTGTGGACCAAGGACGCGG<br>AGGCGCTGGCGGCGTTCGAGAGGTTTGGGAAGGTGTTGGGTGGAGTGGAGAGGAGGATCACGGAGATGAATGGTGACGAGAGATGGAAGAATAGAGTTGGGCCTACGAAG<br>CTGCCTTATACGTTGATGTATCCAACGAGTGAGGAAGGATTGACTGGTAGAGGGATTCCTAATAGTACCTCTGTTTGA
SmLOX2	Protein	MGIFPPCDSSEVMEKLLGSVCGKTRDDPATERSSKIKGSVVLMKKNAMDSTDFGASLVDRVYEIFGRGIILQLVSAQHFDPANPKRGKLGKEAYLVDWITKLTSLKAEDEASYDVEF<br>DWDEGLGIPGAFIIRNQHRNQIYLKSVTLEHVPGHGPVRFICDSWVYPTRRYDCDRVFFSNKAYLPSETPPPLIFYREQELNNLRGKGTGELKEWDRVYDYAYYNDLGSPEKGEDQA<br>RPVLGGSAYPYPRRARTGRPPNKKDPNTESRLFLLNLNIYVPRDEQFAQVKFSDFIGYAIKSVGQVLVPEIKAVFDKTFNEFDTFKDVLDLYETRTQVPDDKSLAKIKDCVPWELLREL<br>IRSDGERFLNFPVPQVIEADNTAWRTDEEFGREMLAGVNPVIIRILEEFPPRSKLDSQEYGNQDSSITREHIEKNMNGLTVEEAIEKKKLFILDHHDALMPYLRRINTTATKTYATRTLLL<br>LQDDGTLRPLAIELSLPHDDGDSCGAVNRVFTPCHGDGAEAMIWQLAKAYAAVNDSGYHQLISHWLNTHAVIEPFAIATNRQLSGLHPIYKLLKPHFRDTIHINALARHTLINAGGILE<br>TTVFPGRYALEMSSAVYKNWNFPDQALPRDLIKRGVAVPDTSQPHGLRLVIEDYPYAVDGLEIWSAIEHWVTEYCSLYYPTDDKVTGDAELQSWWKELREVGHGDLKEKPWWPK<br>METRLELIECCTIIIWVASALHAAVNFGQYAYAGYLPNRPTVSRRFMPEPGSPEYAELEKDQEAAFLKTITAQFQTLLGVSLIEMLSRHSSDEIYLGQRETPYWTGDEEPVRAFERFSAA<br>LVGIEDRIKEMNRNTELKNRTGPVKMDYMLLYPNTSDYSKKGGLIVKGIPNSVSI-
	CDS	ATGGGGATTTTCCCACCATGCGATTCTTCGGAGGTGATGGAGAAGCTGCTCGGATCCGTGTGCGGGAAAACCCGAGACGATCCCGCGACTGAAAGATCATCGAAGATCAAAG<br>GCTCCGTCGTCTTGATGAAGAAGAACGCGATGGACAGCACCGATTTTGGCGCGTCCTTGGTCGACCGGGTTTACGAGATCTTCGGTAGAGGCATCATCTTGCAGCTCGTCAGT<br>GCTCAACACTTCGACCCGGCTAATCCGAAGAGAGGGAAGCTAGGAAAGGAAGCTTACTTGGTGGATTGGATCACCAAGTTGACTTCACTTAAGGCCGAAGACGAGGCGTCGT<br>ACGACGTGGAATTCGACTGGGACGAGGGCCTCGGGATTCCCGGGGCTTTTATTATCCGGAATCAACACCGTAATCAAATCTACCTCAAGTCCGTCACACTGGAACATGTCCCG<br>GGACACGGTCCGGTCCGTTTTATATGCGACTCGTGGGTTTATCCTACGCGTCGCTACGACTGCGACCGTGTCTTTTTCTCAAACAAGGCGTACCTACCATCGGAAACGCCGCCA<br>CCTCTAATCTTTTATAGAGAGCAAGAGCTAAACAACCTTCGTGGAAAAGGCACCGGCGAGCTCAAGGAGTGGGACAGAGTGTACGACTATGCATACTACAACGACCTAGGAA<br>GCCCCGAAAAAGGCGAAGATCAAGCCCGCCCGGTGCTAGGTGGGTCGGCCTATCCATACCCTCGTAGGGCACGGACAGGCCGCCCACCAAACAAAAAAGATCCCAACACCG<br>AGAGCAGACTGTTTCTGCTAAACCTAAACATTTACGTACCACGAGACGAACAATTCGCCCAAGTGAAATTCTCCGATTTCATCGGGTACGCGATAAAGTCGGTAGGGCAAGTT<br>CTAGTCCCGGAGATCAAAGCCGTGTTCGACAAAACATTCAACGAATTCGACACGTTCAAGGATGTCTTGGACCTCTACGAGACGAGGACTCAGGTGCCCGATGATAAATCCT<br>TAGCCAAAATCAAAGACTGTGTTCCATGGGAGCTGCTTAGAGAGCTCATTCGCTCCGATGGTGAGAGGTTCCTCAACTTCCCAGTCCCTCAAGTTATAGAAGCGGATAACACT<br>GCGTGGAGAACGGATGAGGAGTTCGGACGCGAAATGTTGGCCGGAGTCAACCCTGTTATCATTCGAATTCTGGAGGAGTTTCCACCAAGAAGCAAACTAGACTCTCAAGAAT<br>ATGGCAATCAGGATAGTAGCATCACAAGAGAGCACATCGAGAAAAACATGAACGGCCTCACCGTCGAAGAGGCAATAGAGAAGAAGAAGCTGTTCATACTCGATCACCACG<br>ACGCGCTGATGCCGTACCTGCGGCGGATCAACACGACGGCGACGAAGACCTACGCCACCCGAACCCTCCTTCTCCTCCAAGACGACGGCACGTTGAGGCCACTCGCCATCGA<br>GCTCAGCTTGCCGCACGACGACGGGGACTCGTGCGGAGCCGTGAACAGAGTGTTCACTCCGTGCCACGGAGACGGCGCCGAGGCCATGATTTGGCAGCTGGCGAAAGCCTAC<br>GCCGCCGTCAACGATTCCGGCTACCACCAACTCATTAGCCACTGGTTGAACACGCACGCCGTGATAGAGCCGTTCGCGATAGCAACCAACAGGCAGCTGAGCGGGCTCCACC<br>CGATCTACAAGCTCCTGAAGCCGCACTTCCGCGACACGATCCACATCAACGCCTTGGCCCGCCACACGCTCATCAACGCCGGTGGAATACTCGAGACGACGGTCTTCCCGGG<br>AAGATACGCCTTGGAAATGTCCTCCGCCGTCTACAAGAACTGGAATTTCCCCGACCAGGCGCTCCCCCGGGACCTCATCAAAAGAGGGGTGGCGGTTCCCGATACGAGCCAG<br>CCGCACGGCCTTCGGCTCGTGATAGAAGACTATCCCTATGCAGTGGACGGCCTAGAAATATGGTCCGCGATCGAGCATTGGGTGACGGAGTACTGCTCTTTGTACTACCCGAC<br>CGACGATAAGGTGACCGGAGACGCGGAGCTTCAGTCCTGGTGGAAGGAGCTCCGCGAGGTCGGCCACGGCGACTTAAAGGAGAAGCCGTGGTGGCCGAAAATGGAGACCCG<br>GCTCGAGCTCATCGAGTGCTGCACCATAATCATATGGGTGGCGTCGGCCCTCCACGCCGCGGTGAACTTCGGCCAGTACGCCTATGCCGGTTATCTCCCGAACCGGCCCACTG<br>TGAGCCGGCGGTTCATGCCTGAACCGGGTAGCCCGGAGTACGCCGAGCTGGAGAAGGATCAAGAGGCTGCGTTTCTGAAAACGATCACGGCTCAGTTCCAAACGCTGCTTGG<br>GGTGTCGCTGATCGAGATGCTGTCGAGGCATTCGTCCGACGAGATTTATCTCGGACAGAGGGAGACGCCGTATTGGACCGGGGACGAAGAACCGGTTCGGGCGTTCGAGCGG

Gene	Sequence Type	Sequence (5'-3')
		TTCAGCGCGGCATTGGTTGGGATTGAGGATAGAATAAAGGAGATGAACCGGAACACGGAATTGAAGAACCGAACCGGGCCGGTTAAGATGGATTACATGCTGCTGTATCCC<br>AACACTTCGGATTATTCCAAAAAAGGAGGACTTATTGTGAAAGGAATTCCAAATAGTGTATCTATTTGA
SmLOX3	Protein	MSCNCSEMWENLLGCLCGEIKDSPKIEQQQQKKKIKGIVVLKKKNVMDVTDVGASLLDRIHELFGKGVSLQLVSSHSAEPGNSRKGKVGKEAYLEDWLPKLTSLNAEDHASFNVT<br>FDWDVESLGFPGALIVKNQHHSQLYLKSVALENVCGYGRVDFVCDSWVYPVRRYKYDRVFFASNMYLPGETPEPLLFYREQELRSLRGDDRLEGSGILKEWDRVYDYAYYNDLGA<br>PEKGEEYARPVLGGPDYPYPRRARTGRPPNKKDPTNESRLFLLSLNTYVPSDERFSQVKFSDFIGYALKSLGQVIVPEIKALFDKTINEFDTFEDVLRLYEGGVRVPDDGHSLSKLRKC<br>VPWELIRELLRSDGERFLKFPVPDVIKVDKTAWRTDEEFGREMLAGVNPVVIRRLQEFPPKSRLDRRQYGDQDSRITRAHLEENMNGLAIEEAIQRNKMFILDHHDALMPYLRRINTT<br>STKTYASRTILLLRDDGTLRPLAIELSLPHEDGDALGATSRVFAPCEHGVGNAIWQLAKAYVAVNDSGYHQLISHWLNTHAVMEPFVIATNRQLSSIHPVHGLLKPHFRDTIHINALAR<br>QILINAGGVLETTVFPARYAMEMSSFVYRDWNFTEQALPVDLIKRGVAVADESQQPHGLKLLIPDYPFAVDGLEIWSAIQAWVTEYCCIYYPTDDAVSGDAELQSWWKELREVGHG<br>DLKEKPWWPRMETRRELIESCTIIIWVASALHAAVNFGQYPYAGYLPNRPTVSRRFMPEPGSAEFAEMEREPELAYLKTITAQLQTLLGISLIEILSRHSTDEVYLGQRDGGWSGEEAA<br>AAFERFGAALVDVERRIRERNGNRALKNRHGPVEVQYTLLYPNTSDYSREGGLTGKGIPNSISI-
SmLOX3	CDS	ATGTCGTGTAATTGCTCAGAGATGTGGGAGAATTTACTGGGCTGCTTGTGCGGAGAGATAAAAGATTCGCCCAAAATCGAGCAGCAGCAGCAGCAGAAGAAGAAGATCAAA<br>GGCATTGTCGTGTTGAAGAAGAAGAATGTGATGGACGTCACCGACGTCGGCGCCTCCTTGCTCGACCGCATCCACGAGCTCTTCGGCAAAGGCGTTTCTCTGCAGCTCGTCAG<br>CTCTCATTCCGCTGAGCCAGGAAACTCGAGAAAGGGGAAGGTAGGGAAAGAGGCATATTTGGAGGATTGGTTGCCGAAGCTGACTTCTCTCAATGCAGAGGATCACGCCTCT<br>TTTAATGTCACCTTTGATTGGGATGTTGAATCTCTCGGATTTCCCGGCGCTTTGATCGTCAAAAACCAACACCATAGCCAGCTATACCTCAAGTCCGTCGCCTTGGAAAACGTC<br>TGCGGTTACGGCCGCGTCGATTTCGTCTGCGACTCGTGGGTTTACCCCGTGCGTCGCTATAAATACGACCGCGTCTTTTTCGCGAGCAACATGTACCTCCCGGGGGAGACGCC<br>GGAGCCCCTGCTCTTTTACAGAGAGCAGGAGCTCCGGAGCCTTCGAGGCGATGACCGCCTCGAAGGCTCTGGGATCCTCAAGGAGTGGGATAGAGTGTATGATTATGCGTAC<br>TATAACGACCTAGGCGCCCCCGAGAAGGGCGAGGAATATGCTCGCCCCGTGCTCGGGGGCCCCGACTATCCGTACCCCCGTCGAGCGAGGACGGGGCGTCCACCGAATAAA<br>AAAGATCCCACCAATGAGAGCAGGCTGTTTCTGTTGAGCTTGAACACGTACGTGCCGAGTGATGAGAGGTTCAGTCAGGTGAAATTCTCGGATTTCATAGGTTATGCTCTGAA<br>GTCTCTGGGGCAGGTGATAGTGCCGGAGATCAAGGCGTTGTTCGACAAGACTATAAACGAGTTCGACACGTTTGAAGACGTTCTCCGCCTCTACGAAGGCGGAGTTAGGGTC<br>CCGGATGACGGCCACTCCCTGAGTAAACTCAGGAAGTGCGTCCCGTGGGAGTTGATCAGGGAACTCCTTCGTTCCGATGGCGAACGCTTTCTTAAGTTCCCCGTGCCCGATGT<br>TATTAAAGTGGACAAGACTGCATGGAGAACAGATGAAGAATTTGGAAGGGAAATGCTAGCAGGAGTCAACCCTGTTGTCATTCGACGTCTCCAGGAGTTTCCACCGAAAAGC<br>AGACTAGATCGGCGACAATACGGCGATCAAGATAGCCGCATCACGCGAGCGCACCTCGAAGAAAACATGAATGGACTCGCCATAGAAGAAGCCATACAGAGGAACAAGAT<br>GTTCATACTAGATCACCACGACGCATTGATGCCGTACCTGAGAAGGATCAACACAACATCAACAAAGACCTACGCGAGCCGAACGATCCTCCTCCTCCGAGACGACGGCACT<br>CTGAGGCCACTCGCCATCGAGCTCAGCTTGCCGCACGAGGACGGGGACGCCCTCGGGGCCACGAGCCGGGTGTTCGCCCCGTGCGAGCACGGGGTGGGGAACGCCATCTGGC<br>AGCTGGCGAAGGCCTACGTGGCGGTCAACGACTCGGGCTATCACCAGCTCATAAGCCACTGGCTCAACACTCACGCGGTGATGGAGCCGTTCGTGATCGCGACGAACAGGCA<br>GCTGAGCTCGATCCACCCCGTGCACGGGCTTCTGAAGCCGCACTTCCGCGACACGATCCACATCAATGCCCTGGCTCGGCAGATCCTCATCAACGCCGGCGGGGTGCTCGAGA<br>CCACCGTGTTCCCGGCGAGATACGCCATGGAAATGTCCAGCTTCGTTTATAGAGACTGGAACTTCACTGAGCAAGCACTCCCCGTAGATCTCATCAAAAGGGGAGTGGCGGTT<br>GCCGATGAAAGCCAGCAGCCCCACGGCCTCAAACTCCTAATACCAGACTACCCTTTCGCCGTCGACGGCTTGGAAATCTGGTCCGCAATCCAAGCCTGGGTCACCGAATACTG<br>CTGCATCTACTACCCCACCGACGACGCCGTTTCCGGCGACGCCGAGCTCCAATCATGGTGGAAGGAGCTCCGCGAGGTCGGCCACGGCGACTTAAAGGAGAAGCCGTGGTGG<br>CCGAGAATGGAGACGCGGCGCGAGCTGATCGAGTCCTGCACGATCATCATATGGGTGGCGTCGGCCCTCCACGCCGCCGTCAACTTCGGGCAGTACCCTTACGCCGGGTATC<br>TCCCGAACCGCCCCACCGTGAGCCGCCGGTTCATGCCCGAGCCGGGCAGCGCCGAGTTCGCCGAGATGGAGAGGGAGCCCGAGCTGGCCTACCTGAAGACCATCACTGCGCA<br>GCTGCAGACGCTGCTCGGGATCTCTCTGATCGAGATACTGTCGAGGCACTCGACGGACGAGGTGTATCTCGGTCAGAGAGACGGCGGGTGGAGCGGCGAGGAGGCGGCGGC<br>GGCGTTTGAGAGATTTGGGGCGGCGCTGGTTGATGTGGAGAGGAGGATAAGGGAGAGGAATGGGAACAGGGCGTTGAAGAACCGGCATGGGCCGGTGGAGGTGCAGTACA<br>CCTTGCTGTACCCGAACACCTCTGATTATAGTAGAGAGGGAGGCCTTACTGGGAAGGGAATTCCCAACAGCATCTCTATATGA
SmLOX4	Protein	MDERIVNGDVGGQVRGVVVLMKKNVLGVNNVAASVVDRVGEFLGRKVALQLIGTAAHPDSAAESCRGKLGKKAYLEDWITKITPMTAAATYNVTFEWSKEMGYPGAFTITNFHH<br>SEFYLKTLTLEDVPGHGRVHFICNSWVYHAECYKKDRIFFANQTYLPSQTPPPLRGYREEELENLRGTGTGKLEEWDRVYDYDIYNDLGDPDKGPEQNVPFLGVQLNTHILAEEELA<br>GLWLHQHLRINHMCLITCYFVDPRTEGRIPAYNSLSIYVPRDERFSHLKMSDFVAYALKSLFQFLVPVLKDLFDKTPDEFDSFEDVLRLYDGATEVSNVTMLEKIRQHIPLETIRELLRS<br>DGEKTYNFPLPHVIKEDKDAWRSDEEFGREMLAGINPVIIQCLQAIRNNKLYILDHHDSLMPYLRRINTTAAKTYATRTILFLKEDGTLRPIAIELSLPHPEGDHYGAVSRVCTPAEDGV<br>EGTIWLMAKAYVAVDDSGYHQLISHWLNTHASIEPFIIATNRQLSVLHPIHKLLQPHFRDTMNINALARQALICAGGFVEMTVFPGKYSLEMSSMVYKDWVFPEQALPGDLIKRGVAI<br>KDSSCPHGVRLLIEDYPFAVDGLEIWGAIKTWVQDYCNLYYKSDEMVQEDPELQAWWTEIREKGHGDKKDEPWWPTMQSCEELIESCTTIIWVASALHAALNFGQYPYGGYLPNRP

Gene	Sequence Type	Sequence (5'-3')
		ATSRRFIPDIGTPAYEEMKLNPEKAYLKTITPQMQGVLGISLVEILSRHSSDEIYLGQRDSPEWTADTEARKAFERFGDRLRETEQKFIDLNNDKRWRNRSGPVKMPYTLLYPSSEIGLT<br>GRGIPNSISM-
	CDS	ATGGATGAGCGCATCGTCAATGGCGACGTCGGCGGCCAGGTGAGGGGAGTGGTGGTGCTGATGAAGAAGAACGTGCTCGGAGTGAACAACGTCGCGGCGTCGGTCGTCGAT<br>CGAGTCGGCGAGTTTTTGGGGAGGAAGGTCGCGCTGCAGCTCATCGGCACCGCCGCTCATCCAGATTCTGCTGCAGAAAGTTGCAGAGGCAAACTTGGGAAGAAAGCATATC<br>TAGAAGACTGGATCACAAAAATCACTCCTATGACAGCAGCTGCGACATACAATGTCACCTTCGAGTGGAGCAAGGAGATGGGCTATCCGGGGGCATTCACAATCACAAATTT<br>TCACCATAGTGAATTCTACCTCAAGACGCTGACTCTTGAAGACGTCCCTGGCCATGGCCGCGTCCATTTCATCTGCAATTCTTGGGTTTACCATGCTGAATGCTACAAAAAGGA<br>CCGGATATTTTTCGCTAATCAGACATATCTCCCTAGCCAAACGCCTCCGCC<br>ACTTCGTGGTTACAGAGAAGAAGAATTGGAGAACCTGAGAGGAACTGGAACAGGAAAACTTGAGGAATGGGATAGGGTGTACGACTATGATATATACAATGATTTAGGTGA<br>TCCAGATAAAGGTCCAGAGCAGAACGTCCCATTCTTGGGGGTTCAACTGAATACCCATATCCTCGCAGAGGAAGAACTGGCCGGCCTCTGGCTTCATCAGCATCTACGTATAA<br>ACCACATGTGTTTAATCACTTGCTACTTTGTAGATCCCAGAACCGAGGGCAGGATACCAGCATATAACAGCCTGAGCATCTACGTCCCGAGGGATGAAAGGTTTAGTCATCTG<br>AAGATGTCAGATTTTGTAGCATATGCACTCAAGTCACTATTTCAGTTCTTGGTGCCAGTGCTTAAGGATCTGTTTGACAAAACTCCCGATGAGTTTGACTCATTTGAGGACGTG<br>CTACGGCTCTATGATGGTGCAACCGAAGTATCTAATGTAACTATGTTGGAGAAAATTAGACAACATATACCCTTAGAGACGATAAGGGAGCTACTCAGATCAGATGGAGAGA<br>AAACATACAATTTTCCGCTGCCACATGTCATCAAAGAAGATAAAGATGCCTGGCGATCTGATGAAGAATTCGGTAGAGAAATGCTGGCTGGAATCAATCCTGTCATTATCCA<br>ATGCCTCCAAGCAATCAGGAACAATAAGCTGTACATACTAGATCACCACGACAGCCTGATGCCATACCTGAGGAGGATAAATACTACAGCTGCAAAGACTTATGCAACGAGA<br>ACAATTCTCTTCTTGAAAGAAGATGGAACACTGAGGCCCATAGCAATTGAATTAAGTTTGCCACATCCAGAAGGGGATCATTACGGTGCAGTAAGTCGCGTATGCACCCCAG<br>CAGAAGATGGAGTTGAAGGTACCATCTGGCTGATGGCGAAGGCTTATGTGGCAGTAGATGACTCTGGCTATCACCAGCTCATAAGTCACTGGTTAAACACTCATGCATCAATT<br>GAGCCATTTATTATCGCCACCAACAGACAGCTGAGTGTACTCCACCCAATTCATAAACTTTTGCAGCCTCACTTCAGAGATACAATGAATATAAATGCACTAGCTCGTCAAGC<br>CCTTATATGTGCTGGAGGGTTTGTTGAGATGACAGTATTTCCAGGAAAATATTCTTTGGAAATGTCATCTATGGTTTATAAGGACTGGGTTTTCCCAGAGCAAGCACTTCCAGG<br>AGACCTTATAAAAAGAGGGGTGGCAATCAAAGACTCTAGCTGCCCACATGGAGTGCGATTACTGATAGAGGACTATCCTTTTGCTGTTGATGGACTCGAGATCTGGGGAGC<br>AATCAAAACATGGGTTCAGGATTACTGCAATCTTTACTACAAGAGTGATGAAATGGTTCAGGAAGATCCCGAGCTTCAGGCGTGGTGGACTGAAATACGAGAAAAGGGACAT<br>GGAGATAAGAAAGACGAACCCTGGTGGCCCACAATGCAATCATGTGAAGAGCTGATAGAATCCTGCACCACTATCATATGGGTGGCGTCTGCCCTTCACGCAGCATTAAATT<br>TCGGACAGTACCCTTATGGAGGGTAC<br>CTGCCAAATCGTCCGGCTACTAGCCGGAGATTCATTCCAGATATTGGCACTCCTGCCTATGAAGAAATGAAGTTGAATCCTGAAAAGGCTTACTTGAAAACCATCACTCCCCA<br>GATGCAAGGTGTGCTTGGCATCTCCCTCGTAGAAATTTTATCCAGGCATTCTTCCGATGAGATTTATCTTGGACAGCGAGACTCTCCTGAATGGACAGCAGACACAGAGGCAC<br>GCAAAGCATTCGAAAGGTTTGGGGACCGGTTAAGAGAGACAGAGCAAAAGTTCATTGATTTGAACAATGATAAGAGATGGAGGAATAGGTCGGGGCCAGTGAAGATGCCGT<br>ACACTTTGCTATACCCCAGCAGTGAGATCGGACTGACAGGCAGGGGAATTCCCAACAGCATCTCAATGTAA
SmLOX5	Protein	MPLKTLESTTHEYSKMKKRHERRATRRGLCSSLARCLSLCQGDVAARKVRSFGTPGKNIVYGEIIIQVGHGESGPGKAASVWLYSDSNVDPGTGKGKLTQKAALQKGSIDEETDSSS<br>RTKVLRYEVEFNVEPDFGAPGAFVMKNEHKHEFFLQNVSLQGDNNHIVHFECNSWIYPFHLTKQDRVFFPNTCYLPNQTPPSLMELRKDELARLRGNGTGERRKWEQIYDYDCYND<br>LGIPERSLGYGRPVLGGTQSFPYPRRLRTGRPSNNYDTRAERRLRGWFNTDIYVPPDERFSQQKLSEFTTNAVRAAVHFVMPEAGAIFNQETSHFESFDQIHRLFMVIKEKPVKFQLPRI<br>AAGNQFAWMDDDEFGRQMLAGTNPTVISSLQNFPPDGSNGVKSSIKGSDIEHNLDGLTVAEAMNQWRLFKLDHHDYLMPFLTRINRDEGVCAYASRTLLFLRDDCTLKPVAIELSLP<br>GSLSHEEISRVFRPAHDGTEAALWQLAKAHVAAADSVYHHLVSHWLNAHAVVEPFIIATRRKLSVMHPIHRLLEPHFKDTMQVNALARSMLLNAGGIFEKILFTGDISMELSSSLYRS<br>WRFDEQSLPADLIKRGFARYNPNQPENIELLFQDYPYGADGLDIWIAIKNWVIEYCSYFYENNASLRSDNEIADWWWEIRNVGHGDKSNETWWYEMTTVAALIETITTLIWTVSAHH<br>AAISLGQYDYAGYPPNRPTACRKPIPYEGTMEFAELLKDPDKYFLSMLPEKFETALGIAMVDVLSRRLSDEMSLGQQPSLKWIADGRVLRMFRSFEDDLEEVKQRIQARNREPKLKN<br>RRGRADFRYELLCPETSEVRSSDGILRSGIPNSISI-
	CDS	ATGCCTTTAAAAACTTTGGAGTCCACAACCCATGAGTATAGCAAGATGAAGAAAAGGCACGAACGGAGAGCAACACGGCGAGGCTTGTGTTCAAGCCTCGCCCGGTGCCTGA<br>GCCTGTGCCAAGGTGACGTGGCCGCCAGAAAAGTTCGGAGTTTCGGTACTCCCGGGAAGAACATCGTATACGGAGAGATCATCATCCAGGTAGGCCATGGAGAGTCAGGCCC<br>CGGAAAAGCAGCCTCAGTTTGGTTGTACAGCGATTCCAACGTCGATCCCGGCACTGGAAAAGGGAAACTTACGCAGAAAGCCGCTCTCCAAAAAGGAAGCATCGACGAGGA<br>AACAGACAGCAGCTCCAGAACAAAAGTTCTGAGGTATGAAGTAGAGTTCAACGTTGAGCCAGACTTCGGGGCTCCAGGAGCATTCGTCATGAAAAACGAGCACAAACACGA<br>GTTTTTCCTTCAAAATGTGTCTCTGCAAGGAGACAACAACCACATAGTTCATTTCGAATGCAACTCGTGGATCTATCCTTTCCACTTAACCAAACAAGATCGTGTTTTCTTCCC<br>AAACACGTGTTATCTCCCAAACCAAACACCACCTTCTCTGATGGAACTGCGAAAAGATGAACTCGCGAGATTGAGAGGGAATGGAACTGGAGAGAGACGAAAATGGGAACA

Gene	Sequence Type	Sequence (5'-3')
		AATCTACGACTACGACTGCTACAACGATCTTGGCATACCTGAAAGAAGCCTTGGATATGGAAGACCTGTTTTAGGTGGAACACAGTCGTTTCCATATCCGCGTAGGTTGAGAA<br>CCGGTCGCCCTTCAAACAACTACGATACTCGAGCTGAGAGGAGACTACGTGGATGGTTCAACACAGATATATATGTACCTCCAGATGAACGTTTCAGCCAACAGAAACTCTC<br>GGAATTCACAACAAACGCGGTCAGGGCTGCTGTCCATTTTGTGATGCCTGAAGCAGGAGCAATCTTCAACCAAGAAACGAGCCACTTTGAGTCCTTCGATCAGATTCATAGGC<br>TGTTCATGGTGATCAAGGAGAAACCTGTTAAGTTCCAATTGCCTCGGATTGCAGCAGGGAACCAATTTGCATGGATGGATGACGATGAATTTGGACGGCAGATGCTTGCTGG<br>AACTAATCCAACTGTAATTTCATCTCTGCAGAATTTCCCACCAGACGGCAGCAATGGAGTCAAGAGTTCGATAAAGGGATCCGACATAGAGCATAACCTTGATGGTCTGACA<br>GTTGCAGAGGCAATGAACCAGTGGAGACTTTTCAAGCTAGATCACCACGATTATCTGATGCCATTCTTGACGAGAATAAACAGAGATGAGGGTGTATGTGCTTATGCATCGC<br>GCACGCTACTCTTCTTGAGAGACGATTGCACGCTAAAACCAGTAGCCATAGAACTGAGCTTGCCTGGCTCTTTGTCCCACGAGGAAATTAGCAGAGTTTTCCGTCCAGCACAT<br>GACGGGACAGAAGCTGCTCTCTGGCAACTAGCAAAAGCTCATGTAGCAGCAGCAGATTCAGTTTACCATCATCTAGTCAGCCATTGGCTAAATGCACATGCTGTTGTTGAGCC<br>ATTTATCATTGCAACTCGAAGGAAGTTGAGTGTTATGCACCCCATTCACCGGTTACTCGAACCTCATTTCAAAGACACCATGCAAGTAAATGCACTGGCAAGGAGCATGTTGC<br>TAAATGCTGGAGGAATTTTTGAAAAGATACTGTTTACCGGTGACATCTCAATGGAGCTCTCGTCGTCTCTGTACAGAAGCTGGAGATTCGATGAGCAAAGCCTCCCTGCAGAC<br>CTGATCAAAAGAGGCTTTGCCAGATATAACCCGAATCAGCCAGAAAACATCGAGCTTCTCTTTCAAGATTATCCCTATGGTGCAGATGGACTTGATATCTGGATAGCCATAAA<br>GAACTGGGTGATAGAATACTGTTCTTACTTCTATGAAAATAACGCCTCTTTAAGGTCTGACAATGAGATTGCAGACTGGTGGTGGGAGATCAGAAATGTCGGTCATGGCGATA<br>AGAGCAACGAAACATGGTGGTATGAAATGACAACTGTGGCTGCACTGATTGAGACTATAACAACTCTAATATGGACCGTCTCAGCTCATCATGCTGCAATAAGCTTAGGACA<br>GTACGACTACGCAGGCTATCCACCAAACCGTCCAACAGCATGTCGGAAACCTATTCCATACGAAGGGACAATGGAGTTTGCCGAGCTTCTCAAAGATCCAGACAAATATTTT<br>CTCAGTATGTTACCTGAAAAGTTTGAGACTGCTCTTGGAATAGCTATGGTTGATGTTCTGTCAAGGCGCTTGTCCGATGAAATGAGCTTAGGACAACAGCCTTCGCTGAAATG<br>GATAGCAGATGGAAGGGTACTTCGAATGTTCAGAAGTTTTGAAGATGACCTTGAAGAAGTGAAGCAAAGAATACAAGCTAGAAACCGAGAACCAAAACTGAAGAACAGACG<br>GGGAAGAGCTGATTTCCGGTACGAGCTTCTGTGTCCTGAAACATCAGAGGTTAGATCAAGTGATGGAATCCTGAGAAGCGGAATTCCCAACAGCATATCCATATGA
SmLOX6	Protein	MLRAQASPTTLRRPEAFPAAVKQHRASSLPGLRPGRRAAIRAVISSGDSKAGVETAEKAVESSSGALETFSTSGGVSVRAVIRIRKKMKEKLLDKIEDQWDSFLNGIGRGISLQLISQEP<br>HLGKSGETFVRGWLPRLSDNPYVVEYGADMVVSKDFGQPGAVVVTNFHEKEVFLMEIVVHGFKEGPLFFTADTWIHSRKDNPESRIIFKNQAYLPSETPAGIKSLRQQDLLSVRGDG<br>KGERKLHERIYDYDVYNDLGNPDKGEELARPVVGGEDRPYPRRCRTGRRPTLTDPDAESRIEKPDPVYVPRDETFEEVKQNTFSAGRLKAVLHNLIPLIVSTLASSDVPFTNFSDIDNL<br>YGDILLQHEPKEAKKNQLLANLMNQVLNVGDKLLKYEIPAIIRRDRFSWLRDNEFARQTLAGVNPVNIELLKELPVTSKLDPAIYGPPESAITREIIARELNGLSVEEAIENKKLFIIDYH<br>DLLLPFIEKMNELPDRKAYASRTVYYFDDGILRPIVIELSLPPTPSSPRNKKIYTHGHGATSSWIWKLAKAHVCSNDAGVHQLMNHWLKTHACTEPYIIATHRQLSSMHPIYKLLHPH<br>MRYTMEINALARQSLINGGGVIEACFSPGKYAMEISSAAYEKLWRFDMEALPADLLHRGMAVEDPTVPGGVKLVIEDYPYAADALLIWAAIQELVESYVEHYYSEPNSISSDVELQA<br>WWNEIKNVGHYDKRNETWWPNLNTQDDLSSILTTMIWTASGQHAAINFGQYPFGGYPPNRPTLMRKLIPQLGDPEYEKFLHNPEYTFLTSLPTQLQATKVMAVQDTLSTHSPDEEY<br>LHQLDQIHRLSSNDPQVQKFFENFSVQLEEIERIINLRNKNVELTNRTGAGVPPYELLLPTSPPGVTGRGIPNSISI-
	CDS	ATGCTTAGAGCTCAGGCCAGCCCCACCACTCTCCGGCGGCCGGAGGCCTTCCCCGCCGCCGTGAAACAGCACAGGGCCTCCAGCCTGCCCGGGCTCCGGCCCGGGAGGCGGG<br>CGGCAATAAGGGCTGTGATCAGCAGTGGAGATAGCAAAGCAGGAGTGGAAACAGCAGAGAAGGCTGTGGAGAGTAGTAGTGGAGCTTTGGAGACTTTTTCAACAAGTGGAG<br>GTGTGAGTGTGAGAGCAGTGATCAGAATAAGGAAGAAGATGAAGGAGAAGTTGCTTGACAAGATTGAGGATCAGTGGGATTCTTTCCTCAATGGGATTGGGAGAGGGATCT<br>CACTTCAGCTCATCAGCCAAGAACCTCATCTTGGCAAGAGTGGTGAGACTTTTGTGAGGGGGTGGTTGCCAAGATTATCAGACAATCCTTATGTGGTGGAGTATGGAGCTGAC<br>ATGGTGGTGTCAAAGGATTTTGGGCAGCCAGGAGCTGTAGTTGTGACCAATTTCCACGAGAAAGAGGTGTTCTTGATGGAGATTGTGGTTCATGGCTTCAAAGAAGGACCCTT<br>ATTCTTCACGGCTGATACATGGATTCATTCCAGGAAAGACAATCCTGAAAGCAGAATAATATTCAAGAATCAGGCTTACCTGCCGTCTGAGACCCCAGCTGGGATCAAGAGT<br>CTCCGGCAGCAGGACCTGTTGAGCGTTCGTGGCGATGGGAAAGGAGAGAGGAAGCTGCACGAGAGGATATACGATTATGATGTCTACAATGATTTAGGTAATCCTGATAAGG<br>GCGAGGAGCTAGCCAGGCCGGTTGTGGGCGGTGAAGACAGGCCTTATCCTAGGCGTTGTCGGACTGGTAGACGTCCCACTTTGACAGATCCCGATGCAGAGAGTAGAATCGA<br>GAAGCCTGATCCCGTGTATGTTCCTCGTGATGAGACGTTCGAGGAGGTTAAACAGAACACCTTCTCAGCTGGAAGGCTCAAGGCTGTGCTCCACAACCTGATACCATTGATCG<br>TTTCCACATTGGCTAGTTCGGACGTCCCGTTCACAAACTTCTCCGACATAGATAATCTTTATGGTGATATTCTTCTGCAACATGAGCCAAAGGAAGCTAAAAAGAATCAGCTC<br>CTTGCCAATCTCATGAACCAAGTATTGAATGTTGGTGATAAGTTGCTCAAATACGAAATACCAGCCATTATTAGGCGTGACAGGTTTTCATGGCTAAGGGATAACGAGTTTGC<br>ACGTCAGACTTTGGCCGGGGTAAATCCTGTTAACATCGAGCTACTAAAGGAGCTTCCCGTTACAAGCAAACTAGATCCTGCAATATATGGCCCTCCAGAATCTGCAATCACAA<br>GGGAAATAATAGCCAGAGAGCTTAACGGACTCAGCGTTGAAGAGGCTATAGAAAACAAGAAGCTGTTTATAATTGACTATCATGACTTGCTCTTACCGTTCATTGAGAAGAT<br>GAATGAGTTGCCTGATAGAAAAGCTTACGCTTCCAGAACTGTCTACTATTTTGATGATGGCATTCTTAGGCCGATTGTTATCGAGCTATCACTTCCTCCAACTCCTTCATCTCC<br>AAGGAACAAGAAAATTTATACCCATGGGCACGGTGCTACCTCTAGCTGGATCTGGAAGCTGGCTAAAGCTCATGTTTGCTCCAATGATGCAGGCGTTCACCAACTCATGAACC

Gene	Sequence Type	Sequence (5'-3')
		ACTGGTTGAAGACTCATGCCTGCACGGAACCTTATATAATTGCCACTCATAGGCAGCTAAGCTCAATGCATCCAATCTACAAACTACTACACCCCCACATGCGGTACACCATG<br>GAAATCAATGCCCTTGCAAGGCAAAGTTTGATAAACGGTGGTGGAGTCATAGAGGCTTGTTTCAGCCCCGGAAAGTATGCCATGGAGATAAGCTCTGCTGCCTATGAGAAAT<br>TGTGGCGGTTTGACATGGAAGCATTACCAGCAGATTTACTTCACAGGGGAATGGCTGTTGAGGATCCCACGGTGCCCGGTGGTGTCAAACTTGTAATAGAAGATTATCCTTAT<br>GCTGCAGACGCACTTCTCATATGGGCTGCCATACAGGAATTGGTAGAATCTTACGTTGAGCATTATTATTCGGAACCCAATTCCATCTCATCTGATGTGGAGCTTCAGGCCTGG<br>TGGAACGAGATAAAGAACGTGGGACATTATGACAAGAGAAACGAAACGTGGTGGCCAAACCTCAATACACAAGATGACTTATCAAGCATACTTACAACCATGATTTGGACT<br>GCTTCTGGTCAGCATGCTGCTATCAACTTCGGACAGTATCCGTTTGGGGGATATCCACCTAACCGTCCGACTCTCATGAGGAAATTAATCCCTCAACTGGGTGACCCTGAGTA<br>CGAGAAGTTCCTTCACAACCCCGAGTACACTTTCCTGACCTCGTTGCCCACGCAACTCCAGGCTACAAAGGTTATGGCTGTCCAAGACACCCTATCAACACATTCTCCAGATG<br>AAGAGTACCTCCATCAGTTGGACCAGATTCACAGGCTTTCCTCTAATGATCCTCAAGTTCAAAAGTTTTTCGAAAATTTTTCTGTGCAATTAGAAGAGATAGAACGAATCATC<br>AATCTAAGGAATAAGAATGTAGAACTGACAAACAGAACTGGTGCTGGTGTTCCTCCATATGAATTGCTTCTACCCACTTCACCTCCTGGCGTTACTGGTCGTGGTATTCCCAA<br>CAGCATTTCCATCTGA
SmLOX7	Protein	MTPDEGDRLAWFRDAEFARQTLAGINPCAIQLVTEWPLKSKLDPEVYGPAESAITKELLEQDIRGFMTAVKQKKLFILDYHDLLLPYVQKVRELDGTTLYASRTLFFLMPAGTLRPIAI<br>ELTRPQTEDKPQWRQVFKPSWDPTGIWLWRLAKTHILAHDSGYHQLISHWLRTHCCTEPYIISTHRQLSAMHPIHRLLHPHLRYTMEINALARQALINANGIIETSFSPKKYSIELSSAA<br>YDQLWQFDLQALPAELISRGMAVEDPSAPHGLKLTIEDYPYANDGLLIWDSIKQWITDYVSHFYPKANLVQSDSELQAWWTEIRTVGHGDKKDEPWWPELNTQEDLIGILTTIIWTA<br>AGHHAAVNFGQFDFGAYFPNRPTIARTPMPCEEPSPAVEEKFMERPEAILLDCFPSQLQATVIMTILDVLSNHSPDEEYIGGRIEPYWEDDKVISAAFERYQGRLKEIEGVIDARNADTN<br>LKNRSGAGVVPYELLKPYSGPGVTGKGVPNSISI-
	CDS	ATGACACCCGACGAAGGAGATAGGCTTGCATGGTTTAGGGATGCGGAGTTCGCTCGACAAACGCTAGCAGGCATCAATCCATGTGCCATACAATTGGTCACGGAATGGCCGT<br>TGAAGAGTAAACTCGATCCCGAGGTGTATGGACCGGCGGAGTCAGCAATCACGAAGGAACTCCTAGAACAAGATATCAGAGGATTCATGACAGCAGTGAAACAGAAGAAGC<br>TTTTCATCCTAGACTATCACGATCTGCTCTTGCCTTATGTGCAAAAGGTAAGAGAGCTGGACGGAACAACTCTTTATGCATCGAGGACGTTGTTCTTCTTGATGCCAGCGGGCA<br>CGTTAAGGCCCATTGCCATCGAGCTAACTCGACCACAAACTGAAGACAAGCCCCAGTGGAGGCAAGTTTTTAAACCATCTTGGGATCCCACCGGCATCTGGTTGTGGAGACTC<br>GCTAAAACTCACATCCTTGCTCACGACTCCGGTTATCATCAGTTGATCAGTCATTGGCTACGAACTCATTGTTGCACCGAGCCTTACATAATTTCCACACACAGGCAACTAAGT<br>GCAATGCACCCAATTCATCGACTGCTGCATCCTCATCTGCGATACACAATGGAGATTAATGCATTGGCTCGTCAGGCACTCATAAATGCCAATGGGATCATTGAGACTTCTTTT<br>TCTCCTAAAAAGTACTCCATTGAGTTGAGCTCAGCTGCCTATGACCAGCTGTGGCAATTTGACTTACAGGCACTACCGGCAGAATTAATTAGCAGAGGAATGGCTGTGGAAGA<br>TCCAAGTGCGCCTCACGGCCTGAAGCTGACTATTGAGGACTACCCTTATGCAAACGACGGTTTGCTGATATGGGATTCCATCAAACAGTGGATCACTGACTACGTCTCACACT<br>TCTATCCTAAAGCCAACTTGGTGCAGTCGGATTCCGAGCTTCAGGCGTGGTGGACAGAAATAAGAACAGTAGGGCACGGTGATAAGAAGGACGAGCCATGGTGGCCGGAGC<br>TCAACACTCAAGAGGATCTAATCGGAATTTTAACAACAATTATATGGACAGCTGCAGGTCACCACGCAGCTGTAAACTTTGGGCAATTTGATTTTGGAGCATACTTTCCGAAT<br>AGGCCAACCATTGCTCGAACGCCGATGCCTTGCGAAGAACCGAGCCCCGCTGTTGAAGAGAAGTTTATGGAAAGGCCGGAGGCGATCCTTCTAGACTGTTTCCCATCGCAAC<br>TTCAAGCAACAGTAATTATGACGATTTTAGATGTTCTGTCCAACCACTCTCCTGATGAAGAGTACATTGGTGGTCGAATTGAGCCGTACTGGGAGGATGATAAAGTGATCAGC<br>GCTGCATTTGAACGTTACCAAGGGAGATTGAAGGAGATTGAAGGGGTCATTGATGCCAGAAATGCTGATACCAACCTCAAGAATAGGTCGGGAGCTGGAGTGGTGCCGTACG<br>AGCTTTTGAAACCCTATTCTGGACCTGGTGTCACAGGGAAGGGTGTCCCAAATAGCATCTCTATTTAG
SmLOX8	Protein	LSKRSSMALARELMGSSLMEKSSFLAASSSSTRLVFSQKRNRSFLPLEGRRVQVVIRRNTTPVAAISEDLDLVKVLPEKAVKFKVRAVVTVRNKNKEDFKETLVKHLDAFTDKIGRNV<br>VLELISTGFDPKTKAPKKSKEAVLKDWSKKANLKTEKVNYTAEFEVDSNFGIPGAITVANKHQKEFFLESITIEGFACGPVHFPCNSWVQPKKDHPGKRIFFTNKPYLPHETPEGLKAL<br>REKELRELRGDGQGERKLSDRVYDFDVYNDLGNPDKGIDFARPILGGEKIPYPRRCRTGRSPTDTDMNSESRVEKPLPMYVPRDEQFEESKMNAFSTGRLKGVLHNLIPSLMASISAN<br>NKDFKGFSDIDSLYSEGLLLKLGLHDEIMNKVPLPKSVSKFQEGGLLKYDLPKIVSKDKYAWLRDDEFARQAIAGVNPVTIERLQSFPPVSKLDPEIYGPQESALKEEHIAGQLNGMTI<br>QEALDANKLFIVDYHDVYLPFIDKINALDGRKSYATRTVYFLTDAGTLKPIAIELSLAPSSPRPKRVVTPPVDATTYWMWQLAKAHVCSNDAGVHQLVNHWLRTHAALEPFILAAHR<br>QMSAMHPIFKLLDPHMRYTLEIKRWLVRA-
	CDS	TTGTCGAAGAGATCGAGCATGGCGCTAGCTAGAGAGCTCATGGGCTCGTCTCTAATGGAGAAATCCTCATTTCTCGCCGCTTCATCGTCGTCAACGAGGCTGGTTTTCAGCCA<br>GAAGAGGAACCGGTCTTTTCTGCCCTTGGAGGGGAGGAGAGTACAAGTGGTGATAAGGAGGAATACAACCCCTGTTGCGGCGATCAGTGAGGATTTGGATTTGGTGAAAGTT<br>TTGCCGGAAAAGGCGGTAAAATTCAAGGTTAGGGCAGTTGTGACGGTGAGGAACAAGAACAAGGAGGATTTCAAGGAGACTTTGGTAAAACATTTGGACGCTTTTACGGAT<br>AAGATTGGGAGGAATGTTGTTTTGGAGCTCATCAGCACTGGTTTTGATCCAAAAACAAAGGCCCCAAAGAAGAGCAAAGAAGCTGTGTTGAAGGACTGGTCCAAGAAAGCA<br>AACCTCAAAACTGAGAAAGTGAATTACACAGCAGAATTTGAGGTGGATTCAAATTTTGGGATTCCTGGTGCAATTACAGTGGCCAATAAACACCAAAAGGAATTCTTTTTGG

Gene	Sequence Type	Sequence (5'-3')
		AGAGCATCACAATTGAAGGATTTGCATGTGGCCCCGTGCATTTCCCCTGCAATTCTTGGGTTCAGCCCAAGAAAGATCATCCTGGCAAAAGAATTTTCTTCACTAATAAGCCA<br>TATTTGCCACATGAAACACCTGAGGGATTAAAGGCTCTTAGAGAGAAGGAGCTTAGAGAATTGAGAGGTGATGGACAAGGTGAAAGAAAATTGTCTGATAGAGTGTATGAC<br>TTTGATGTGTACAATGATCTTGGCAATCCAGACAAGGGGATTGATTTCGCTCGACCGATTCTTGGTGGCGAAAAGATTCCGTATCCTAGGCGCTGTCGGACAGGGCGCTCTCC<br>CACGGATACCGATATGAACTCTGAGAGCCGAGTAGAGAAGCCGTTGCCTATGTATGTGCCTAGAGACGAGCAGTTTGAGGAGTCAAAGATGAATGCCTTTTCAACTGGGAGG<br>CTGAAGGGGGTCCTTCATAACCTCATACCATCGTTGATGGCGAGCATCTCTGCGAACAACAAAGATTTCAAAGGATTCTCGGACATCGACAGCCTGTACAGCGAAGGCCTTCT<br>CCTCAAACTCGGCCTCCACGATGAAATCATGAACAAGGTCCCATTGCCTAAATCTGTCAGCAAATTTCAAGAAGGTGGTCTGCTCAAATATGACCTTCCAAAGATTGTATCAA<br>AGGACAAATATGCATGGTTGAGGGATGACGAATTTGCCCGTCAAGCCATAGCCGGGGTGAATCCCGTCACCATTGAGAGGCTTCAATCTTTCCCCCCTGTGAGTAAGCTCGAC<br>CCCGAGATCTATGGACCTCAAGAATCAGCTCTCAAAGAAGAGCACATTGCTGGACAACTCAATGGCATGACCATACAAGAGGCTTTGGATGCAAACAAGCTCTTTATCGTTG<br>ATTACCACGATGTTTACTTGCCATTCATCGACAAGATCAATGCCCTTGATGGCCGGAAATCATATGCAACGCGGACTGTGTACTTCTTGACCGATGCTGGAACCCTCAAGCCC<br>ATAGCTATAGAGCTCAGCCTTGCACCAAGCTCTCCGAGGCCAAAACGCGTAGTCACACCTCCGGTCGACGCCACCACTTACTGGATGTGGCAACTGGCCAAAGCCCATGTCTG<br>CTCTAATGATGCTGGAGTTCATCAGCTTGTCAACCATTGGCTACGAACGCATGCAGCGTTGGAGCCATTCATATTGGCAGCTCATAGGCAGATGAGCGCAATGCATCCCATTT<br>TTAAGCTCTTGGATCCTCACATGAGGTACACGTTGGAGATCAAGCGTTGGCTCGTCAGAGCTTGA
SmLOX9	Protein	LTMPLTMLIKPVQRERRDSVLFDKIIDVAFDFISSRQQGSASRTPTTRVACTPARCARPPAEESIVRCEIVVIALSVPVDSQTKNVSRRSEGGVLCQIVTQTVLIGCCPFCDERFSQQKLSE<br>FTTNAVRAVVHFVMPEAGAIFNQETSHFESFEQIHRLFMGNGNQSVDEWLLKRLKHELPDDLFKKVKQVIKEKPVKFQLPRIAAGNQFAWMDDDEFGRQMLAGTNPTVISSLQNFPP<br>DGSNGVKSSIKGSDIEHNLDGLTVAEAMNQWRLFKLDHHDYLMPFLTRINRDEGVCAYASRTLLFLRDDCTLKPVAIELSLPGSLSHEEISRVFRPAHDGTEAALWQLAKAHVAAAD<br>SVYHHLVSHWLNAHAVVEPFIIATRRKLSVMHPIHRLLEPHFKDTMQVNALARSMLLNAGGIFEKILFTGDISMELSSFLYRSWRFDEQSLPADLIKRGFARYNPNQPENIELLFQDYP<br>YGADGLDIWIAIKNWVIEYCSYN-
	CDS	TTGACGATGCCACTAACGATGTTGATTAAACCTGTGCAAAGGGAACGTAGAGATAGTGTTCTATTTGATAAGATAATTGACGTGGCATTCGATTTCATATCTTCACGCCAGCA<br>AGGAAGCGCGAGTAGAACTCCAACGACTCGAGTGGCTTGTACTCCAGCACGGTGTGCCCGGCCCCCTGCGGAGGAGTCGATAGTTCGGTGTGAGATTGTAGTCATAGCCTTG<br>AGTGTACCTGTGGATTCACAGACGAAAAATGTGAGTCGACGTTCAGAAGGAGGAGTCTTGTGTCAGATCGTCACACAGACGGTGCTCATAGGCTGCTGTCCATTTTGTGATGA<br>ACGTTTCAGCCAACAGAAACTCTCGGAATTCACAACAAACGCGGTCAGGGCTGTTGTCCATTTTGTGATGCCTGAAGCAGGAGCAATCTTCAACCAAGAAACGAGCCACTTT<br>GAGTCCTTCGAGCAGATTCATAGGCTGTTCATGGGTAACGGAAATCAATCAGTTGACGAATGGTTGTTGAAGCGTTTAAAACACGAACTGCCTGATGATCTTTTCAAGAAGGT<br>AAAGCAAGTGATCAAGGAGAAACCTGTTAAGTTCCAATTGCCTCGGATTGCAGCAGGGAACCAATTTGCATGGATGGATGACGATGAATTTGGACGGCAGATGCTTGCTGGA<br>ACTAATCCAACTGTAATTTCATCTCTGCAGAATTTCCCACCAGACGGCAGCAATGGAGTCAAGAGTTCGATAAAGGGATCCGACATAGAGCATAACCTTGATGGTCTGACAGT<br>TGCAGAGGCAATGAACCAGTGGAGACTTTTCAAGCTAGATCACCACGATTATCTGATGCCATTCTTGACGAGAATAAACAGAGATGAGGGTGTATGTGCTTATGCATCGCGC<br>ACGCTACTCTTCTTGAGAGACGATTGCACGCTAAAACCAGTAGCCATAGAACTGAGCTTGCCTGGCTCTTTGTCCCACGAGGAAATTAGCAGAGTTTTCCGTCCAGCACATGA<br>CGGGACAGAAGCTGCTCTCTGGCAACTAGCAAAAGCTCATGTAGCAGCAGCAGATTCAGTTTACCATCATCTAGTCAGCCATTGGCTAAATGCACATGCTGTTGTTGAGCCAT<br>TTATCATTGCAACTCGAAGGAAGTTGAGTGTTATGCACCCCATTCACCGGTTACTCGAACCTCATTTCAAAGACACCATGCAAGTAAATGCACTGGCAAGGAGCATGTTGCTA<br>AATGCTGGAGGAATTTTTGAAAAGATACTGTTTACCGGTGACATCTCAATGGAGCTCTCGTCGTTTCTGTACAGAAGCTGGAGATTCGATGAGCAAAGCCTCCCTGCAGACCT<br>GATCAAAAGAGGCTTTGCCAGATATAACCCGAATCAGCCAGAAAACATCGAGCTTCTCTTTCAAGATTATCCCTATGGTGCAGATGGACTTGATATCTGGATAGCCATAAAGA<br>ACTGGGTGATAGAATACTGTTCTTACAATTAG
SmAOS1	Protein	MAASSSLSTLSCFSTSSLPSSSQTKPPRKVALRLITATLSEKPPTSKPAAPAKLPMRKIPGDYGLPLIGPWRDRQDYFYNQGRDEFFKSRIQKYQSTVYRTNMPPGPFISFRPNVVALLDG<br>KSFPALFDTDNVEKRDLFTGTFMPSTDLTGGYRTLSYLDPSEPNHAKLKNLMFFMLSSRRDHVIPEFHATYVQMFQGLEAELAAKGKASFGAANDVAAFNFLARSFYGVSPNESKLG<br>PDGPSIVAKWVLFQLHPLLRLGLPRPVEDGLLHTFTLPPFLIKKDYTRLYDFFYDNSSPILDHAETLGVARDEACHNLVFATCFNTFGGMKIFFPNMLKWVGRAGARLHADLAREIRS<br>AVKSSGAVSMGAMESMPLMKSVVYEALRIEPPVPSQYAKAKRDFVIESHEAAFQIREGEMLYGFQPLATKDPKIFDRAEEFVADRFVGEEGEKLLKHVLWSNGPENESPTLHNKQCA<br>GKDFVVLISRLLLVEFFLRYDSFDIEVAASPLGAAVTVTSLKRATF-
	CDS	ATGGCTGCTTCCTCTTCTCTATCCACTCTCTCTTGTTTCTCCACTTCCAGTCTCCCATCTTCTTCCCAAACCAAACCACCACGAAAAGTGGCTTTGCGGCTCATCACCGCCACCT<br>TATCGGAAAAGCCACCGACCTCGAAACCAGCGGCGCCGGCGAAACTCCCGATGAGAAAGATCCCCGGCGACTACGGGCTCCCCCTGATCGGGCCGTGGAGAGACAGGCAGG<br>ACTACTTCTACAACCAAGGCAGAGATGAATTCTTCAAATCAAGAATCCAGAAATACCAGTCCACCGTCTACAGAACCAACATGCCGCCGGGTCCCTTCATCTCCTTCCGCCCC<br>AACGTCGTCGCTCTCCTCGACGGCAAGAGCTTCCCGGCGCTCTTCGACACCGACAACGTCGAGAAAAGGGACCTCTTCACCGGCACCTTCATGCCCTCCACCGACCTCACCGG

Gene	Sequence Type	Sequence (5'-3')
		CGGCTACCGGACCCTCTCCTACCTCGACCCCTCCGAGCCCAACCACGCCAAGCTCAAGAACCTCATGTTCTTCATGCTCTCCTCCCGCCGCGACCACGTCATCCCGGAGTTCCA<br>CGCCACCTACGTCCAGATGTTTCAAGGTTTGGAGGCCGAGCTCGCCGCCAAAGGGAAGGCCAGCTTCGGCGCCGCAAACGACGTCGCCGCGTTTAACTTCCTAGCCCGATCCT<br>TCTACGGCGTCAGCCCGAACGAGAGCAAGCTGGGGCCCGACGGCCCGAGCATCGTCGCGAAATGGGTCCTCTTTCAGCTCCACCCGCTCCTCCGCCTCGGGCTGCCCCGACCC<br>GTGGAAGACGGGCTGCTCCACACCTTCACCCTCCCGCCGTTTTTGATCAAGAAGGACTACACGCGCCTCTACGACTTCTTCTACGACAACTCCTCGCCGATCCTGGACCACGC<br>CGAGACCCTGGGCGTGGCCCGCGACGAGGCCTGCCACAACCTCGTCTTCGCCACGTGCTTCAACACCTTCGGCGGCATGAAGATCTTCTTCCCCAACATGCTCAAGTGGGTCG<br>GCCGCGCCGGCGCCAGGCTCCACGCCGACCTGGCCAGGGAGATCCGCTCGGCCGTCAAATCCAGCGGCGCCGTCAGCATGGGGGCCATGGAGAGCATGCCGCTCATGAAGTC<br>GGTCGTGTACGAGGCGCTGCGGATCGAGCCGCCGGTGCCGTCGCAGTACGCCAAGGCGAAGCGCGACTTCGTGATCGAGTCCCACGAGGCGGCGTTCCAGATCAGGGAAGG<br>GGAGATGCTGTACGGGTTCCAGCCGCTAGCCACCAAGGACCCCAAGATATTCGACCGCGCCGAGGAGTTCGTGGCGGATCGGTTCGTCGGTGAGGAGGGGGAGAAGCTGCTC<br>AAGCACGTGCTCTGGTCAAACGGCCCGGAAAATGAGAGCCCCACGCTTCACAACAAGCAGTGCGCCGGCAAGGATTTTGTGGTGCTCATCTCACGGTTGCTGCTGGTTGAATT<br>CTTCCTCCGCTACGACTCCTTTGATATCGAGGTCGCCGCCTCTCCGTTGGGCGCCGCCGTAACTGTAACGTCGCTCAAACGGGCTACTTTCTAA
SmAOS2	Protein	MAASHSLSSLSPPHLQFSSQTSTRKLPAFSKPLRPQPIITATISERPYVSQPLPPPLTAATPSTLPIRKIPGDYGLPLIGPWKDREDFFYKQGRDEFFKSRIKKYQSTVFRTNMPPGPFISFNP<br>NVVALLDGKSFPALFDTDKVEKKDLFTGTYMPSVELTGGYRTLSFLDPSEPNHAKLKKLMFFMLSHRRERVMPEFHSSYTELFDNLERELASKGKADFGPPSDQAAFNFLARSFFGV<br>DPRDTKLGADGPKLVTKWVLFQLHPLLTLGLPRPVEDGLLHNFMLPPTLIKKDYQRLYDFFLGNSAALLDQAEKLGLSRDEACHNLLYTTCFNSFGGMKKLFPNMLKWVARAGTR<br>LHTDLAREIRAAIASRGGGGVTMAAMEGMPLMKSVVYEALRVEPPVASQYAKAKRDFVVESHDAAFRIKEGEMLFGFQPFATKDPRIFSRAEEFVPDRFVGEEGERLLKHVVWSNG<br>PESGHATVNNKQCAGKDFVVLAARLLLVELFRRYDSFDIEVAASPLGSDITLTSLKRASF-
	CDS	ATGGCTGCTTCGCATTCTCTCTCATCTCTCTCTCCTCCCCACCTCCAATTCTCTTCCCAAACCTCTACAAGAAAGCTACCCGCTTTCTCAAAACCCTTGAGGCCTCAGCCGATCA<br>TCACCGCCACCATATCCGAGAGACCATACGTTTCACAGCCACTTCCGCCGCCACTGACGGCGGCGACGCCCTCCACGCTACCCATCCGCAAAATCCCGGGAGATTACGGGCTT<br>CCCTTGATCGGGCCATGGAAGGACCGAGAAGACTTCTTCTACAAGCAAGGGCGCGACGAATTCTTCAAATCGAGAATCAAGAAATACCAATCCACTGTGTTCCGAACCAACA<br>TGCCACCCGGCCCCTTCATCTCCTTCAACCCGAACGTCGTCGCCTTGCTCGACGGCAAGAGTTTTCCGGCGCTCTTCGACACCGATAAAGTCGAGAAGAAGGACCTCTTCACC<br>GGCACCTACATGCCCTCCGTGGAGCTCACCGGCGGCTACCGCACCCTCTCCTTCCTCGACCCATCGGAGCCCAACCACGCCAAGCTCAAGAAGCTCATGTTCTTCATGCTCTCC<br>CACCGCCGCGAACGCGTCATGCCGGAGTTCCACAGCAGTTACACCGAGCTGTTCGACAACTTAGAGAGAGAATTGGCCTCCAAGGGAAAAGCCGACTTCGGGCCGCCCAGCG<br>ATCAGGCGGCGTTCAATTTCTTGGCCCGCTCCTTCTTCGGAGTCGACCCGAGGGACACCAAACTCGGGGCGGACGGGCCGAAGCTCGTCACGAAATGGGTACTGTTTCAGCTG<br>CATCCGCTGCTCACTCTGGGCCTGCCGCGGCCCGTTGAAGATGGGCTCTTACACAACTTCATGCTGCCGCCGACGTTGATCAAGAAGGATTATCAAAGGCTATACGACTTCTT<br>CCTCGGCAACTCCGCCGCGCTCCTCGACCAAGCCGAGAAGCTGGGGCTCTCCCGCGACGAGGCCTGCCACAATCTGCTCTACACCACGTGCTTCAACTCCTTCGGCGGCATGA<br>AGAAGCTCTTCCCCAACATGCTCAAGTGGGTCGCCCGCGCCGGGACCAGGCTCCACACGGATCTCGCCAGGGAGATCCGGGCGGCGATCGCGTCCCGCGGCGGCGGCGGCGT<br>GACCATGGCGGCGATGGAGGGCATGCCGCTGATGAAGTCGGTGGTGTACGAGGCGCTGCGCGTGGAGCCGCCCGTGGCGTCGCAGTACGCCAAGGCGAAGCGCGACTTCGT<br>GGTGGAGTCGCACGACGCGGCGTTCCGGATCAAGGAGGGGGAGATGCTGTTCGGCTTCCAGCCCTTCGCCACCAAGGACCCCAGGATTTTCAGCCGGGCCGAGGAGTTCGTG<br>CCGGACCGGTTCGTCGGCGAAGAAGGGGAGAGGCTCTTGAAGCACGTGGTCTGGTCCAACGGCCCGGAGAGCGGCCACGCCACCGTCAACAACAAGCAGTGCGCCGGGAAG<br>GACTTTGTCGTGCTGGCGGCCAGGCTGCTGCTGGTGGAGCTCTTCCGCCGCTACGACTCATTTGATATTGAGGTTGCGGCATCGCCGTTGGGCTCCGACATCACATTGACGTCG<br>TTAAAACGGGCAAGCTTTTAG
SmAOS3	Protein	MSSVDELPLKEIPGGYGLPFFGPISDRLDFHYRQGPDEFFRARMQQHNSTVFRANAPPGPFTARNPKVVVLLDAASFQILFDTSKVEKRDVFTGTFMPSTSFTGGYRVCSYLDPSEPKH<br>ALLKGFFLSVLGRLHKELIPTFRAAATQLFADVEAELAEKGESEFNPISDRMSFDLLFRLLPARARLKFLPNFVEDLLLHTFPLPYFPAKSGYELVHSAFREAAGELLDEAEEKGLSRDE<br>ASHNMMFVMGFNSYGGTKILFPALLKYVAGGGEDLHRRLASEIRRVVEEEGGVTLAALERMSLVKSVVWETMRIEPPVQFQYGKAKEDLKISSHVATYVVKKGETIFGYQPFATRD<br>PVIFVNPDEFVPDRFLDGGEKLLKYVYWSNGRETDSPTADNKQCPAKDMVVVLGRMMLVELFMRYETFSVEFGKLLLGSSVTIKSLTNPK-
	CDS	ATGTCTTCAGTCGACGAGCTCCCACTGAAAGAAATCCCCGGCGGCTACGGCCTCCCATTCTTCGGACCGATATCGGACCGCCTCGACTTCCATTACCGTCAGGGCCCGGACGA<br>GTTCTTCCGGGCCCGAATGCAGCAGCACAACTCCACCGTCTTCCGGGCCAACGCCCCGCCCGGCCCTTTCACCGCCCGAAACCCTAAAGTCGTCGTCCTCCTCGACGCCGCGA<br>GCTTCCAGATCCTCTTCGACACGTCCAAGGTCGAGAAGCGGGACGTCTTCACCGGCACCTTCATGCCGTCTACTAGCTTCACCGGCGGCTACCGCGTCTGCTCCTACCTCGACC<br>CCTCCGAGCCCAAGCACGCCCTCCTCAAGGGCTTCTTCCTCTCTGTTCTCGGCAGATTGCACAAGGAATTGATCCCGACCTTCCGCGCCGCCGCTACGCAGCTCTTTGCCGACG<br>TCGAGGCTGAGCTCGCCGAGAAAGGGGAGTCGGAATTCAACCCGATCAGCGACAGGATGTCGTTCGATCTCTTGTTCCGTTTATTGCCGGCAAGAGCTCGGTTGAAGTTCCTC<br>CCTAATTTCGTCGAGGATTTGCTCCTCCACACGTTCCCGCTGCCGTACTTTCCAGCGAAATCGGGGTACGAGCTGGTCCACAGCGCGTTCCGGGAGGCTGCCGGAGAGTTGCT

Gene	**Sequence Type**	**Sequence (5'-3')**
		GGACGAGGCGGAGGAGAAGGGTTTGAGTAGGGATGAGGCTTCTCACAACATGATGTTTGTGATGGGCTTTAACTCCTATGGCGGGACGAAGATACTCTTCCCGGCTTTGTTG<br>AAGTACGTCGCCGGCGGCGGGGAGGACTTGCACCGCCGCCTCGCCAGCGAGATCCGGAGGGTCGTGGAGGAGGAGGGCGGCGTGACTCTGGCGGCGCTGGAGAGGATGAGC<br>CTGGTGAAATCTGTGGTATGGGAGACGATGAGGATTGAACCGCCGGTTCAGTTTCAATATGGCAAGGCTAAAGAGGATTTGAAAATTTCGAGCCACGTGGCAACTTACGTGG<br>TAAAAAAGGGGGAGACCATATTCGGGTACCAACCCTTTGCAACGAGGGATCCCGTGATATTCGTAAACCCGGACGAGTTCGTGCCTGATAGGTTCTTGGACGGGGGCGAAAA<br>GTTATTAAAGTACGTGTATTGGTCTAACGGGAGAGAGACGGACAGCCCGACGGCGGACAACAAGCAATGTCCGGCGAAGGATATGGTGGTGGTGCTGGGGAGGATGATGTT<br>GGTGGAGCTTTTCATGCGGTATGAGACGTTTAGTGTGGAATTTGGGAAGCTGCTTCTTGGTTCATCTGTCACCATCAAATCGCTCACAAACCCCAAATAG
SmAOS4	Protein	MSTAQLPAREIPGEHGLPFFGPIKDRFSYFYSQGELKYFTTRMDKYQSTVFRCNMPPGPFMARDPRVVCLLDAVSFQTLFDTTKVDKRDVLDGTFMPSTAFTGGYRTCAYLDPSEPN<br>HAILKGFFLSILAKKHDQFIPLFRQAMSGLFTGLEGDLAAKGSSSFNDLNDVMAFEFIFRLLCGVGPSETGLGPDGPKSMDLWLFGQLSPLMTLGIKYVPGFLEDFFLHNFPIPFFLVRS<br>YYSKIYAAFEKNAGKLLEEAEELGLQRDEACHNLVFLAGFNAYGGMKAVFPALIKWVAAAGADLHRRLAAEIRAAVKEKGVTPAALNELTLTKSVVYEAMRMEPSVPYQYGRA
	CDS	ATGTCCACAGCCCAGCTTCCGGCCCGAGAAATCCCCGGCGAGCACGGGCTCCCGTTTTTCGGGCCCATAAAAGACCGTTTCAGCTACTTCTACAGCCAAGGCGAGCTCAAATA<br>CTTCACAACCCGCATGGACAAATACCAATCCACGGTGTTCCGATGCAACATGCCGCCGGGCCCCTTCATGGCCCGAGACCCGCGCGTCGTCTGCCTCCTCGACGCCGTCAGCT<br>TCCAGACCCTCTTCGACACCACCAAAGTTGACAAGAGAGACGTCCTCGACGGCACCTTCATGCCGTCCACCGCCTTCACCGGCGGCTACCGGACCTGCGCCTACCTCGACCCC<br>TCCGAGCCCAACCACGCAATTCTCAAAGGCTTCTTCCTCTCAATACTCGCCAAGAAGCACGACCAATTCATCCCGTTGTTCCGGCAGGCCATGTCCGGCCTTTTTACGGGCCTC<br>GAAGGTGACCTCGCCGCCAAAGGCAGCTCCTCCTTCAACGATTTAAACGACGTCATGGCGTTCGAGTTCATCTTCCGCTTGCTCTGCGGCGTGGGCCCGTCGGAGACCGGGCT<br>CGGCCCGGACGGCCCGAAATCGATGGACCTGTGGCTCTTCGGGCAGCTCTCCCCGCTGATGACTCTCGGGATCAAATACGTCCCGGGCTTTCTAGAAGATTTCTTCCTCCACA<br>ATTTCCCCATCCCTTTCTTCCTCGTCAGATCTTACTACAGCAAGATTTACGCAGCTTTCGAGAAGAACGCGGGGAAGCTTCTAGAAGAAGCGGAGGAACTCGGGTTACAACGC<br>GACGAAGCGTGTCACAACCTCGTGTTCCTCGCCGGCTTCAACGCGTACGGCGGGATGAAAGCCGTGTTCCCGGCGCTCATCAAGTGGGTCGCCGCCGCGGGCGCCGACCTCC<br>ACCGCCGGCTCGCGGCGGAGATCAGGGCGGCGGTGAAGGAAAAGGGGGTGACTCCGGCGGCGTTGAACGAGCTGACGCTGACAAAATCGGTGGTGTACGAGGCTATGCGCA<br>TGGAGCCGTCGGTTCCGTACCAATACGGGAGAGCTAA
SmAOS5	Protein	MSSVDELPLKEIPGGYGLPFFGPISDRLDFHYRQGPDEFFRARMQQHNSTVFRANAPPGPFTARNPKVVVLLDAASFQILFDTSKVEKRDVFTGTFMPSTSFTGGYRVCSYLDPSEPKH<br>ALLKGFFLSFLGRLHKELIPTFRAAATQLFADVEAELAEKGESEFNPISDRMSFDLLFRLFAGKSSYDTAAGGGGNAGLDTWLFLQLAPLITLGLKFLPNFVEDLLLHTFPLPYFPAKSG<br>YELVHSAFREAAGELLDEAEEKGLSRDEASHNMMFVMGFNSYGGTKILFPALLKYVAGGGEDLHRRLASEIRRSWRRRAA-
	CDS	ATGTCTTCAGTCGACGAGCTCCCACTGAAAGAAATCCCCGGCGGCTACGGCCTCCCATTCTTCGGACCGATATCGGACCGCCTCGACTTCCATTACCGTCAGGGCCCGGACGA<br>GTTCTTCCGGGCCCGAATGCAGCAGCACAACTCCACCGTCTTCCGGGCCAACGCCCCGCCCGGCCCTTTCACCGCCCGAAACCCTAAAGTCGTCGTCCTCCTCGACGCCGCGA<br>GCTTCCAGATCCTCTTCGACACGTCCAAGGTCGAGAAGCGGGACGTCTTCACCGGCACCTTCATGCCGTCTACTAGCTTCACCGGCGGCTACCGCGTCTGCTCCTACCTCGACC<br>CCTCCGAGCCCAAGCACGCCCTCCTCAAGGGCTTCTTCCTCTCTTTTCTCGGCAGATTGCACAAGGAATTGATCCCGACCTTCCGCGCCGCCGCTACGCAGCTCTTTGCCGACG<br>TCGAGGCTGAGCTCGCCGAGAAAGGGGAGTCGGAATTCAACCCGATCAGCGACAGGATGTCGTTCGATCTCTTGTTCCGTTTATTCGCCGGCAAGAGCTCGTACGACACCGCC<br>GCAGGCGGCGGCGGCAATGCCGGCCTCGACACGTGGCTGTTTCTGCAGCTGGCTCCATTGATCACGCTAGGGTTGAAGTTCCTCCCTAATTTCGTCGAGGATTTGCTCCTCCAC<br>ACGTTCCCGCTGCCGTACTTTCCAGCGAAATCGGGGTACGAGCTGGTCCACAGCGCGTTCCGGGAGGCTGCCGGAGAGTTGCTGGACGAGGCGGAGGAGAAGGGTTTGAGTA<br>GGGATGAGGCTTCTCACAACATGATGTTTGTGATGGGCTTTAACTCCTATGGCGGGACGAAGATACTCTTCCCGGCTTTGTTGAAGTACGTCGCCGGCGGCGGGGAGGACTTG<br>CACCGCCGCCTCGCCAGCGAGATCCGGAGGTCGTGGAGGAGGAGGGCGGCGTGA
SmAOS6	Protein	MAIPKPESLGLGLKIYNPKKISNPISPHPTNPEPDRASPKTRWAGPIDIPRYIY*RAGTRLHTDLAREIRAAIASRGGGGVTMAAMEGMPLMKSVVYEALRVEPPVASQYAKAKRGLTV<br>VESHDAAFRIKEGEMLFGFQPFATKDPRIFSRAEEFVPDRFVGEEGERLLKHVVWSNGPESGHATVNNKQCAGKDFVVLAARLLLVELFRRYDSFDIEVAASPLGSDITLTSLKTGKL<br>LDCGRGC-
	CDS	ATGGCAATCCCGAAACCCGAAAGTTTAGGGTTAGGGTTGAAGATTTACAACCCGAAAAAAATCTCCAACCCGATTAGCCCGCACCCGACTAACCCGGAACCCGATAGGGCTA<br>GCCCGAAAACCCGGTGGGCTGGCCCGATTGACATCCCTAGATACATCTATnCCCGCGCCGGGACCAGGCTCCACACGGATCTCGCCAGGGAGATCCGGGCGGCGATCGCGTC<br>CCGCGGCGGCGGCGGCGTGACCATGGCGGCGATGGAGGGCATGCCGCTGATGAAGTCGGTGGTGTACGAGGCGCTGCGCGTGGAGCCGCCCGTGGCGTCGCAGTACGCCAA<br>GGCGAAGCGCGGACTTACGGTGGTGGAGTCGCACGACGCGGCGTTCCGGATCAAGGAGGGGGAGATGCTGTTCGGCTTCCAGCCCTTCGCCACCAAGGACCCCAGGATTTTC<br>AGCCGGGCCGAGGAGTTCGTGCCGGACCGGTTCGTCGGCGAAGAAGGGGAGAGGCTCTTGAAGCACGTGGTCTGGTCCAACGGCCCGGAGAGCGGCCACGCCACCGTCAAC<br>AACAAGCAGTGCGCCGGGAAGGACTTTGTCGTGCTGGCGGCCAGGCTGCTGCTGGTGGAGCTCTTCCGCCGCTACGACTCATTTGATATTGAGGTTGCGGCATCGCCGTTGGG

Gene	Sequence Type	Sequence (5'-3')
		CTCCGACATCACATTGACGTCGTTAAAAACGGGCAAGCTTTTAGATTGCGGGAGGGGTTGCTGA
SmAOS7	Protein	MLDKWLAVQLLPTISINVVQPLEEIFLHSFSYPFWLVKSDYEKLASFVETGAGEAVRRAQTEFKLTKEEAIHNLIFMLGFNAFGGFSLFFLSLLGNLGEQEPSVHEELRREVREKLSNN<br>VLSFETVKKMDLVNSFVYETLRLDPPVPQQFARARKDFDLGSHNAVYEIKKGELLCGFQPLVMRDPKIFENPEDFVYNRFSKVKGGDELLEYLFWSNGPQTGTPGPSASNKQCVARD<br>AVPQTAAVFLAYLFQRYDEISISSGSITALKKAK-
SmAOS7	CDS	ATGCTTGACAAATGGCTCGCCGTGCAGCTCCTCCCAACGATAAGCATCAACGTCGTGCAGCCCCTCGAGGAGATCTTCCTCCACTCGTTTTCCTACCCCTTCTGGCTCGTCAAA<br>TCCGACTACGAGAAGCTCGCGAGTTTCGTCGAGACCGGAGCCGGAGAGGCCGTGCGAAGAGCGCAAACTGAGTTCAAGCTCACCAAGGAGGAGGCCATCCACAACCTCATC<br>TTCATGCTCGGGTTCAACGCGTTTGGAGGGTTCTCCCTCTTCTTCCTCTCGCTCCTCGGCAACCTCGGGGAGCAGGAGCCGAGCGTGCACGAGGAGCTGAGGAGGGAGGTACG<br>GGAGAAGCTCTCGAACAACGTCCTTAGCTTTGAAACGGTGAAGAAGATGGACCTGGTGAATTCGTTCGTGTACGAGACACTGCGTTTGGATCCACCAGTCCCGCAGCAGTTC<br>GCCCGAGCTCGAAAGGACTTCGACCTCGGCTCGCACAACGCCGTGTACGAGATCAAGAAAGGGGAACTGCTGTGTGGCTTTCAGCCACTAGTGATGAGGGATCCTAAAATAT<br>TTGAGAATCCAGAGGACTTTGTGTACAACAGATTCTCAAAGGTAAAAGGTGGTGATGAGTTGCTGGAATACCTGTTCTGGTCGAACGGGCCGCAGACGGGGACTCCGGGGCC<br>GTCGGCCTCCAACAAGCAGTGCGTGGCCAGGGACGCCGTGCCTCAAACTGCTGCTGTGTTTCTGGCTTACCTGTTTCAGAGATATGATGAGATCTCAATTTCTTCTGGCTCCAT<br>CACTGCTCTCAAAAAGGCAAAGTGA
SmAOC1	Protein	MAFTNSITPKAEAPSALPSTGNRIADLRFSTSVSTKICNTPNQRSISCARSSTPQAFFGLWSKPKPEPSTPSKVQELYVYEMNERDRGSPAYLRLSQKEQNTLGDLVPFSNKLYSGDLQK<br>RLGITAGLCVLIQHFPDQNADRYEAIYSFYFGDYGHISVQGAYLTRSDTYLAVTGGSGVFEGVSGTVKLQQIVFPFKLFYTFYLKGIKDLPAELVVTPVAPSPSVEPSPAAKAAEPQATI<br>ANFTN-
SmAOC1	CDS	ATGGCTTTCACTAACTCCATCACTCCCAAGGCTGAAGCACCTTCTGCTCTCCCATCCACCGGAAATCGGATCGCCGATCTTCGTTTCTCGACCTCCGTTTCAACCAAAATTTGC<br>AATACTCCGAATCAGAGATCGATTTCCTGCGCCAGATCTTCCACGCCGCAAGCCTTCTTCGGCCTCTGGAGCAAACCCAAGCCGGAGCCTTCAACTCCATCTAAGGTTCAGGA<br>GCTGTACGTGTACGAGATGAACGAGCGCGACCGCGGCAGCCCGGCCTACCTACGGCTGAGCCAGAAGGAGCAGAACACCCTGGGCGACCTCGTCCCCTTCTCCAACAAGCTC<br>TACTCCGGCGACCTGCAGAAGAGATTGGGCATCACGGCCGGTCTCTGCGTGCTCATCCAGCACTTTCCCGACCAGAATGCCGACCGCTACGAGGCCATCTACAGCTTCTATTT<br>CGGCGATTACGGCCACATTTCGGTGCAGGGCGCGTACCTCACACGCTCCGACACTTACCTGGCCGTCACCGGCGGCTCCGGCGTCTTTGAGGGTGTGTCTGGCACGGTCAAGC<br>TGCAGCAGATAGTGTTCCCGTTTAAGCTCTTCTACACGTTTTATTTGAAGGGGATCAAGGATTTGCCGGCGGAGCTGGTGGTCACCCCGGTGGCGCCGTCGCCCTCCGTCGAG<br>CCGTCGCCGGCTGCCAAGGCCGCCGAGCCTCAGGCCACCATTGCTAACTTCACAAATTGA
SmAOC2	Protein	MAASSASTILKVAVSSPSPARLPPSAASQKLPSKQKHSLPKPLALSTTKSFSCRAQAAADSTPRPQKVQELHVYEINERDRGSPAYLRLSQKTVNSLGDLVPFSNKVYTGDLKKRAGIT<br>AGICILIKNEAEKKGDRYEAIYSFYLGDYGHIAVQGPYLTYQDTELAVTGGSGVFEGVYGHVKLHQIIFPFKLFYTFHLKGIPDLPPELLAQPVPPALHVEPTPAAKTCEPGATLPNFTN
SmAOC2	CDS	ATGGCAGCTTCATCCGCCTCTACTATTTTGAAGGTTGCCGTCTCCTCCCCTTCTCCCGCCAGACTGCCGCCGTCCGCCGCCTCCCAGAAACTCCCATCCAAGCAGAAACACTCC<br>CTCCCTAAACCCCTCGCTCTCTCCACCACTAAATCATTCTCATGCAGAGCACAGGCTGCAGCTGATTCAACACCCCGTCCCCAGAAAGTTCAAGAGCTGCACGTCTACGAGAT<br>CAACGAGCGTGATCGCGGCAGCCCCGCATACCTCCGATTGAGCCAAAAAACCGTCAATTCCCTCGGCGATCTCGTGCCTTTCAGCAACAAGGTGTACACCGGCGACCTGAAG<br>AAACGGGCTGGAATAACGGCGGGGATATGCATCCTGATAAAGAACGAAGCAGAGAAGAAGGGCGACCGGTACGAGGCCATCTACAGCTTCTACTTGGGCGACTACGGCCAC<br>ATCGCCGTGCAGGGGCCCTACCTCACCTACCAAGACACCGAGCTCGCCGTCACCGGCGGCTCCGGCGTCTTCGAGGGCGTCTACGGCCACGTCAAGCTCCACCAGATCATCTT<br>CCCCTTCAAACTCTTCTATACCTTCCACCTCAAGGGCATCCCCGACCTGCCGCCGGAGCTGCTCGCCCAGCCGGTGCCGCCCGCGCTCCACGTGGAGCCCACCCCCGCCGCCA<br>AGACTTGTGAACCGGGAGCCACGCTCCCTAACTTTACCAATTAG
SmOPR3-1	Protein	LAITSKLQQSSLSKAAMVETKPSLFSPYKMGQYNLSHRVVLAPMTRCRALNAMPNSALAEYYEQRATDGGFLITEGTMISPYSAGFPHVPGIFNKEQVEAWKPVVERVHAKGAVIFC<br>QLWHVGRASHPELQPGGIAPISSTDKPISKRWRVLLPNGKYEMYPKPRKLETHELPELVHQYRQSAINAIEAGFDGIEIHGAHGYLLDQFMKDGINERTDEYGGSLQNRCKFIIQVVEA<br>VVFAIGADRVGVRISPAIDHLDAMDSNPHSLGLAVIERLNKLQFACGSKLAYLHITQPRYTAYGQTESGRPGSEEEEAQLLRTWRNAYQGTFICSGGFTRQLGIEAVAVGDADLVAYG<br>RLFISNPDLVERLKLNAPLNKYVRATFYTHDPVVGYTDYPFLQSNAEKPATISRL-
SmOPR3-1	CDS	TTGGCGATCACGTCGAAATTGCAGCAATCATCTCTCAGTAAAGCAGCCATGGTGGAAACGAAGCCGTCTCTCTTCTCCCCTTACAAGATGGGCCAGTACAATCTCTCCCACAG<br>GGTGGTTTTGGCGCCGATGACTCGATGCAGAGCGCTGAACGCCATGCCTAATTCCGCCCTAGCTGAGTACTACGAGCAGAGAGCCACGGACGGCGGATTTCTGATCACGGAG<br>GGCACCATGATCTCCCCCTATTCCGCAGGGTTCCCGCACGTGCCGGGCATCTTCAACAAGGAGCAAGTGGAGGCGTGGAAGCCGGTGGTGGAGAGGGTGCATGCTAAGGGCG<br>CTGTTATCTTCTGTCAGCTCTGGCATGTCGGTCGAGCTTCGCATCCAGAACTTCAGCCTGGTGGTATTGCGCCAATCTCCTCAACGGACAAGCCGATATCCAAAAGGTGGAGA

Gene	Sequence Type	**Sequence (5'-3')**
		GTGCTACTTCCGAACGGGAAATACGAGATGTATCCCAAGCCCCGCAAATTGGAAACTCACGAATTGCCTGAGCTCGTACACCAATATCGCCAGTCGGCCATAAACGCCATTG<br>AAGCAGGATTTGATGGCATTGAGATCCACGGAGCTCACGGTTACCTCCTCGATCAATTTATGAAGGATGGGATCAACGAGCGGACAGATGAGTACGGTGGATCCCTCCAAAA<br>CCGCTGCAAATTCATTATACAGGTAGTTGAAGCTGTTGTTTTTGCTATTGGTGCTGATCGAGTAGGCGTCAGAATATCACCTGCAATCGACCATCTTGATGCAATGGACTCCAA<br>TCCACACAGCCTAGGACTAGCTGTGATTGAGAGACTCAACAAACTGCAGTTTGCCTGTGGTTCCAAACTCGCCTATCTACACATCACTCAGCCACGATACACAGCCTATGGGC<br>AGACAGAGTCGGGCAGGCCTGGCAGCGAAGAGGAGGAGGCACAGCTGTTGAGAACTTGGCGAAATGCATATCAAGGAACATTCATTTGTAGTGGTGGGTTCACCAGGCAGC<br>TAGGTATTGAAGCAGTGGCGGTGGGGGATGCTGACTTGGTGGCCTACGGCCGGCTCTTCATTTCAAACCCGGATTTGGTCGAGAGACTGAAGCTCAACGCACCTTTAAACAA<br>GTATGTGAGGGCTACGTTCTATACTCACGATCCCGTTGTGGGATATACCGACTACCCCTTCCTGCAATCTAATGCTGAAAAACCTGCAACCATATCGCGGCTTTGA
SmOPR3-2	Protein	MAAEDTDVIPLLTPHKMGEFTLSHRIVLAPLTRDRSYNNVPQPHAALYYSQRATKGGLLITEATGVSDTAQGYPDTPGIWTKEHVEAWKPIVEAVHQKGGVFFVQLWHVGRVSTYD<br>YQPGGEAPLSSTDKGLTPGLYGVDWSPPRKLNTDEIRGIVNDFRAAARNAIEAGFDGVEIHGANGYLLEQFMKDQVNDRTDEYGGSLENRCRFALEVAAAVSDEIGSHRVGMRLSP<br>YTDFNEAVDSDPDGLGLYMANALSKLDLVYLHVIEPRAVGSTEIEGCAEDQLLPMRKAFSNTFISAGGFDRAKGNCAVDHDAADLVAYGRLFLANPDLPERYRLDAPLNKYDRNTF<br>YTSHPVIGYTDYPFLQQASIV-
	CDS	ATGGCAGCTGAAGATACTGATGTTATCCCCCTGCTCACCCCACACAAAATGGGGGAATTCACACTCTCCCACAGAATAGTATTAGCACCATTAACCAGAGACCGCTCATACAA<br>CAACGTTCCACAGCCGCATGCCGCATTGTATTACTCTCAGCGAGCAACCAAAGGCGGCCTCCTCATCACTGAAGCAACAGGTGTATCAGATACTGCTCAAGGGTACCCGGAT<br>ACACCTGGAATTTGGACAAAGGAGCATGTGGAGGCATGGAAGCCTATAGTGGAAGCAGTCCATCAAAAGGGTGGTGTCTTCTTCGTGCAGTTGTGGCATGTTGGCCGTGTCT<br>CCACTTATGATTACCAGCCTGGTGGCGAAGCTCCACTCTCATCAACAGACAAGGGATTGACCCCTGGCTTATACGGAGTGGATTGGTCTCCTCCCAGAAAACTAAACACCGAC<br>GAAATCCGTGGCATCGTCAACGATTTCAGGGCGGCTGCTCGCAACGCCATCGAAGCAGGATTCGACGGAGTCGAGATACACGGCGCCAACGGCTACTTACTGGAGCAGTTCA<br>TGAAGGATCAAGTGAACGACAGGACAGACGAATACGGCGGCAGCCTCGAGAACAGGTGCCGCTTCGCTCTGGAGGTGGCGGCGGCTGTGAGCGACGAGATAGGATCGCACA<br>GAGTGGGGATGCGGCTGTCTCCCTACACCGATTTCAACGAGGCGGTGGACTCGGATCCGGACGGCCTCGGGCTCTACATGGCCAATGCGCTCAGCAAGCTCGACCTCGTGTA<br>CCTCCACGTCATCGAGCCGCGAGCTGTTGGATCGACGGAGATCGAAGGGTGTGCCGAAGATCAGCTCCTCCCCATGAGGAAGGCCTTCAGCAACACCTTCATCTCTGCCGGTG<br>GCTTCGACAGGGCTAAGGGGAATTGCGCCGTGGATCACGACGCCGCCGATTTGGTGGCGTACGGCCGCCTCTTCTTGGCGAATCCGGATTTGCCGGAGAGGTATCGCTTGGAT<br>GCGCCGCTTAACAAGTATGATAGGAACACTTTCTACACTTCTCATCCTGTGATTGGTTACACTGATTATCCATTTCTTCAACAAGCTAGCATAGTGTAG
SmOPR3-3	Protein	MGEFTLSHRIVLAPLTRARSYDTVPQPHAALYYSQRATKGGLLITEATTVSDTAKGFPDTPGIWTKEHVEAWKPIVDAVHQKGAVFFLQLWHVGRASTYDYQPGGQAPVSSTNKGL<br>TPGLEGVDWSPPRKLTTEEIRGVVNDFRVAARNAIEAGFDGVEIHGANGYLLEQFMKDQVNDRTDEYGGSLEKRCRFALEVAAAVIDEIGSHRVGMRLSPFSDYNESVDSDPDGLGL<br>YMANALSELDLVYLHVIEPRAVGATVIEGCAEGQLLPMRKAFGNTFIAAGGFDRAKGNSAIDDGAADLVAYGRLFLANPDLPERYRLDAPLNKYDRNTFYTSHPVIGYTDYPFLQQ<br>AL-
	CDS	ATGGGGGAATTCACACTCTCCCACAGAATAGTATTAGCACCATTAACCAGAGCTCGGTCATACGACACCGTTCCACAGCCGCATGCTGCCTTGTATTACTCCCAGCGAGCAAC<br>CAAAGGCGGCCTCCTCATCACTGAAGCAACAACTGTATCAGATACTGCCAAAGGGTTCCCAGATACACCTGGAATCTGGACTAAGGAACATGTGGAGGCATGGAAGCCTATA<br>GTGGATGCAGTCCATCAAAAGGGTGCTGTTTTCTTCCTGCAGTTGTGGCATGTTGGCCGTGCCTCCACTTATGATTACCAGCCAGGCGGCCAAGCTCCAGTCTCATCAACAAA<br>CAAGGGATTGACCCCTGGCTTAGAAGGAGTAGATTGGTCTCCTCCCAGAAAACTAACCACCGAAGAAATCCGTGGCGTCGTCAACGATTTCAGGGTGGCTGCTCGCAACGCT<br>ATCGAAGCAGGATTCGACGGAGTCGAGATACACGGCGCCAACGGCTACTTACTGGAGCAGTTCATGAAGGATCAAGTGAACGACAGGACAGACGAATACGGCGGCAGCCTC<br>GAGAAGAGGTGCCGCTTCGCTCTGGAGGTGGCGGCGGCGGTGATCGACGAGATAGGATCGCACAGAGTGGGGATGAGACTGTCTCCATTCTCCGATTACAACGAGTCAGTGG<br>ACTCGGATCCAGACGGCCTCGGGCTCTACATGGCCAATGCGCTCAGCGAGCTCGACCTCGTGTACCTCCACGTCATCGAGCCGCGAGCTGTTGGCGCGACGGTGATCGAAGG<br>GTGTGCCGAAGGCCAGCTCCTCCCCATGAGGAAGGCCTTCGGCAACACCTTCATCGCTGCCGGTGGCTTCGACAGGGCTAAGGGGAATTCCGCTATAGATGATGGCGCCGCC<br>GATTTGGTGGCGTACGGCCGCCTCTTCTTGGCGAATCCGGATTTGCCGGAGAGGTATCGCTTGGATGCGCCGCTTAACAAGTATGATAGGAACACTTTCTACACTTCTCATCCT<br>GTGATTGGTTACACTGATTATCCATTTCTTCAACAAGCATTATAG
SmOPR3-4	Protein	MAAEDTDVIPLLTPHKMGEFTLSHRFKFLTTLYSISYNSSISLCIYLINWVALNRIVLAPLTRNRSYNNVPQPHAALYYSQRATKGGLLITEGTGVSDTAQGYPDTPGIWTKEHVEAWK<br>PIVEAVHQKGAVFFVQLAHVGRVSTYDYQPGGEAPVAPTDKALTPGLYGVDWSPPRKLTTDEIHGIVNDFRLAARNAIEAGFDGVELHGANGFLLEQFMKDQVNDRTDEYGGSLEK<br>RCRFALEVAAAVSDEIGSHRVGMRLSPYTDFNEAVDSDPDGLGLYMANALSKLDLVYLHVIEPRGEGATASEGCGEDQLLPMRKAFSNTFIVADGFDRTKGNCAVDDDAADLVAY<br>GRLFLANPDLPERYRLDAPLNKYDRNTFYTSHPVVGYTDYPFLQQP-
	D	ATGGCAGCTGAAGACACTGATGTTATTCCTCTGCTCACCCCCCACAAAATGGGGGAATTCACACTCTCCCACAGGTTTAAATTCCTTACTACATTATACTCCATATCTTATAAT

Gene	Sequence Type	Sequence (5'-3')
		TCTTCAATTTCTTTGTGTATATATCTCATTAATTGGGTGGCGTTGAACAGAATAGTATTAGCACCATTAACCAGAAACCGATCATACAACAACGTTCCACAGCCGCATGCCGCC<br>TTGTATTACTCGCAGCGAGCAACCAAAGGCGGCCTCCTCATCACTGAAGGCACAGGTGTTTCCGACACTGCTCAAGGATACCCAGATACACCTGGAATTTGGACAAAGGAGC<br>ATGTGGAGGCATGGAAGCCTATAGTGGAAGCAGTCCATCAAAAGGGTGCTGTTTTCTTCGTGCAGTTGGCGCACGTTGGCCGTGTCTCTACTTATGATTACCAGCCTGGCGGT<br>GAAGCTCCAGTCGCACCAACAGACAAGGCATTGACCCCTGGCTTATACGGAGTAGATTGGTCTCCTCCCCGAAAACTAACCACCGACGAAATCCACGGCATCGTCAACGATT<br>TCAGGCTGGCTGCTCGCAACGCTATCGAAGCAGGATTTGACGGAGTCGAGCTACACGGCGCCAACGGCTTCTTACTGGAGCAGTTCATGAAGGATCAAGTGAACGACAGGAC<br>AGACGAATACGGCGGCAGCCTCGAGAAGAGGTGCCGCTTCGCTCTGGAGGTGGCGGCGGCGGTGAGCGACGAGATAGGATCGCACAGAGTGGGGATGAGGCTGTCTCCCTA<br>CACCGATTTCAACGAGGCGGTGGACTCGGATCCGGACGGCCTCGGGCTCTACATGGCCAACGCGCTCAGCAAGCTCGACCTCGTGTACCTCCACGTCATCGAGCCGCGAGGC<br>GAGGGTGCGACGGCGAGCGAAGGGTGTGGTGAAGATCAGCTCCTCCCCATGAGGAAGGCCTTCAGCAACACCTTCATCGTTGCTGATGGCTTCGACAGGACTAAGGGGAATT<br>GCGCCGTGGATGATGACGCCGCCGATTTGGTGGCGTACGGCCGCCTCTTCCTGGCGAATCCGGATTTGCCGGAGAGGTATCGCTTGGATGCGCCGCTTAACAAGTATGATAGG<br>AACACTTTCTACACTTCTCACCCTGTTGTTGGTTACACTGACTATCCATTTCTTCAACAACCGTAG
SmOPR3-5	Protein	MQLMGTSTSTTSFSILFNSQRESPPLLICKNMGSTAEDKPQIPLLTPYKMGKFQLSHRRKVGSRSGPGSVEFFHIMRFMANAALEVLGRLLFFCWISILLCSWRAIIAAHEIVLAPLTRQR<br>SFNNVPQPHAVLYYSQRANKGGFLVSEATGVSDTAQGYPHTPGIWTKEQVEAWKPIVDAVHAKGAVFFCQIWHVGRVSNTGFQPNGQAPISSTDKELTPQIRANGVDVAQFSPPRR<br>LTTEEIPQIVNDFRLAARNAMEAGFDGIEVHGAHGYLIDQFLKDNVNDRTDQYGGSLVNRCRFAHEIIEAVTAEIGATEYELLYCHMVEPRMKTVGEKSECPHSLVPMRQAFKGTFIV<br>AGGYDREDGNKAVAEDRADLVAFGRLFLANPDLPKRFEVNAPLNKYDRNTFYIPDPVLGYTDYPFLDETA-
	CDS	ATGCAGTTGATGGGCACTTCGACATCCACGACTTCATTTTCGATTCTTTTCAATTCTCAAAGAGAGAGTCCGCCATTATTGATCTGCAAAAACATGGGAAGCACAGCTGAAGA<br>TAAGCCGCAAATCCCACTTCTCACACCATACAAAATGGGAAAATTTCAGCTCTCTCATAGGCGAAAAGTCGGCTCAAGATCAGGCCCGGGCTCTGTTGAGTTCTTTCATATCA<br>TGAGATTCATGGCTAATGCAGCTTTGGAGGTGCTTGGGCGTTTACTCTTTTTCTGCTGGATTTCGATTCTCCTTTGCAGTTGGAGGGCGATCATCGCTGCACACGAAATTGTTCT<br>GGCGCCACTTACGAGGCAGAGGTCATTCAACAATGTACCTCAGCCCCATGCTGTATTGTATTACTCTCAGAGAGCCAACAAAGGGGGTTTTCTCGTGAGCGAAGCAACCGGA<br>GTTTCTGATACTGCACAGGGATACCCTCACACTCCGGGAATCTGGACAAAGGAACAAGTTGAGGCGTGGAAACCTATTGTAGACGCAGTTCATGCCAAGGGTGCGGTCTTCTT<br>TTGCCAGATCTGGCATGTAGGCAGGGTTTCAAACACAGGGTTTCAGCCGAACGGGCAAGCTCCGATATCATCCACGGACAAAGAACTAACCCCGCAGATCCGAGCTAACGGC<br>GTGGATGTTGCGCAATTCTCGCCTCCACGTCGCCTGACAACAGAAGAAATCCCACAGATTGTCAATGACTTCAGACTTGCTGCAAGGAATGCTATGGAAGCTGGTTTTGATGG<br>AATCGAGGTTCACGGTGCTCACGGCTATCTCATAGACCAGTTCTTGAAAGACAACGTGAACGACCGGACAGATCAGTACGGAGGGTCTCTAGTGAACCGGTGTCGTTTTGCTC<br>ATGAGATCATCGAAGCTGTGACAGCCGAGATAGGAGCGACAGAGTACGAGCTTCTTTACTGCCACATGGTCGAGCCGAGGATGAAAACCGTGGGAGAGAAATCCGAGTGCC<br>CGCACAGCCTTGTCCCGATGAGACAGGCATTTAAGGGGACCTTCATTGTCGCGGGTGGCTACGATAGGGAAGACGGGAACAAGGCCGTGGCCGAGGATCGTGCTGATCTGGT<br>CGCTTTTGGCCGGCTGTTCTTGGCTAATCCTGACCTGCCTAAGAGGTTTGAGGTGAATGCTCCTTTAAACAAATATGATAGGAATACTTTCTACATACCTGATCCTGTTTTAGG<br>CTATACCGACTACCCATTTCTGGATGAAACTGCTTGA

Table S5. Proteins used in the phylogenetic tree of JA gene family

Protein name	Plant Species	Accession number
AtLOX1	Arabidopsis thaliana	NP_175900.1
AtLOX2	Arabidopsis thaliana	NP_566875.1
AtLOX3	Arabidopsis thaliana	NP_564021.1
AtLOX4	Arabidopsis thaliana	NP_177396.1
AtLOX5	Arabidopsis thaliana	NP_188879.2
AtLOX6	Arabidopsis thaliana	NP_176923.1
CaLOX	Corylus avellana	CAD10740.1
CfLOX	Camellia fraterna	QCQ29247.1
EcLOX	Eleusine coracana	AHH24969.2
FrLOX	Fragaria x ananassa	CAE17327.1
GmLOX	Glycine max	NP_001235189.1
HbLOX	Hevea brasiliensis	AWR88469.1
MuLOX	Macrotyloma uniflorum	AIL90390.1
OsLOX	Oryza sativa	CAC01439.1
PaLOX	Persea americana	AAD09861.1
PdLOX	Prunus dulcis	CAB94852.1
SlLOX	Solanum lycopersicum	NP_001233812.2
StLOX	Solanum tuberosum	CAA64766.1
TuLOX1	Triticum urartu	EMS65931.1
TuLOX2	Triticum urartu	EMS61461.1
VrLOX	Vigna radiata	AGS94394.3
VvLOX	Vitis vinifera	NP_001290017.1
AaAOS	Artemisia annua	ADZ24000.1
AtAOS	Arabidopsis thaliana	NP_199079.1
BoAOS	Brassica oleracea	AGB34186.1
CfAOS	Cymbidium faberi	ASA69245.1
CisAOS	Citrus sinensis	NP_001275835.1
CusAOS	Cucumis sativus	NP_001274390.1
GhAOS	Gladiolus hybrid cultivar	AEO13838.1
GmAOS	Glycine max	NP_001236445.1
GmAOS1	Glycine max	KAH1252408.1
GmAOS3	Glycine max	KAH1242324.1
HvAOS	Hordeum vulgare subsp. vulgare	CAB86384.1
InAOS	Ipomoea nil	BAK52267.1
KfAOS	Klebsormidium flaccidum	BAS32649.1
LuAOS	Linum usitatissimum	AAA03353.1
MeAOS	Manihot esculenta	XP_021613093.1
MpAOS	Marchantia polymorpha	BAS32648.1
MtAOS	Medicago truncatula	CAC86897.1
NiAOS	Nicotiana attenuata	CAC82911.1
OsAOS	Oryza sativa	AAL17675.1
PsAOS	Pisum sativum	BAE45341.1

**Protein name**	**Plant Species**	**Accession number**
RcAOS	Rosa chinensis	XP_024167128.1
SlAOS	Solanum lycopersicum	NP_001234833.2
SlAOS2	Solanum lycopersicum	NP_001274707.1
SlAOS3	Solanum lycopersicum	NP_001265949.1
StAOS	Solanum tuberosum	CAI30876.1
TaAOS	Triticum aestivum	AAO43440.1
VaAOS	Vigna angularis	KAG2401435.1
ZmAOS1	Zea mays	XP_008644470.1
ZmAOS2	Zea mays	PWZ56648.1
ZmAOS3	Zea mays	XP_008679839.1
ZmAOS4	Zea mays	PWZ28798.1
AtAOC1	Arabidopsis thaliana	NP_189204.1
AtAOC2	Arabidopsis thaliana	NP_566776.1
AtAOC3	Arabidopsis thaliana	NP_566777.1
AtAOC4	Arabidopsis thaliana	NP_172786.1
HlAOCC1	Humulus lupulus	AAT96851.1
HlAOCC4	Humulus lupulus	AAT96852.1
HlAOC-P	Humulus lupulus	AAT66741.1
HvAOC	Hordeum vulgare subsp. vulgare	CAC83766.1
IiAOC	Ipomoea nil	XP_019173088.1
MtAOC	Medicago truncatula	XP_003614458.1
NtAOC	Nicotiana tabacum	CAC83765.1
OsAOC	Oryza sativa Japonica Group	AAR89017.1
PpAOC	Physcomitrium patens	XP_024374815.1
PsAOC	Pisum sativum	BAE45342.1
SlAOC	Solanum lycopersicum	CAB95731.1
StAOC	Solanum tuberosum	NP_001275212.1
VvAOC	Vitis vinifera	NP_001267900.1
ZmAOC1	Zea mays	NP_001105245.1
ZmAOC2	Zea mays	NP_001141267.1
ZmAOC3	Zea mays	AQL06179.1
ZmAOC4	Zea mays	ACG32920.1
AtOPR3	Arabidopsis thaliana	NP_001077884.1
GaOPR3	Gossypium arboreum	KHG19112.1
GhOPR3	*Gossypium hirsutum* (cotton)	NP_001313917.1
GmOPR3	Glycine max(soybean)	XP_003551126.1
InOPR3	Ipomoea nil (Japanese morning glory)	XP_019189633.1
LrOPR3	Lilium regale	ART33470.1
MnOPR3	Morus notabilis	EXB38932.1
MtOPR3	Medicago truncatula (barrel medic)	XP_013458417.1
NtOPR3	*Nicotiana attenuata*	XP_019256011.1
OsOPR3	Oryza sativa Japonica Group (Japanese rice)	XP_015643918.1
PsOPR3	Pisum sativum	BAD12186.1

Protein name	Plant Species	Accession number
SlOPR3	Solanum lycopersicum (tomato)	NP_001233873.1
VvOPR3	Vitis vinifera (wine grape)	NP_001267975
ZmOPR3	Zea mays	NP_001105830.1

Table S6. Promoter sequence of JA gene family

Gene	front 1000bp sequence
SmLOX1	TTATCCTAAAATGTGTTTCTTTCTTCATGCTCGATTACACTTTTTCAAAATCGGTTCTCTTTTAAGGTTGTTTTTATAGAATGAAAGTATCATTACAACTTTCAAGATT<br>GTCCTATTATTTCTTTGTACGACTTTGCAAGCCATTAGAGACAAATAATTTATCGGCTTCATATTTAAATAAGACTTACAAGTCACTAGAGACCAATACTTTTAGAG<br>GTCAAACACTACTCAAACATATAAGTTTGGCCCAAATGAAACGAGCAGCCACACATACCACACGTTGCTTATCAATTTGTTAAAGGTTGGTATTTTTTTCAGAACGA<br>TTATTATTTTTATTCACAAGAACTTATACTTTTAATTTATTTAGTCAAATTACTTATCAATTTGTTAAAGGTTGGTATTTTTTTTTCAGAACGATTACTATTTTTATTCA<br>TGAGAACTTATACTTTTAATTTATTTAGTCAAATAATGATTATCGTAAAAAAAAAGGTAACAATTGATATAAACAAAAGTCATATTGTCCCATATATATATTATTTT<br>GCTCCTAAATCAAACCCGGATTAAGACAATAGTGAAGAACGTACCACTGTAAACACGTTTTATTTGTAAAACGTGAATATTTTTATTTATATCTCGGTAAAACCGGC<br>TATGTAATAGATGAGATCGGTCTCACCCAAGTTTTTGCGTTGATATATCTGTGGTAAAGAGAAGAATATAATCACATTAAAGAAAAGTAGCCCATAAGCAATTGTA<br>GAGAATGAAAGCTGCATAAAGTTGAAGGAAGACTCCTCTCAAAAAAAAAAAGTTGAAGGAAGACCAGTAGCTAGCTACATATGGAAATGAAACTAGAGAGTTAC<br>TATTAACTACATTTGATAATGATGAGTGTATGAGCTAGAAATTGAAAACGTGGTCGCGCCTCTATAAATACATCCACCCTCCATAGTCGTCTCCATTCATTCATACG<br>TTCAATTTAGTCATTTAGAGAGAGAGAGAGAGAGAG
SmLOX2	AGCTTTGCGTATCGATATCATTTTTTTTAATATGATATCATATTTTATTCGATTAATGTATATAAGGTATTGTGCAATTTGAATTTGAGATATTTAAGAAAAAATATT<br>CTACGACTAAACCTATCCCAATAGGACATAGTATAATTTAGAATTAATAGTGTTTTATGTCCCGAAATTTCAACGTTTTTCATAAAATATTCTGAAATTTAATTTTCT<br>CCTAAAAAACTCAGAACTTCCAGCTTTTTCTATAAAATGTCTTGCTATACAAAATTCAGTGACGGAAAGATGACTTATCGTGCCGGATTAAATATTTTTGGAACCAT<br>CCATATTAGAAATCACTACTGTTGAAAAACACTGAAAGTTCGGGGCTTTTTGTCATTTTTTCATCACCAGATTTTGTATAACAGGATATTATATGGAAAATGTGGAA<br>ACTTCGGAGTTATTTACGAGGAAATAAAAATTTGAGACATTTTATGTAAAACGTTGAAAGTTCGGGACATAAAATAAAACACTGTTAACCTTATAATTTATAAACA<br>CCATTCTTGTGAATATTGGTAAGATAATATACAAACATATAATCCTAAAAAATAATTTCTAACAAGAAGTTGGGTAGACTAAAGAACTATACACTTTACAATCTTAT<br>ACCTACCCAGATCAGCACACTCCAATGGCAACGACCAGGCTGGAAATTACTAATCAATTTCCACTTCACAAAGACAAAAATCATTCAAATTATTATATTTCCCACTT<br>TCAAAAAATGTCATGTTTCCTATTTGATTACCCACGTGAAAACAGCATGTAATATTAATTAATTCTGAAAAAGGCCCAAAGCATATATCAGACGACTCATCTACCTT<br>CCAAGAAAACACACCATAAATGTTGCTACAGACTACAGTTGCATTCTTTCTATAATAAAACCACCCATTTTTCCCAATCAAACTCATTGCATCTTCATTTCAAAAAA<br>AAAAAAAAAAGTTTGTGACACAATTTTCATCTCAAA
SmLOX3	AGGAATGGAGTAATTCATAATTTATGGGACCAAAAAGGGAGATTATATATAAGATATTGTCTTTGAAAAATGAACTAGTCCAATAGAGTGGTTACACGTGTCCTAG<br>AGATAAAAAAAAGTTACAAAAAAAATTATAAACAAATAATACAATGTACATAATACAATAAAATAACTAAATCTTATTTTTATTTTATGAAATAAGTTAAATAAAT<br>TAATCTTCTTTCTATTTAAGTGATTTGTGAATGACCACAATTTGACTATTTAGCATCACTCAGGCAAAAAAAAGTGAAATCAAGCAAATCTAAAAAAAATAAAAAG<br>GAAAAAAGAAAGAGGTAAAAAAAAAGTGAAATCAAGGAAATCTCAAACTATATACTCTCTCCGATCCCACGAATCTTGACACATTTCCAAATAAGGTAATAATTA<br>TTACATTCTCTCTTCTACTGTATCACTTTTATTACCTTTTCTCTCATACTTTATCACAGTACTTTATTAACTACACACTTAAAACATCAATCTACAACTCCTTAATTCAG<br>GTGCCGAAACCAAATGTGTCAAGATTCGTGGGATGGAAGGAGTATATATATATATATATATAAAAAAGGAAAAAAGAAGAGGGTTGAAGAAGAGAACATGGCCA<br>ATGTTAAAAAAGAAAAAGAAGAAATTCAAATAAAAGAAAAAAAGAAATTCAACCCACGAGGAGAAAAGTGGGTAGGCTCAATAATCAAAGCTATAATAACATCC<br>AATCCAAGTTATCCAACAGCAAACAGCAGCTGCTTCTCCACACACACAGATTTCCCCACTTTTTCTCACATTCTACTATCATAATACATATACCCACTAATCTAAAC<br>ACGTAATTAGCATTAATTACATCGAATCTTCTCACACTTTACTTAACCCCCCCCCATCATAATCACCCACTCAAGCAAGGCATAATTTCCAACTGAAAATCCCCCAT<br>CGTCAACTAATTTCTTCCTATTCCACTTTCCCCCCCTTTTTCGCA

Gene	front 1000bp sequence
SmLOX4	AATACTACCAAAGCAAGGAAACTGCTTCAGCGCAAGAGGGAGATAGATATGTAAAACTCATTTTTAGATCATTTAAAAGCTTGTTCCACAAAAATAAAAAGTAAA<br>ATCGGTGATGCTATTTGGATCATTTTGTTCCGGATCAGATACAATAAATATTTCATGGAGAAAGAAAAATCATTAACTATTTATATTTTTAATAATTTTGACTTAATA<br>TATATATATATATTAATTGATTAATAATTCATCGTTAAATTTGCTTTTAAAAATCATCATTAAGTGGATATTTTTATTTTTCAAAAATGATAATATATTTTAATTAAAA<br>TATAGTATAATTTTTCATTCAATTTTAGAAATATGTTAAGTTATTTAAATAAATTTATAATATATATAATTCAAATTTCGAACTTAATTCAATATATATATATAAAACTT<br>ATAAACTTTTACTACGAGTAAACACATTTTCAATTGTCCCCAAGGAGACACCAAAGGTGCGAACTCTTATCTACGAACAGTGAGGTGCGGGGTTCAAACCCTATCG<br>GCTACCGCTGCCCTCCCAAAAAGTAAAAAAATAAAAATAAAACATATTTTCAATTTTAATATTACAACCTTTGTCCTTGTTGATCCAGATCAATTTGATTTAAATAA<br>ATTCATCCAAACAAATCAAAGAATGAAAAAAAAAAATTCAAATCTGGCCCACTTATTCAAAAGAAAAAGAAAAGAAAAAGTTGTTAGAATATTGTCTGCTTATGCC<br>GATTCGTTTTCCAATCACAGTGATACTGATAATATATTCCCTTCCCAGACACAAACAGTGTTTTCCAACTCCACTTACATGGAATTTGCTACGCTCAAAATCACACTC<br>CCCATCAAATTTAAATATTACCTTTCACACTTTGGAATTTTCCTTGAAATAATACAAGTCAACCTATACTAAAACTAGCACACTGTTTTCTACTAATATAAGTATCTT<br>CTCCCCTTCATCACTCTCACTCCACAATTTGCAGCG
SmLOX5	TTTCATCTTGACCACAGCACCCAACAATTTATTGAAAAGAATATCAAAAGGGAGCGGCCGCGTTCTCATCATCTCCCGGAATAGAGCGACGGCATCATCGGGCTCA<br>CGTATAGATCCAAAATCGAATCTGGGATAGGCGGAGAAGCGTCTCTTCATTTGGCAGCTGGCATTATCAAAATGAAAACTGGGTAGAAGAAATGATTGATAATGTG<br>GAGAGGCGATGGCAAAAGTGGAGAATTTAGATGAAGAATTCGACAAAATTCTAGCCAGAAACATGGCGGCGTGGTTCGACCTCATTCTTGGGATAGTTTTGTAGG<br>ATTTCAGATGTGAAAGCTGAATTTGGAAGGTGAAGAATTGGGGAAGGAGGAGAGTTAGGGTTTTGTGAGAAGAAAGTTGAAAAATGAGTTTTGGGGAATTCGCAG<br>TGCTAGGTTAGAAAGAAGGGCAAAATAGATCCTCATTATACTTTGCTAAATTTAACTTCGTTTTTAAAAGAAAAATTTCTATTCTCAAAAAAAAAAAAAAAAAAAA<br>AAACTTATTTTCACTTTCTAGTTCATGACGAGTCTTCCACAAGAATTATGTTTAACGAGCATGATATGAATTGTGCATATTATTCATAATTCGAGTTTCTACAATAAA<br>AAATTATGAAACCATTACAATTCATTAATTATCAATTATAGTTTATTAATCAAAATTAAAATTCAACTATGAATGTATCGTATATCTAGATGCACTGGGGTTGTACCT<br>CTTTAATTTGTTATGCCTCAAATTAACAAGAGCTTCATCACGTAAATGATATTTTGTAAATTTTGAATTACCAAGCACATTTCCAAAATAAAGACATCGCAATAATA<br>ATATATTACCACTGCAATTCAGTAAAACCTATACATTTACAACTCGAGAAGCTAAAAATGGAAGTCCAAAGAAGTGAATGAATCCGATCCTTTCCTCAAATCGCAA<br>GATTTGCACACACTTCCTTTCTTGAACATGATACTAAATATCA
SmLOX6	TAAATAAAGCATTGTATATAGAAGATTAGTAGCATTAATTCTCCAAGAATATCTATTTGAATTCGTTGATCCATTAACTTTTGATCTTCAATCCGTTGGAAGCTTTTT<br>GCAATAACCACACAACAAAATCACACTTTCAAATCCTCAGCACCTAAATTAGTCGAAATCGATCTTTTAATTATTTGGCCAATTATCGTGGGGGACACACTTTTGAT<br>AGGGATGTTGGGGGATGTGGATATTGGATTAATAATAAAAAAAATAAAAATATTTTAATCTCTAATTACACTCACTAATTGAACCCCTTATTTTTCTTAATCAGCCC<br>AATTAATTATTTTGGTTCTCCCTCCCATATCCATTACGCACATTGTTTCTCTGGCCCTCCAAATTTGTATCACTTGTATATGTACTGACGCTTGATTAAATATTGCGCT<br>ATGTCAATTTCATAAGTGAGCAAGTTGTTGCATTTCAAATTTCAATACAATATGGTAAAAATGGTTTGGTCGATTTTGTTGCATTTGTCAATTGTCTCATGATTACAT<br>TATCCTGCACGCCTTCCTCGTCTTTTCATTTTAGTCTTTACATCATAAATTACACAAATATCTATCCCATATTACCCAACATCACAAAATTTATCAGCAATATACTCCA<br>CCCATATTCAAATTTTAACTCAAATATTTTTAAATTTATCAATTCAAACTTAATTTTATTGCCAGTAGGTGTAAGTATGAATTGGTGAACACAAGTTTTGTAATATCT<br>CGAGCGTTAACAATCATATTACTCTGTTGTGTGATGTTTGTCGGTATCTCGATCTCAGTCCATGCCTACAACCCATGCCATGCCCACGCAATCACACCAAAATAAAA<br>TAAAAAATGACTTCCAACTACAAATAAATACTAATTAAATATGACTCGTGAAAACAATAGTTTACTTTCTTCAAAGAAATGTCAATATAATTAGTCCCCATGCTCTA<br>CACATTTTCCACTTCATTCCCTGCAGAAAG
SmLOX7	TTATCAATAAATTAATAATTATTAATTTATTGATATATTAATATATATCGATGTAATTTAAACTCTCTAAATTAATAAAATCATTGGGTCATAAAATTATAATTTATA<br>GAGGTTTTACTGTATTTTTTAAGAAATTTAGCATTCATGCTAAATTTTTAAACACATACATATCAAAGTATGATAACATTCATATGCTCCATCCGTCCCATTTATAAC<br>GTCCCAATAAATTTTCTTTTTGGGACGTCCCATTTATAATGTCTCAACCTAAAAAAAGATATAAATTACTTTATTATTAGTCTATCCCTCTACATTTACTTTATCTCTC<br>TCTCTCTCTCTCTCTTATCTTTAACTACTTTTTCTATCTTTATTCGTGATTTACTTTATCTATATTTTTTCTTAATTTGTGCGCCCCATCTTTTTGGGACGTTATAATCAA<br>GACACAAATAGTACAATTTAGTGTATGTGTGACATGTACCTTATTTATTTATGTAATAGTGAGGACGTTATTGTTATTTTCATTTATCTAGTTAAGCAGTTAGATTCG<br>ACCTATAGGATATTTATCTTTTTCAGGATCCTAAAAAGAGTTTGAGATATATATCAAATAAATTTAAATAAGGTAATCTAATTTATTAATATATTTTTGTAGTATTTT<br>AATATCTAATAAAATTTTCAGCGCATTTTCAAATGGTTGGGCGGTTGTCTAACAGACTCCTTTCTTTTTAGGCAGGTGTCCTAAGATGAGCTCAACGAGAATATCTA<br>TTAAATTAATGTATTGTTCTAAATATATAAAATATAATAAACATAATTCTCCTTAAACTTAAATATATAGTAATAGGGATAGTATATATATATATACACACACATAT<br>ATATGGGACTAATAAAATTTCGGGGCAGTAATTTTCTGGGAATCTGTGGGGTCGCATAGGCCCAAAACCGGTCCTGCATGAATATGGTCAACACTCACCTTTGTGC<br>GTGTACTGTTTGGTGTTATAATTACTAC

Gene	front 1000bp sequence
SmLOX8	TTTGTGAAGAAGCCTCTATCATCAAACTAAATTATTAAAAAATTATCTTTTAAAATTTAGAACTCAAAATTTAATATATGTTGTGTCTATTAAGGATTTTCTGCATCC GCCCCTACCCACAATGCAATGACTAAATATATATATATAAAAATCCAATGAAAAAATAGAAGTACAAAACATCTGCTCCATCCGTCTCAATTCAGTAAGATCAATT TTTTTTATTTGGATGTCACATTTTAAAAGGCCGTTTCTTAACTAAATGAAAAATCAAAACATAACAAACACTCCCACATAATTTATTATTTACACTAAATATTATTAT GATTCACAAATAATTATCTCTCTTTTAATTTAATAAAAAATATCTTCATTTATCTCTTCTTCTTGTTCTTATTTTTACAAATAATTCTTAAAAAAGAGTTCATTTCTTTA TCATATTTTATTAAAAATTAGTACTCCATTTTGTTCTTAACATATGTGCACAGTATTATAACCTATTGAATTAGGAAAGAAATGAAAAAATGCACTCCATTATTAATA AAAGAAAAGAGGAAATACTCATGCAAACCTTTAGATCCACTAGGGACACAACCGCCATTTTAGGCTACACAGAAGGACAAAATGGTCCACCCAGCCAAAAAAGCG AAACGCACTTACCAAATAGCAGCCTTTCACAATTGATATCATAGTATTATTTGTGTTTGCATGAAAATTCTACGAAAATTCGAAATCTCATTTAACAAAATAAAACT TATTTTTTATTATTTATTTTCTGCATGTGGTCACATTTTAGAAAATTGAAAACGTGTGGCCGGTGAGGTCTTATATTTTCTCCTCTTTCACCATCGCTTCCCCCACGCT GTATCTATACATCTGTGTATATATACACGCATAAATGCAGAGAGAGAGAGAGACAAACACCACCATTATTTCTGCTTAATCAAAGCTCCAATTCTCGATCACACAA AAGGGTCACTCGAACTAGCGGAAAGATTCAAGC
SmLOX9	TTGTCTCCTCATGTGAAGACTTACAATATCTTGTTGGATGGTCTGTGTAATGCTCATCGTATCTCTGAAGCATTTTCCATGTTGCATACCATGAAAGATAGAGGAGTC CTTCCAAACATAGTAACATACAATGTTCTCATTGAGGGATTGTGCATTAATAATAAAGTCAGAGAAGCAAAAGATCTGTTCGACGAGCTTCCATCCAAAGGTATGC AGCCCAATGTCGTAACATATACAATCCTTATCGGTGCACTTTGTGAAGAAGGACAGATAGAGGAGGCTAAGGATTTGATGATGCAAATGGTAAACAATGGTTGCTT GCCTGATAGTGTGACGTACAATGTTTTTGTTCAAGGTCTTCTCAAAAGGAACAAGACGTGTGATGCAATTCCACTGTTGGAAGAGATGGATGCAAGGGGTTTCACA CTTGACGAAACTACTTTTTCGATGTTGATTGAACCTGTGGTAAGGGAAGGTAGAGATAGTGTTCTATTTGATAAGATTAAGAAACTCGTACCAAAGGACCTCTATTC TAGATAGGTGTTATGTTGTGTTAGAAGTTGAAGAATTATTTTACCGGAAAGTAAGGGGAAAGTACAAATGCTTATTTGTTTGTTTAATCTCTATGTTATGATGATTTT AAATGCTTGAATTTTATAGCTCTATCATAGATTACTAGGATTTCCAAAGGATATCGCTCGCTCTTGGCAAAGTAGTATTCATTCTAGGAGTAGAGCTTATAAGATAA GATGAATAGTGCCATAATTCATCAATTTGACACTTTGAATTTCACATGATTCGAAATACTGCAAAGTTGATTTTGAACTCTTTAGAAAAACATAGGGAATTTAGCTT TTACGTATTTGTCTTTTAGGTGTAAATCGAAGTATAAGATAATCAAAGTTTTGTTTCCAAATTGAAGCTTTTATATTGTGTTATAGTTTGTCTTTGTTTCAATGAGGTC GAACCATGCCACCATGTTTGTGGGAAGAGATATTGA
SmAOS1	TTAAACGTTTTTATACTAATCTCATACATAATATTCAAAGTGAGTGGCAGCGGTGCACCAACTTAAATACTCATTTATAATGTGTAGATAGCAACATGCCAACATGC ATGTGGCAGCACAAAGCGAGCTATAGTGGAATCAATTAATTAATGCACATCTTCCCCATAGATTTTGGTAATCCACCCCATTCCATCATCATTTCTATTATTTTATAA ATGGATTATAAAAATAAAAAAGTATTAGTATAAAGATCTTTATTTAATATTTACGTACTGTTAAAAATCAATTATTAATGAAAATTTACAAAACAAATATGAAAAA TCACTATAATTAAATCATAATAAATTAATGAACATCATTAATAATCTTAAACCTAAGAAATAAAGAAATACTATAATTAAAGAAAAAATAGTTAAAATTAGGAAAA AAATTGAAAGTAGGTAGAATTATAATTAAGTCACTTGATGAGGTTCAACCTAGTCAATTTGGTGTCCTACATTATGTATATTCCATTAAGATAATTTATTTATCAATT TGTTTAATTTTGTTTATTGAATGCGTATTGATGAATTTCTAAAGATTTAAAATTGTAAAAAATTAAAATGTATCTAAAATTATTAAATATACTTTTTCTTTATATAAAT TTTAAAGTAGTAATATGATCATATATCAATTATTTATAAGTATATAAAAATATCACATTAAAAATAATAAAGGATAGTATTATGTTCACCTTCAATTTTATTTATCGC TTGAGATCCATGTGGTCAAACTCTCCATATACAGTTATATAATAATAAAGTGAAACCAGAAGTGAAACCTAGCTAATGGGCCATCTTCTCCACTTAATAATTCTTCC TAAATTATTCATTCATTAATTCTCTCTCTCATATAGATATCATATGCTCTCCCACCAATTCTCTCTCATATATATCATATGCTCTCCCACCATACTTGCACAATTATTC ATCACCAATTTTGTACTAATCAACACTACACC
SmAOS2	TTCAATTTTCTGTTTATAAGAGTCTTCCTTATATCTCTGTGGCTTCGCAGTAGTCGTCGGAGACAAGGGAGAGGGGCGAAGTTGAGAAATGGTAGAGACAAAGGGA GAACAGGGGGCTGGGACTGGCAGTGGCTGCTCCCGCTGCTGCTCGGCGAAATCAGAGAGGTGGCTCCGCCTACTGTTGTCGTCGCCGGAGTTGAACATGGTGTCGC CGGCGGCGCTGCGACTGCATAGTGTATGTTGAGGGAGAGAGTGCTGAGAGACAGAGACATGTTTGGGGAGGGCTTAAGCTATACTTTACTTTTTCTTAAACATGTG GGACCTTCTATTTTACACCACTAATTTTACACTCCTTAATCTCCGTGCCCAAAAGAAAGGGGACAACATTATTGGGATGGAGGGAGTACCTTTTTATCCACTCTCAA TACACTCAACAATATTTTTTCTTAAAACCCGTGCCACTCTTTCTAGTTCTTTCATGGACGGATGAAGTATAAACTTAAAACTGAAAAAAAATTAAAAATAAAACACG AGATGAGATATAATTTATAAAATACGGTGCACTGGATTTCATTCTATATCGAGATTGGCCATGAAAGATCCGCAACTTTAGCATTCCAAAGTAGCTACAGTTGTTTA AGGTCGGTCACCAATTGATTCTAGTACATCAAGTTACTTTTCAATTGTATGTCCAAAATAAAAATAAAAAGTTACTCCTTTTTATTTGTGACATTTCTAGGAAAGCG GAATTCACCATACTTTTCTTCATTCGTAACTTTCCTACTATTCAATATTGTATTGGTAGCATTGGACGGCGCCGGTAATAGAGTAGAAATTTTCCGACCCATGTTTTT TATACTTGATCGCAAAATCACATTAATAATTCTTTTTTATTTTTATTTTATTATGCCCTCCCATGTTTATTTATACCTCCAGAATAGTCAACTAAATCAATCAAACTCA TTTCGTAGCAGTCAAAATCCTCATAACTCACTCGGCA

Gene	front 1000bp sequence
SmAOS3	CATAATACAAGCTATCATGCACTTGCATGCATAGTATAATCACACTCGAGCCATTAATTGTTTTCAATTAAACATTTCTCAAACTCGAACCCATTAAACATTTGGGG<br>GTTTATAACTTTATACAAACAGTGAACTTTCTCTAAATCTGTGTTAAGAAAAAGGTCTAGGATTGAATTCTCACGGTCTTCTTATCCTAATTCAAAAACATATATAAC<br>CGACATTATCACTATAGAAAATAAAGCTACAAGAATTTATTGACAACTTACCTCTTCAAAATGGAAAATCAAACTATTTTTTTTCATATATAGTTTCATTTTTTTTA<br>CTCTGCACCCCTCATATATATCAATTCGTTAACAAAAATCCTGTATACACATTTAATTAAATACTACTACTATTTACTTTTTAACAGTCCCCCTTCAAACCCTCTCAA<br>GAAAAGAAAGAAGAAGAAAGAAACCCCTTCAATAACTAAGAAACAAGTGGACATGCATGCTTACTTTAAAAGTTTTTTTTTTCCAGAAAATTCAAACTTTAAAAG<br>TTAAGAGACTAATAATTTCCTTATATTTATATCCATAACCTTTCAACGAGATCTTCATTGAAATTTTTAACCATATATCCACGCTTCACATTAATTGATTGTTAATTTA<br>GATATACTTATAAATCAAACTTAATTACTTTTCCGCGCATTGACTGTAGATACCTACAGTTTGCATTGCACGTCAGTACTCATCAAAATGAGTTTGACGCCACGTAA<br>AATTCATAGTCTACTACCGCGCCTAACTCTATTTGTCCTGCATGCCTAACGCACGTGGATTTTGGTAATTTTGAATTCATCAATCCAAATTCCAATTCTCAACTTTTTT<br>TTTAAAAAAAATTTAATCACGAATCCATCTCAATTGGCCTCTCATAATCATCTCTATATATAGGTCCAATTTGATTAACCATAACATAGCAAGCCACAATTTCTTTCC<br>ACATTCTTCAAAACTCAAAAAACAAGAGAA
SmAOS4	AAACCCCCTCATTTTTGTGGATGATTTCTGCCATCGCCGGAAGTCTGATTTGGGGATGATTTCAGGCGGCTGGTTCGACGGCAGATAGAGGACGGCGAGGGCCGAC<br>CGCCAGCGCCACTGTCACCGTCGCCCAAATGCAGTGAATGCAGTGAGAGGAAGAAGTAGTTTTAATTAAATCCTTAATTTTGGTGGACCACAATTAATTAAACATA<br>ACTATCTCCTTCTTAATCTCCGTGCCCAAAAAAAAAGTGGCCACTTTTATTGGGACGGAGGGAGTAAATGGTATGTGAGATGAGCTTTTTGTAAATATTGTGTGAGAA<br>TGATGTAAAAGTATAAAAGTATATGTCCTAAAATAGAAAGACCACGCTTATTGTGAACGGACGAAAATGACAAAAAAATCACACTTATCGAGAACAGAGAGAGTA<br>TAACTTAAGCCAGTAGTCCCCAATGGATATTCAAGCAATGCGGGCTCGTTTTTTTGTAAACAGCAAAGTCAAAGGGTTCAAATCCACTACTCCCTCTGCGTATAATT<br>AATCACCTCTATATATAATTCTATAATTAATCATGCAAAATAAACTTTTCCCTCTAATCCACAATTATTAATTTAACACGTCCAGACAATTAAATATTCATAATTATT<br>CGCTTCCCCAATAATATTCAGATCGATCGACTAACTAAATACAATCTCAATAAAATTAATCATACACCCCACGCAAAAAAAAACAGTTGTACTTTAAGTCGAAAAC<br>AAATACAGCAATCTCGATGTGTCGGGTTAATAATGAATAAAAAGATATGGTTGTCAAATCAAATTATTAATTAATTGGGGATGGACCTTGCATTAAACATTTTATTT<br>GTTCAACAAAGAGCAGTGCCCCATCCTCCCCCTTGGCCTTCCATAATGTGACTGACAAACCTCAGATTATATATATCTCCTCAATTCTAGATTTCTTAATTATTCAAT<br>CCCCTCCCAATTAACCAAACAAATCCCTAATCTACCGACA
SmAOS5	TCCATAATACAAGCTATCATGCACTTGCATGCATAGTATAATCACACTCGAGCCATTAATGTTTTCAATTAAACATTTCTCAAACTCGAACCCATTAAACATTTGGG<br>GGTTTATAACTTTATACAAACAGTGAACTTTCTCTAAATCTGTGTTAAGAAAAAGGTCTAGGATTGAATTCTCACGGTCTTCTTATCCTAATTCAAAAACATATATA<br>ACCGACATTATCACTATAGAAAATAAAGCTACAAGAATTTATTGACAACTTACCTCTTCAAAATGGAAAATCAAACTATTTTTTTTCATATATAGTTTCATTTTTTTT<br>TACTCTGCACCCCTCATATATATCAATTCGTTAACAAAAATCCTGTATACACATTTAATTAAATACTACTACTATTTACTTTTTAACAGTACCCCCTTCAAACCCTAC<br>TACAAGAAAAGAAAGAAGAAGAAAGAAACCCCTTCAATAACTAAGAAACAAGTGGACATGCATGCTTACTTTAAAAGTTTTTTTTTTCCTAGAAAATTCAAACTTT<br>AAAAGTTAAGAGACTAATAATTTCCTTATATTTATATCCATAACCTTTCAACGAGATCTTCATTGAAATTTTTAACCATATATCCACGCTTCACATTAATTGATTGTT<br>AATTTAGATATACTTATAAATCAAACTTAATTACTTTTCCGCGCATTGACTGTAGATACCTACAGTTTGCACGTCAGTACTCATCAAAATGAGTTTGACGCCACGTA<br>AAATTCATAGTCTACTACCGCGCCTAACTCTATTTGTCCTGCATGCCTAACGCACGTGGATTTTGGTAATTTTGAATTCATCAATCCAAATTCCAATTCTCACACTTT<br>TTTTTTAAAAAAAAATTTATCACGAATCCATCTCAATTGGCCTCTCATAATCATCTCTATATATAGGTCCAATTTGATTAACCATAACATAGCAAGCCACAATTTCTT<br>TCCACATTCTTCAAAACTCAAAAAACAAGAGAA
SmAOS6	TAAGACAATGAAAGATTCTCATTTGCAAGAGTTTTGGGTTCAATTATGCCGCATGCGGATAACATTTTTTTCTAATTCATTCTGAGTGCCCAAAAGATATATGTCAT<br>AAATAATGGCAAGAAAAGAGTATTATTTGAATATTATCATCCAAAAATTATTCAAGTTGTTTTTTTTTTACTTTTTTTTCTGACCAAAATAGTTTTCTAAATATGGCA<br>ATGTATGTCAACAGTCTACATAATGTTCCTGAATAAAAAAAAAAAAAAAAGTGGCCCATTTTATTATATGTATACATGATACATCTAGGGATGTCAATGCAGCCCGA<br>AATCCGTGGGCCGACCCGAATAACCCGACAAAATTAGAGGGTTAGGGTTGAAAAATTGCAACCCGAAAAAACCTCCAGCCCGATTAGCCCGCATCCGACTAACCC<br>GGAACCCGATAGGGCTAGCCCGAAAACTCGATGGGCTGGCCCGGTGGGCTGACAGAATTGTGACTGATGCATCCAGTTACAACCTCTGCTATGTTTAGATCTATGG<br>TATTTGCTTAAATATATATATGTAGCATCATTAAAAAAATGAAATTAATTAGTTAGAATATATATGTGTGTGTGTGTGTGCAATCTCATTAAAAAAAGTGAAATTAA<br>TTACGGATTTTTTGTAGAAATTGATTATTAGTATCTCATTAGTTAGAAATTAATTATTAGTATCTCATTTTAAAGTGATACCATTTTTTTTTTTATCAATGAGACGTGC<br>ATGGCAAATCCACTAATAATTCAGTAATAATCCAGATTGATTCTAGTGCATTGATACATGTTAAATTTTACTATCTCATATATAATATTGTTTATGTAATCTTGAATA<br>TCTCATATTTTTGCATTTCATGCTTTGTTATATAATTTATATCTTTAATATCTATGTATATTTTATACATTATGCATTAGATCATTAGGAATAATAAAATACAAATGAT<br>AATTTTATTTTTACGTCTTTAACCTGATTAGCCCG

Gene	front 1000bp sequence
SmAOS7	TTTATTTACTTTATACACAATTTCTTTTTTATTTATCACAAAAAATTGTTTATTTATTTATCAAACTATTTGACAAAAGACTATCGCCCAATTTTATTTATTTATTTACT<br>TGTGATGTGAACCTACTGTGGTCCAAATTTGCACGTCTCACGTTTACCCTTTTTTAATTTTTTGGTTCTGTTCTTCCTTTTGTAGAAATGTGATACTACTAAAGGTTTT<br>TTGTTAGAATCAAAATAAATATTCGTTGACTAAGAAAAGAGGAAATCGATACGTAGTTCAAGTGTGGCAACAATTAATATTATTTTTTTTTTGAGGAAAAACAATTA<br>ATAATTATAATAAGGCTTATTGGCGATAAAATATAATGCTAACTTCAAAATTAATTAGTGCGAAAATACACTAATTTAACGGTTGTTATAATTTTTATCATGATTAA<br>GATTTTCATCCAAATAAAATCAGTTTGATGTCATAAATCGCGTAAGTTGGTTTCGTCGTGACACTGCTTTATTTGAAACGACATCGTTTTGGTTATGAAAAATTCATG<br>CCACCGAGTAGCGACGTCAACTTTAATTTTTACAAAAATGTTAAATTAGTGTATTTTATCATTTTTGAAGTTGGCGTGCAAAATAAAATATTGGCGAAAATCGGTAT<br>TATTTTAACGCAAATAACCCCTTCAAATAATTCATAGGAAATCTTGAATTCGAAACTATTTAGTACTCTTCTTCATTTTTCTCATCTTAGTAGCATTATACCATCATAT<br>TTCGCAGATAAAAGACTTTGCCATCGACATCATAAGACGAAGCTCGAGCACTTGGGTTTCATCCATGGCTACCACCCTCGATGACATGTGGGATTCCCTCGAGTCCC<br>AGCTTGCCGCCGCCGGCGATGGAGGCTCCGTCAACTACATTTTCCCTCTCCAAGAATTCCTCTTCGCCTTCCTCAGCCTCAACATCCTCGGCGCCAACGCCTCTGCCT<br>TCCCGAGATCAAGAAATCGGGCCACATA
SmAOC1	GGAGTGGGTTGGGCCTTTGAGTGGTCTTTGTGGGCCAATAAGAAATAACAGCTGCTAAATTGGTGCATCGGCCATGGTAACTCATTAATAATTTTGCTAGTGATAAC<br>TGATTAATAATCTTGAATTTATAATTAGATCTATGAATTCAAAATTTATTGTTCTTGAATTCACAATCAGATCTATGAAATCACAATTTAATGTTCTTGAATTCACAA<br>CTAAAACTAGAATTCACAATCAGTTTATGAATTCACAACTATATATAGTGTTACTGTGTTAGTCGTGAATTTATGTTTTAAAGCCTGAATTCACGACTAACAATATTT<br>CTAGTTGTGAATTCACGCTTTAAAACTTGAATTCACAACTAACACTATTTATAGTTGTGAATTCACGACTAACACTATTTTTAGTTCTGAATTCAAACTTTAAAAGTC<br>GAATTCACAATCAATATTAGCTATTCAGTTGTAAATTCGAGTTTTAAAACTTGAATTCACAACTAAAATTAATGTTACTTGTGAATTTCACTGGTTGTGAATTCGCGG<br>GTAATTTTTAAATTTTTTATATGCAAAAGTAAAATGATAAGTGTGATCAATTTTTAAATTTTTTTTATTTTAATGGTCAATTTTTAAATTTACTATTCCTAAGTAGCTA<br>TTTTAAAAATCCACTCATTTATAAATAAACTTTTAATTTTATTATAAACTAAATAAAATTTCCAGTTAATTTGAAAGTTATGTTTATTATAGATTTTGTGAAAAATAG<br>AAGCATGTGTAAAGCAAGAATATTTAAGTATTTCCACACGTAACGACCGCCGCCGACGACCATCTCCATACACATGGGCCGCACGTGGAACAATGAGTTTCCTCGC<br>AAGTGAATTCTAATTCTTCCCCACAACACAACACAATACAGTGAATACACCACAACACACCTCTCTCTTCTCTTCCTATATATACATACGCACCTGCATAGCCCAAA<br>CGGAATCTTTGTCTCGAGGCTTTCATCATTT
SmAOC2	ATAATTTGTAATCATTTTTTATTTAATTTTAATTTATTATACTTTTATACTCCCTCCGTCCCCTGAAGCGTGACCCATTTGTTTTCGGCACGAGAATTAAGAAATTTGT<br>ATTTTGTGTGTTAAGTGTGGTAGGTGAAAAGGTGAATAAAGGGTAAATTTTTTACTATTTTTAAAAACAAGTCAAGCTTCGTGGGACAACCCAAAAAGGAAAGTGG<br>GTCACGCTTTGCGGGACGGAGGGAGTATAATATAAAGATAATTAACAAGGGTTCACTCATCCATTTGGGGTTTATAATTATTTTTTTTATATTTATTTGAATTAATAA<br>TAGATTATGTGGGTTCATTAATTCAAAGTTAGGGTATATAATTTTTAAAATTTTAATTTTTCAATAATAAATACTAATTAGAGTTTATAATATAATAATAATTTTTAT<br>TTTTATAAAAAATAATTTATAAAATGAAGAAATGAAGAATAGTACACACTATATTTTAGGACAGATGATCTCATTTGCAATATTGAAGCCCACAAAGCGTGGATTA<br>ACATTACTCAAATTATTAAAATAAATTTTATTAAAAAGAAAAAACTAAAGAAACCCTTGAAAGCACAAAAAATAGACTAAATGCCGAGCATCATTAAAATCTCATT<br>AAAGAAAACCCGAAAGTAAAAAGCTTTAGCAAAAAAAAGAGTGCTAGTCTAAATAACAATGTTAACAAGTGAGTTGAGGCTATATTACTCAAATATTTATACGGC<br>AAAAACTAAAATAAGAAATTGCGTTGAGAAGTTTGGAAGTGGTGTGCGGCCGCACATGGTCGCACCGTCCATGAAAAACATTAATTTCAGATTGAGCCCACCACTA<br>CTTTTTTGTTGCTACTCATTTTCCATGCTCCTATAATTACATCTATCTATATATATTACTCCCAACAATCAAATACCATTCATTCAACATTTCCTCCGACAGCAGAGAG<br>AGAGAGAGAAAGAAAAAGGTGAATCAATTAATTAGCC
SmOPR3-1	GCCTTCTATTTTTTTTTTTAATATACAACCACTATTAGCACATTAAATTATTTAATATGATAAAATTCGATAATATAAAAATACAATATCTGTATATCTTACGGGATG<br>AACGCTAATTAGGTATCAAAGCAAATCTCCCCTAGGTTTTTCCATCTGATCTTGATGTTGACAAATTTGTTAGCATTTGGAAATAGGAAAAAAAAAAAAAGATAAGG<br>AAATATTTAAATAATTTAGTTAGATTAGGATAAAGCTAAAAAAAATGTTGAATATCAAGGAGATTTAGTATCAATTCAAATTACATTCCTAAATTTATTAGAATAAA<br>GTGTATATAGAAAAAAAAAATGAAGTAAAAAGAACGAATCATTGCGCGGTAGAATTTGAAAAGAAAGAAGAATAAATAAATGCCTTCTGAAGAAAGATATAGAC<br>CGATGGGATTTAACACTGTAAACTGCTGAGTACTTTTGAAAGCTTTGATACGTGGATTTGATGTCTAATTTTAATTCAATGAAATTTTAATTTCTTAATTATTGTAAA<br>ATGGAAACTAGAATAAAACTAATTCAAAAATAAAATAAAATCTATTGCACCCATAGTGAGTGCAATACCATTGTGGGAGTGGGTGCAATAGAAGTGGGACCCATG<br>GTATTGCACCCACTATGGGTGCAAGCATTGTGGATGCTCTAAGAAAGATAGACCGATGTTATGAGTTNTTACGTCCACGTTTATATAATATTTTAAATCAGCGTGGA<br>GCAATAATTTGCATCGCCCACCAAATGGATGACTAACTGTTGACCACAGACAAATGGAATCGCCCACCAATTACTTTACTAGTTTGCATATGAAAATCGTGCGCAA<br>TACCAATGCTAAATTCAAAATGTAATTTATTTGGCCTACTCCACCCCACTAGTATTCATTATTCATTATCCTCCTCCGCCCCCGATCACCGTCCTCCTCCTCCCCTAAC<br>TTCACCGTCCACGCATACACCGCCCTCCTTTAAATAGTGA

Gene	front 1000bp sequence
SmOPR3-2	TCACACATAAAAAATCACATTAAAATTAAAAAATCAATATAGTTTGTGGTTTTATTTGCCAAAATCCCACAAAATATCAAATAAAAGATGTCCACATAAATTAACA<br>ATACAGATAAATGCAATTTTCGTTGAAATGCTTTTCTTTCCAAAACCAAATTCTCTTTTCCCATGAACCCCATCCGAAGGGGAATGAGATTGAGTGTCCCACAATCT<br>TATGTGTGTCATTGTGTCCCATGCTTATATCTATTATTTTAAAAATATTTTTTATAAAAATAAAATTTATTATAATACTATGAATTATATTTAATTTTTATTTCAAAAA<br>TTGAATTTTTAAAAAAGTATATACCATCACTTTGAATTTATCAACCTATTATTAGCTAATAAAAGTGAAATTAAATGATAAAAAATAATTATATACCCTAGATTGAT<br>GGAATAAACCCTAGTTAATAATCACAAAATTAAACTAAAGCATATAAAATTGAAATTGAAGAAAATATATATAAGAAATTAAACAGAATTGAAAGTGGGACACAA<br>AGTGAGACATAAAATGTGGTACACCGTACACTAAATCTCATTCCATCCGAAGGTCAAAGTAATAATTAAGTTTTAGTTAATACTAGAAGTAGAAGAGATTAATACT<br>ATATTTATTTATTTATTTTGGTTTGATACGATTAATACTAAAAATTAAAGCATCGAAAAATTCATAATACAATTTAGAACAGCAAATTAAAGCGCAAGAGCAGTGGC<br>GAATCTGATGTATCCGCCGCCTCTATAATTAATTTGTAGGTGGGCTCTCACACGTTACTTTCTCTTGCCCCTTTTTTGACGTAAAAATGGCGCGCATTCATATTAATA<br>ATTGAGAAAGCAACATGCAAGACAATGAGTCAATGATGTAATCGCCTACGCAATTGCCGCCACCAGCTTCAATAAATCCTCCTCTGTTTTACACAGTTGAACAGAG<br>CACCAAACAAGAATTCAAAAACAGAGCAAAGCTAGAGTT
SmOPR3-3	CAAGGGCGGCGGCCTTCCTGCCGTCACTTTGATTCGCCGGAGAAAGAGAGATAAACGACCGTCGATGAGTGTTGTTGACTGGTGCGTGAGAAAGAGAGGGAGGGA<br>AGGAGGAGGGTGAGATGATAGAGGGAGATGGAGAGGGGCCGGAAGCCATAGCTGCTGTAACGGTGAGATGAGCGAGGGAGATGAAGAGGGGCCGGAAGCCATG<br>GCTGCTGTAACAGAGAGAGAAACGATGGGTGGCTGTGAAGAGAGAGAAAAACCATTGGAGAGAGAAACTATTGGTAGGTGAGAGAAAAGAGTTAGGATTTAGGT<br>TTCTATAGAACCTTAATTAATTCATGTTAATTTTAGCCCAAAATAGAAATGTGTCTTGTTTAATGGGACAGCCCGAAAAGGAAAGTGTGTCTTAAATAATGGGACGG<br>AGGGAGTACATTTTATGCAGTGACCCTAAATCTTAAATTTTCATTTTTCGGGTAAGTCCATCAAGTTATAAAGTTAACAAAAAACATATAGTATAATATAGTAAATA<br>AATTTTTTATAATTATATTTTTGTAATAAAAGAATAAATTATATTTTCTCAATTTAAATAGTTATAGACTTAATAAGTAAAAAAAATAAAATTCAATTGATCAGGCC<br>AATCCACTTGATTTTTTGCTCCGATTATTTTTAGTTTGGACCATTCAAACTTTCTTGTTTGGAGCTAAGAACTTTCGATCTTAATTCTCATTTTTACTTGATCTACTCGG<br>GCTGACCAGAAAAGGTTGACTTTGAATTAAACCGGCAGCCTTAGCTCTCTCACACGTCACTTTCCCTTAGACCTTTGATTTCAAAATGGCGCGCATAAATGATTGAG<br>GAAAGTAATATCGAAGACAATGATGTAATCGCCTACGCAATTGTCGCAGCTTCTGCAACTACTATAAATCCCTCCTCTGTTTTACACGGTTGAACAAGAATTAATTA<br>AAAAACTAGACAAAGTTAAGAGATAGAATTAATCTCAAGAGTT
SmOPR3-4	TAATAAATTTATAAATTAATTAATTCAAAATTAATATCAATTAGAGTTCATTATAAAATAAAGTGTTAATTTGTTTTAAATCCATAAACTTTACACAATGTTTGGTTT<br>TTCCCAAAATAAATAAAAATGTGTTTTAAATACAAAAACTTTCACACATTTTGGAATTTCCCACAAAAGTTAATTCAGATAAAATTAAATCTGTCTTAGAGTCGGAT<br>TTTGTTGTCGTGTAATATCTTAAAGGTTTAATTTAGTACAATTTATTTCACAATAATTAAAATACCCAAATTTTATATATAACATCATATATATCTTTTACGTCAGCA<br>AAATTCAGCGTCACACAAGATTCAATTTTGTCTGAATTGACTTTCATGGGAAATTCCAAAAATTATGAAAGTTCATGTATTTAGAACAAACTTTTATTTATTTTGTGA<br>AAAACCAAAAACCGTGTAAAGATTATGTATTTAAAACAAATTAACCCTAAAATAAATTGTATGCATGACAGATACCAAAAAAAGAAATACTCCCTCCGTCCCGGCC<br>AAGACGCTATATTTGCTTTTCGGCAGGAGTTGTAGATTAATGTTTTAAGTGTGTAATAATAAAGTGATAATGTTTTAAGGAGTTGTAGATTAATGTTTTAATGTTTTA<br>AGGAGTTGAAGGAATACATTGTATTTGTATGTTTCCAAATGATTTATTATTGTGTTTGGTAATGGTTTTACCAAGTTACAGTTTGTGTTGTACTCCTATTATTTTTTAT<br>TTGAACTCCTTTTTGATCGCTTCTTCAACTAATTCGATGGGCTAGCCTAAAGTCCGAACGTTTGGGGTTAAGATTCAACATTTTAACTCAAAAAAAAATCAATCCAA<br>TTTCTCCGTATCCAAATATTTATGTTATTCCTCTGCTCACCCCCCACAAGATGGGGGAATTCACACTCTCCCACAGGTTTAATTCCTTAATTCATTACATATATTTCTT<br>CTAATTCTTCCATTAATTGGGTGGTGTTGA
SmOPR3-5	TTATAAAAGATAATTAAGGCTCGTTTACTATGTAGTATGAAATAATGTGTGATATTTATTAACATATTAGATATTAATATTTTATAGGTATCTATCACATCTTTATCA<br>TGCATAACAAACAAATTCTTAGATATTAAATGGACATGTCCTCAAGGGGACACCAAGCGGTGCGACCTCCTTTCTGCGAACAGTGAGGTCTCGAGTTCAAATCTCA<br>CTGCCCCCCTCCCCAACTCCCACTAGTAAATTAATCATACGAGTCAAATTGTTGTCATATCAATTTAATAATGATCCATCTTATTACAATTAAACTTGTTTAGGATAT<br>GCTTGATTTGAATACGAACTCAATACAAATGATCCAAATTCAAATACAACTCGAATATGATATGATATCTTTTGTATTGATATGATATAATAATGACACGATTAAAA<br>TTTAAATTTATACACATGATAACGATATAAAGTTTGTGTATATAGACGCTAATAATAATAATAATAATAATAATAATAATAATTGCGATTTTACATAATAAATTCAG<br>CTGCAAATCTAAAAGAAAATACAAAATATTATTGAAAAATATTCCCTCCGTCTCATGAAGCATGACACAGTTCTTTTCGGCACGGGAATTAAGAAATTGGTATTTTA<br>TGTGTTAAGTGTGGTAGGTGAAAAAGTGAATAAAAGGTAAATTTTTTGCTATTTTTAGAAACGTGTCATGTTTTGTGGGATAGACCAAAAAGAAAACTGTGTCATG<br>CTTCGTGGGACGGAGGAGTATTCAACATGCAACTTATTCAAATTAAATTAAATTCTCCACTTCAAATAACAGCCACAAATTACTATCATACGAAGCAGCCCCCAGA<br>GTGAGTATACTAACATGTATAGCTTCGGCGAATTCAATGTAGATTGCTGCCGTCAGCATCGAATCAGCAGACTCCCATGACTAATTACATTGTCATATTTTCTCGAA<br>AATCTTTACGTTTTCAACCTCCATAAATACAGAATCAA

Table S7. Putative cis-acting elements identified in the promoter regions of JA pathway genes "*" means the gene contain this cis-acting element in the promoter region.

Gene Name	SmLOX1	SmLOX2	SmLOX3	SmLOX4	SmLOX5	SmLOX6	SmLOX7	SmLOX8	SmLOX9
AAGAA-motif	*	*	*	*	*			*	
A-box							*		
ABRE	*	*	*		*			*	*
ABRE3a			*		*				
ABRE4			*		*				
ACE									
AC-I									
AE-box						*			
ARE		*	*		*	*			
as-1		*	*		*	*			*
AT~TATA-box	*	*	*	*		*	*	*	
AT1-motif								*	
ATC-motif									*
AT-rich sequence							*		
AuxRR-core									
Box 4		*	*	*	*	*	*	*	*
box S									
CAAT-box	*	*	*	*	*	*	*	*	*
CARE									
CAT-box									
CCAAT-box						*			
CCGTCC motif							*		
CCGTCC-box							*		
CGTCA-motif		*	*		*	*			*
chs-CMA1a			*						
chs-CMA2a									
circadian									
CTAG-motif								*	
DRE core									
ERE			*			*		*	*
GA-motif							*		
Gap-box					*				
GARE-motif						*			
GATA-motif			*	*		*			*
G-Box	*	*	*		*			*	*
GC-motif									
GCN4_motif									
GT1-motif		*	*						
I-box					*	*			
LAMP-element			*					*	

Gene Name	SmLOX1	SmLOX2	SmLOX3	SmLOX4	SmLOX5	SmLOX6	SmLOX7	SmLOX8	SmLOX9
L-box									
LS7									
LTR		*	*				*		
MBS		*	*						
MBSI									
MRE							*		
Myb		*	*						
MYB						*	*		*
MYB recognition site						*			
Myb-binding site		*	*			*			*
MYB-like sequence			*			*			
MYC	*		*	*	*	*	*	*	*
Myc									
O2-site					*				
P-box					*			*	*
Pc-CMA2c						*			
plant_AP-2-like		*							
RY-element									
Sp1							*	*	
STRE			*	*		*		*	*
TATA		*					*		*
TATA-box	*	*	*	*	*	*	*	*	*
TATC-box		*			*	*			
TCA		*							
TCA-element		*					*		
TCCC-motif			*	*		*			
TCT-motif		*						*	
TGA-box									
TGACG-motif		*	*		*	*			*
TGA-element		*							
Unnamed__1	*	*	*	*	*	*		*	
Unnamed__4	*	*	*	*	*	*	*	*	*
Unnamed__6				*					
W box	*				*		*		
WRE3									
WUN-motif	*	*	*				*		
Y-box			*						

Table S7 (continued 1)

Gene Name	SmAOS1	SmAOS2	SmAOS3	SmAOS4	SmAOS5	SmAOS6	SmAOS7
AAGAA-motif		*	*		*		
A-box		*		*			
ABRE			*	*	*	*	*
ABRE3a			*		*		
ABRE4			*		*		
ACE							
AC-I							
AE-box			*		*		
ARE	*						*
as-1			*		*		*
AT~TATA-box	*		*	*	*	*	
AT1-motif		*					
ATC-motif							
AT-rich sequence							
AuxRR-core				*			
Box 4	*	*	*	*	*	*	*
box S		*					
CAAT-box	*	*	*	*	*	*	*
CARE							
CAT-box	*	*		*		*	
CCAAT-box							
CCGTCC motif		*		*			
CCGTCC-box		*		*			
CGTCA-motif			*		*		*
chs-CMA1a							
chs-CMA2a	*						
circadian							
CTAG-motif							
DRE core				*		*	
ERE	*					*	
GA-motif							
Gap-box							
GARE-motif							
GATA-motif						*	
G-Box			*	*	*	*	*
GC-motif							
GCN4_motif							
GT1-motif			*	*	*	*	
I-box			*		*		
LAMP-element							
L-box							

Gene Name	SmAOS1	SmAOS2	SmAOS3	SmAOS4	SmAOS5	SmAOS6	SmAOS7
LS7							
LTR						*	
MBS		*		*			
MBSI							*
MRE	*						
Myb	*						
MYB		*	*	*	*	*	*
MYB recognition site							
Myb-binding site		*				*	
MYB-like sequence			*	*	*		*
MYC	*	*	*	*	*	*	*
Myc							
O2-site							*
P-box							*
Pc-CMA2c							
plant_AP-2-like							
RY-element	*		*		*		
Sp1							
STRE		*	*	*	*		*
TATA	*	*				*	
TATA-box	*	*	*	*	*	*	*
TATC-box							
TCA							*
TCA-element		*				*	
TCCC-motif		*					
TCT-motif							
TGA-box							
TGACG-motif			*		*		*
TGA-element							*
Unnamed__1			*	*	*	*	
Unnamed__4	*	*		*		*	*
Unnamed__6	*			*			
W box							
WRE3		*					
WUN-motif							
Y-box							

Table S7 (continued 2)

Gene Name	SmAOC1	SmAOC2	SmOPR3-1	SmOPR3-2	SmOPR3-3	SmOPR3-4	SmOPR3-5
AAGAA-motif		*	*	*			
A-box		*	*		*	*	*
ABRE	*		*	*	*		*
ABRE3a	*		*				
ABRE4	*		*				
ACE							*
AC-I					*		
AE-box							
ARE				*	*	*	
as-1				*	*	*	*
AT~TATA-box	*	*	*	*		*	*
AT1-motif		*		*		*	
ATC-motif							
AT-rich sequence							
AuxRR-core							
Box 4	*	*		*	*	*	*
box S					*		
CAAT-box	*	*	*	*	*	*	*
CARE		*					*
CAT-box				*			
CCAAT-box							
CCGTCC motif		*	*		*	*	*
CCGTCC-box		*	*		*	*	*
CGTCA-motif				*	*	*	*
chs-CMA1a							
chs-CMA2a							
circadian							*
CTAG-motif	*						
DRE core	*						
ERE	*	*	*	*			*
GA-motif							
Gap-box							
GARE-motif							
GATA-motif			*				
G-Box	*		*		*	*	*
GC-motif			*				
GCN4_motif				*			
GT1-motif						*	
I-box			*			*	
LAMP-element							*
L-box				*			

Gene Name	SmAOC1	SmAOC2	SmOPR3-1	SmOPR3-2	SmOPR3-3	SmOPR3-4	SmOPR3-5
LS7						*	
LTR		*			*	*	*
MBS	*			*			
MBSI							
MRE					*		
Myb	*		*	*			
MYB							
MYB recognition site							
Myb-binding site			*				
MYB-like sequence							
MYC	*	*	*	*		*	*
Myc					*		
O2-site		*					
P-box							
Pc-CMA2c							
plant_AP-2-like							
RY-element							
Sp1			*		*		
STRE		*	*	*	*		*
TATA		*		*		*	*
TATA-box	*	*	*	*	*	*	*
TATC-box							*
TCA							
TCA-element							*
TCCC-motif					*		
TCT-motif			*				
TGA-box				*		*	
TGACG-motif				*	*	*	*
TGA-element	*				*		
Unnamed__1	*	*	*				*
Unnamed__4	*	*	*	*	*	*	*
Unnamed__6		*					*
W box	*		*	*			
WRE3				*			
WUN-motif		*					*
Y-box							

Table S8. Functional classification statistics of cis-acting elements

Category	Cis-acting elements	Number of genes	Function
**Transcription**	TATA-box	23	core promoter element around -30 of transcription start
	CAAT-box	23	common cis-acting element in promoter and enhancer regions
	A-box	8	cis-acting regulatory element
	circadian	1	cis-acting regulatory element involved in circadian control
**Develpoment**	GCN4-motif	1	cis-regulatory element involved in endosperm expression
	CCGTCC-box	8	cis-acting regulatory element related to meristem specific activation
	CAT-box	5	cis-acting regulatory element related to meristem expression
	RY-element	3	cis-acting regulatory element involved in seed-specific regulation
	AC-I	1	cis-acting regulatory element related to meristem specific activation
**Hormone**	ABRE	16	cis-acting element involved in the abscisic acid responsiveness
	GARE-motif	1	gibberellin-responsive element
	P-box	4	gibberellin-responsive element
	TATC-box	4	cis-acting element involved in gibberellin-responsiveness
	TCA-element	5	cis-acting element involved in salicylic acid responsiveness
	TGA-element	4	auxin-responsive element
	AuxRR-core	1	cis-acting regulatory element involved in auxin responsiveness
	TGA-box	2	part of an auxin-responsive element
	O2-site	3	cis-acting regulatory element involved in zein metabolism regulation
	ERE	11	ethylene-responsive element
	CGTCA-motif	12	cis-acting regulatory element involved in the MeJA-responsiveness
	TGACG-motif	12	cis-acting regulatory element involved in the MeJA-responsiveness
**Abiotic/ biotic stress**	ARE	9	cis-acting regulatory element essential for the anaerobic induction
	GC-motif	1	enhancer-like element involved in anoxic

Category	Cis-acting elements	Number of genes	Function
			specific inducibility
	AT-rich sequence	1	element for maximal elicitor-mediated activation (2copies)
	W box	7	elicitor-responsive element
	box S	2	elicitor-responsive element
	WUN-motif	6	wound-responsive element
	LTR	8	cis-acting element involved in low-temperature responsiveness
	MBS	6	MYB binding site involved in drought-inducibility
	CCAAT-box	1	MYBHv1 binding site
	MBSI	1	MYB binding site involved in flavonoid biosynthetic genes regulation
	MRE	3	MYB binding site involved in light responsiveness
	G-box	17	cis-acting regulatory element involved in light responsiveness
	Sp1	4	light responsive element
	I-box	6	part of a light responsive element
	GT1-motif	7	light responsive element
	ACE	1	cis-acting element involved in light responsiveness
	Box 4	21	part of a conserved DNA module involved in light responsiveness
	GATA-moti f	3	part of a light responsive element
	GA-motif	1	part of a light responsive element
	AE-box	3	part of a module for light response
	TCT-motif	3	part of a light responsive element
	TCCC-motif	5	part of a light responsive element
	ATC-motif	1	part of a conserved DNA module involved in light responsiveness
	LAMP-elem ent	3	part of a light responsive element
	chs-CMA2a	1	part of a light responsive element
	L-box	1	part of a light responsive element
	Gap-box	1	part of a light responsive element
	AT1-motif	5	part of a light responsive module
	chs-CMA1a	1	part of a light responsive element

Table S9. GO functional annotation results of JA gene family

Gene Name	Biological Process (BP)	Molecular Function (MF)	Cellular Component (CC)
SmLOX1	GO:0031408 oxylipin biosynthetic process GO:0006633 fatty acid biosynthetic process GO:0034440 lipid oxidation GO:0034052 positive regulation of plant-type hypersensitive response GO:0010311 lateral root formation GO:0009753 response to jasmonic acid GO:0009751 response to salicylic acid GO:0009737 response to abscisic acid GO:0009617 response to bacterium	GO:0016702 oxidoreductase activity, acting on single donors with incorporation of molecular oxygen, incorporation of two atoms of oxygen GO:0046872 metal ion binding	GO:0005737 cytoplasm GO:0043231 intracellular membrane-bounded organelle
SmLOX2	GO:0031408 oxylipin biosynthetic process GO:0006633 fatty acid biosynthetic process GO:0034440 lipid oxidation GO:1900366 negative regulation of defense response to insect GO:0010311 lateral root formation	GO:0016702 oxidoreductase activity, acting on single donors with incorporation of molecular oxygen, incorporation of two atoms of oxygen GO:0046872 metal ion binding	GO:0005737cytoplasm
SmLOX3	GO:0031408 oxylipin biosynthetic process GO:0006633 fatty acid biosynthetic process GO:0034440 lipid oxidation GO:1900366 negative regulation of defense response to insect GO:0010311 lateral root formation	GO:0016702 oxidoreductase activity, acting on single donors with incorporation of molecular oxygen, incorporation of two atoms of oxygen GO:0046872 metal ion binding	GO:0005737 cytoplasm

Gene Name	Biological Process (BP)	Molecular Function (MF)	Cellular Component (CC)
SmLOX4	GO:0031408 oxylipin biosynthetic process<br>GO:0006633 fatty acid biosynthetic process<br>GO:0034440 lipid oxidation	GO:0016702 oxidoreductase activity, acting on single donors with incorporation of molecular oxygen, incorporation of two atoms of oxygen<br>GO:0046872 metal ion binding	GO:0005738 cytoplasm
SmLOX5	GO:0031408 oxylipin biosynthetic process<br>GO:0006633 fatty acid biosynthetic process<br>GO:0034440 lipid oxidation	GO:0016702 oxidoreductase activity, acting on single donors with incorporation of molecular oxygen, incorporation of two atoms of oxygen<br>GO:0046872 metal ion binding	GO:0005739 cytoplasm
SmLOX6	GO:0031408 oxylipin biosynthetic process<br>GO:0006633 fatty acid biosynthetic process<br>GO:0034440 lipid oxidation<br>GO:0009695 jasmonic acid biosynthetic process<br>GO:0009611 response to wounding	GO:0016702 oxidoreductase activity, acting on single donors with incorporation of molecular oxygen, incorporation of two atoms of oxygen<br>GO:0046872 metal ion binding	GO:0005740 cytoplasm
SmLOX7	GO:0031408 oxylipin biosynthetic process<br>GO:0006633 fatty acid biosynthetic process<br>GO:0034440 lipid oxidation	GO:0016702 oxidoreductase activity, acting on single donors with incorporation of molecular oxygen, incorporation of two atoms of oxygen<br>GO:0046872 metal ion binding	GO:0005741 cytoplasm
SmLOX8	GO:0031408 oxylipin biosynthetic process<br>GO:0006633 fatty acid biosynthetic process<br>GO:0034440 lipid oxidation<br>GO:0009695 jasmonic acid biosynthetic process	GO:0016702 oxidoreductase activity, acting on single donors with incorporation of molecular oxygen, incorporation of two atoms of oxygen<br>GO:0046872 metal ion binding	GO:0005737 cytoplasm<br>GO:0009579 thylakoid<br>GO:0031984 organelle subcompartment<br>GO:0043231 intracellular membrane-bounded organelle
SmLOX9	GO:0031408 oxylipin biosynthetic process<br>GO:0006633 fatty acid biosynthetic process<br>GO:0034440 lipid oxidation	GO:0016702 oxidoreductase activity, acting on single donors with incorporation of molecular oxygen, incorporation of two atoms of oxygen<br>GO:0046872 metal ion binding	

Gene Name	Biological Process (BP)	Molecular Function (MF)	Cellular Component (CC)
SmAOS1	GO:0009695 jasmonic acid biosynthetic process<br>GO:0016125 sterol metabolic process<br>GO:0031407 oxylipin metabolic process<br>GO:0009753 response to jasmonic acid<br>GO:0006952 defense response<br>GO:0009620 response to fungus<br>GO:0009611 response to wounding<br>GO:0006633 fatty acid biosynthetic process	GO:0016705 oxidoreductase activity, acting on paired donors, with incorporation or reduction of molecular oxygen<br>GO:0004497 monooxygenase activity<br>GO:0005506 iron ion binding<br>GO:0020037 heme binding<br>GO:0009978 allene oxide synthase activity	GO:0009507chloroplast
SmAOS2	GO:0009695 jasmonic acid biosynthetic process<br>GO:0016125 sterol metabolic process<br>GO:0031407 oxylipin metabolic process<br>GO:0009753 response to jasmonic acid<br>GO:0050832 defense response to fungus<br>GO:0009611 response to wounding<br>GO:0006633 fatty acid biosynthetic process	GO:0016705 oxidoreductase activity, acting on paired donors, with incorporation or reduction of molecular oxygen<br>GO:0004497 monooxygenase activity<br>GO:0005506 iron ion binding<br>GO:0020037 heme binding<br>GO:0009978 allene oxide synthase activity	GO:0009508chloroplast
SmAOS3	GO:0016125 sterol metabolic process	GO:0016705 oxidoreductase activity, acting on paired donors, with incorporation or reduction of molecular oxygen<br>GO:0004497 monooxygenase activity<br>GO:0005506 iron ion binding<br>GO:0020037 heme binding<br>GO:0016829 lyase activity	

Gene Name	Biological Process (BP)	Molecular Function (MF)	Cellular Component (CC)
SmAOS4	GO:0016126 sterol metabolic process	GO:0016705 oxidoreductase activity, acting on paired donors, with incorporation or reduction of molecular oxygen<br>GO:0004497 monooxygenase activity<br>GO:0005506 iron ion binding<br>GO:0020037 heme binding<br>GO:0016829 lyase activity	
SmAOS5	GO:0016125 sterol metabolic process<br>GO:0033075 isoquinoline alkaloid biosynthetic process<br>GO:0009695 jasmonic acid biosynthetic process	GO:0016705 oxidoreductase activity, acting on paired donors, with incorporation or reduction of molecular oxygen<br>GO:0004497 monooxygenase activity<br>GO:0005506 iron ion binding<br>GO:0020037 heme binding<br>GO:0016829 lyase activity	
SmAOS6	GO:0009695 jasmonic acid biosynthetic process<br>GO:0016125 sterol metabolic process	GO:0016705 oxidoreductase activity, acting on paired donors, with incorporation or reduction of molecular oxygen<br>GO:0004497 monooxygenase activity<br>GO:0005506 iron ion binding<br>GO:0020037 heme binding	
SmAOS7	GO:0016125 sterol metabolic process	GO:0016705 oxidoreductase activity, acting on paired donors, with incorporation or reduction of molecular oxygen<br>GO:0004497 monooxygenase activity<br>GO:0005506 iron ion binding<br>GO:0020037 heme binding<br>GO:0016829 lyase activity	GO:0016021 integral component of membrane

Gene Name	Biological Process (BP)	Molecular Function (MF)	Cellular Component (CC)
SmAOC1	GO:0009695 jasmonic acid biosynthetic process<br>GO:0033274 response to vitamin B2<br>GO:1900367 positive regulation of defense response to insect<br>GO:0009625 response to insect<br>GO:0080186 developmental vegetative growth<br>GO:0009864 induced systemic resistance, jasmonic acid mediated signaling pathway<br>GO:0010218 response to far red light<br>GO:0009646 response to absence of light<br>GO:0010114 response to red light<br>GO:0048573 photoperiodism, flowering	GO:0046423 allene-oxide cyclase activity	GO:0009507chloroplast
SmAOC2	GO:0009695 jasmonic acid biosynthetic process<br>GO:0033274 response to vitamin B2<br>GO:1900367 positive regulation of defense response to insect<br>GO:0009625 response to insect<br>GO:0080186 developmental vegetative growth<br>GO:0009864 induced systemic resistance, jasmonic acid mediated signaling pathway<br>GO:0010218 response to far red light<br>GO:0009646 response to absence of light<br>GO:0010114 response to red light<br>GO:0048573 photoperiodism, flowering	GO:0046423 allene-oxide cyclase activity	GO:0009507chloroplast

Gene Name	Biological Process (BP)	Molecular Function (MF)	Cellular Component (CC)
SmOPR3-1	GO:0009695 jasmonic acid biosynthetic process<br>GO:0031408 oxylipin biosynthetic process<br>GO:0010193 response to ozone<br>GO:0048443 stamen development<br>GO:0009620 response to fungus<br>GO:0006633 fatty acid biosynthetic process	GO:0010181 FMN binding<br>GO:0016629 12-oxophytodienoate reductase activity<br>GO:0042802 identical protein binding	GO:0005777 peroxisome<br>GO:0005829 cytosol
SmOPR3-2	GO:0031408 oxylipin biosynthetic process<br>GO:0006633 fatty acid biosynthetic process<br>GO:0009695 jasmonic acid biosynthetic process	GO:0010181 FMN binding<br>GO:0016491 oxidoreductase activity	
SmOPR3-3	GO:0031408 oxylipin biosynthetic process<br>GO:0006633 fatty acid biosynthetic process<br>GO:0009696 jasmonic acid biosynthetic process	GO:0010181 FMN binding<br>GO:0016491 oxidoreductase activity	
SmOPR3-4	GO:0031408 oxylipin biosynthetic process<br>GO:0006633 fatty acid biosynthetic process<br>GO:0009697 jasmonic acid biosynthetic process	GO:0010181 FMN binding<br>GO:0016491 oxidoreductase activity	
SmOPR3-5	GO:0031408 oxylipin biosynthetic process<br>GO:0006633 fatty acid biosynthetic process	GO:0010181 FMN binding<br>GO:0016629 12-oxophytodienoate reductase activity	

Table S10: Ka/Ks calculation of duplicate gene pairs in the JA gene family

Duplicated pairs	Ka	Ks	Ka/Ks	Purifying selection
SmLOX2-SmLOX3	0.166239585	1.162062986	0.143055572	Yes
SmLOX5-SmLOX9	0.139350744	0.176608134	0.789039218	Yes
SmAOS2-SmAOS6	0.075942509	0.087614086	0.86678424	Yes
SmAOS3-SmAOS5	0.032570612	0.033443083	0.973911778	Yes
SmOPR3-2-SmOPR3-3	0.051148777	0.111044956	0.460613245	Yes
